# Web-Based, Participant-Driven Studies Yield Novel Genetic Associations for Common Traits

**DOI:** 10.1371/journal.pgen.1000993

**Published:** 2010-06-24

**Authors:** Nicholas Eriksson, J. Michael Macpherson, Joyce Y. Tung, Lawrence S. Hon, Brian Naughton, Serge Saxonov, Linda Avey, Anne Wojcicki, Itsik Pe'er, Joanna Mountain

**Affiliations:** 1 23andMe, Mountain View, California, United States of America; 2 Department of Computer Science, Columbia University, New York, New York, United States of America; 3 Department of Anthropology, Stanford University, Stanford, California, United States of America; Georgia Institute of Technology, United States of America

## Abstract

Despite the recent rapid growth in genome-wide data, much of human variation remains entirely unexplained. A significant challenge in the pursuit of the genetic basis for variation in common human traits is the efficient, coordinated collection of genotype and phenotype data. We have developed a novel research framework that facilitates the parallel study of a wide assortment of traits within a single cohort. The approach takes advantage of the interactivity of the Web both to gather data and to present genetic information to research participants, while taking care to correct for the population structure inherent to this study design. Here we report initial results from a participant-driven study of 22 traits. Replications of associations (in the genes *OCA2*, *HERC2*, *SLC45A2*, *SLC24A4*, *IRF4*, *TYR*, *TYRP1*, *ASIP*, and *MC1R*) for hair color, eye color, and freckling validate the Web-based, self-reporting paradigm. The identification of novel associations for hair morphology (rs17646946, near *TCHH*; rs7349332, near *WNT10A*; and rs1556547, near *OFCC1*), freckling (rs2153271, in *BNC2*), the ability to smell the methanethiol produced after eating asparagus (rs4481887, near *OR2M7*), and photic sneeze reflex (rs10427255, near *ZEB2*, and rs11856995, near *NR2F2*) illustrates the power of the approach.

## Author Summary

Twin studies have shown that many human physical characteristics, such as hair curl, earlobe shape, and pigmentation are at least partly heritable. In order to identify the genes involved in such traits, we administered Web-based surveys to the customer base of 23andMe, a personal genetics company. Upon completion of surveys, participants were able to see how their answers compared to those of other customers. Our examination of 22 different common traits in nearly 10,000 participants revealed associations among several single-nucleotide polymorphisms (SNPs, a type of common DNA sequence variation) and freckling, hair curl, asparagus anosmia (the inability to detect certain urinary metabolites produced after eating asparagus), and photic sneeze reflex (the tendency to sneeze when entering bright light). Additionally our analysis verified the association of a large number of previously identified genes with variation in hair color, eye color, and freckling. Our analysis not only identified new genetic associations, but also showed that our novel way of doing research—collecting self-reported data over the Web from involved participants who also receive interpretations of their genetic data—is a viable alternative to traditional methods.

## Introduction

Many common human traits have long been understood to have a genetic basis, yet in only a few cases have influential genes been identified. Even pigmentation, for which almost 40 years ago Cavalli-Sforza [1] estimated that there were four genes underlying variation, has yielded associations only recently [2]–[5] (showing that pigmentation is rather polygenic [6]). We have conducted, within a novel, web-based research framework, genome-wide association studies of 22 common human traits. These traits were selected based on indications of heritability or a simple mode of inheritance, ease of phenotype data collection via web-based self-report, and broad interest.

**Figure 1:**
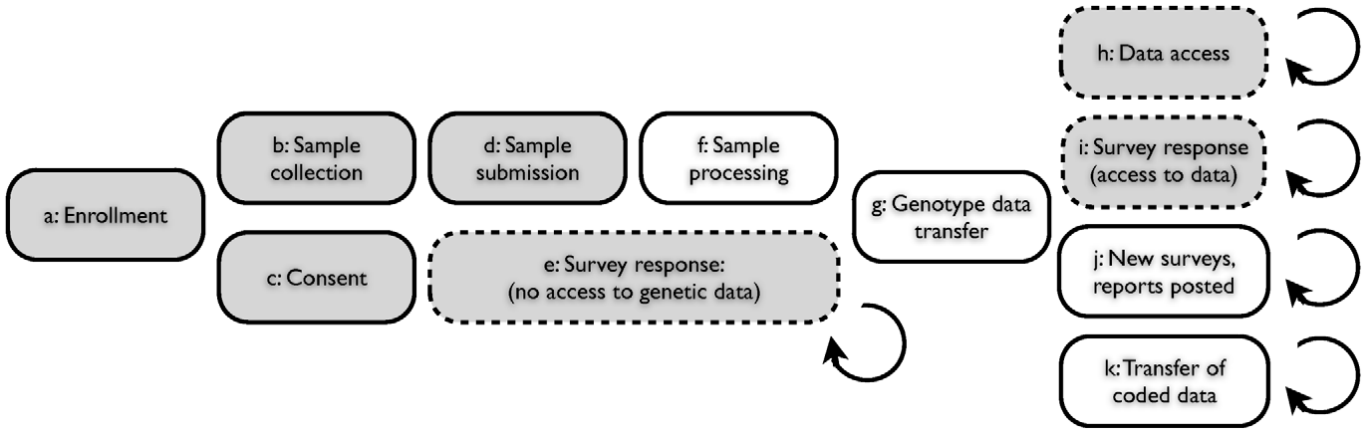
Web-based accrual of genotype and phenotype data via a personal genetic information service. (A) Individual participant signs up for service via web; (B) Participant receives sample collection kit; (C) Participant consents, via web, to use of anonymized genotype and survey responses for research; (D) Participant sends saliva sample to contracted lab; (E) Participant logs on to service web site with option to respond to one or more surveys, prior to having access to personal genetic data; (F) Laboratory processes sample, generating data for 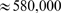 single nucleotide polymorphisms (SNPs); (G) Encrypted genotype data transferred from laboratory to secure server; (H) Participant logs on to service web site with option to access personal genetic data (both raw genotypes and customized reports); (I) Participant logs on and has the option to respond to one or more surveys; (J) New genetic reports posted, new surveys posted; (K) Genotype data and survey responses for individuals, coded and stripped of individually identifying information, are transferred to research team. Shaded boxes indicate participant actions; clear boxes indicate lab or service actions. Dashed boxes indicate optional participant actions within framework of service access.

Data for these studies was collected within a research framework wherein research participants, derived from the customer base of 23andMe, Inc., a direct-to-consumer genetic information company, consented to the use of their data for research and were provided with access to their personal genetic information (Figure 1). They were then given the option of contributing phenotype data via a series of web-based surveys. The result is a single, continually expanding cohort, containing a self-selected set of individuals who participate in multiple studies in parallel.

The parallel and continual nature of this research framework facilitates the rapid recruitment of participants to many studies at once. Furthermore, the presentation of interpreted genetic data to the participants creates incentive for them to return to the website, lowering the marginal cost of recontacting for additional analyses. The participant-driven nature of this study design and the resulting heterogeneity of the data sets require that care be taken to eliminate population stratification and other possible sources of bias. However, the challenges of eliminating such stratification and biases are balanced by the continuous accrual of new data as participants sign up and respond to new surveys.

From the initial set of surveys released, we report results on the 22 traits meeting our sample size criteria (over 1500 unrelated northern European respondents with the additional requirement of at least 500 cases for binary traits). The phenotypes that met these criteria are described below.

### Pigmentation

Pigmentation has been a fruitful area for genetic research since the 19th century, when scientists realized that the mice with varied coat colors that “mouse fanciers” had been developing for centuries provided easily tracked phenotypes for genetic analysis [7]. Many genetic variants underlying “normal” variation in human pigmentation have recently been discovered [2]–[5]. These variants account for a significant part of the known variation in pigmentation, (approximately 30% for hair color, 60% for eye color, see Results), but much remains unexplained.

### Hair morphology

Human hair varies in thickness as well as in the extent of curl, which is related to the shape of the hair (round versus flat cross-section). Over 100 years ago Davenport and Davenport [8] reported a study of hair morphology in families, concluding that straight hair was recessive to curly hair. More recently, a candidate gene approach discovered that *EDAR* is associated with hair thickness in Asians [9].

### Ability to smell the urinary metabolites of asparagus

The study of the ability to smell the urinary metabolites of asparagus (probably mostly methanethiol, a sulfur-containing compound) also dates back over 100 years [10]. Since then, authors have debated whether the variation among humans is in the ability to produce methanethiol (thought from family studies to be inherited in an autosomal dominant manner [10]) or in the ability to smell that compound [11]. No previous studies have reported genes or single nucleotide polymorphisms (SNPs) associated with this sensory ability.

### Photic sneeze

Listed under “ACHOO (Autosomal-dominant Compelling Helio-Ophthalmic Outburst) syndrome” in Online Mendelian Inheritance in Man (OMIM), the “photic sneeze reflex” refers to the tendency to sneeze when moving from relative darkness into bright light—most often sunlight. Aristotle discussed the trait in a section of his Book of Problems called “Problems concerning the nose,” hypothesizing that heat-generated movement led to tickling of the nose. No previous studies have reported genes or SNPs associated with this particular reflex.

### Other phenotypes

The other traits analyzed here fall into three broad categories. The first category consists of laterality preferences: handedness, footedness, ocular dominance, and hand-clasp (which thumb is on top when clasping one's hands). The second group consists of simple physical characteristics: whether participants have had cavities, have worn braces, have had wisdom teeth removed, have astigmatism, wear glasses, have attached earlobes, and suffer from motion sickness while riding in a car. The third group consists of personality traits and preferences: optimism, a preference for sweet versus salty food, and preference for night-time versus morning-time activity. None of these traits have well-established associations with SNPs, although handedness [12] and diurnal preference [13] have putative genetic associations.

## Results

**Figure 2:**
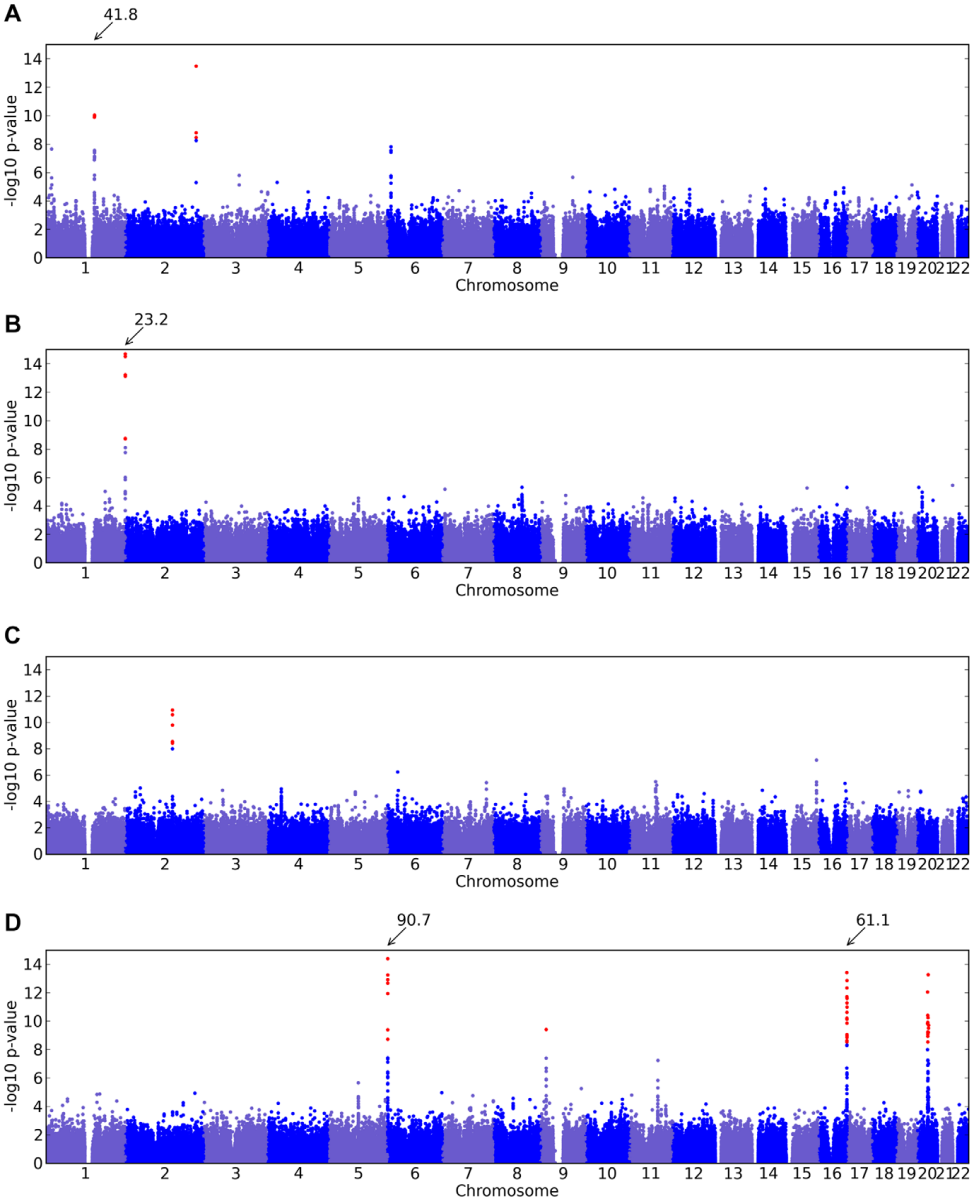
Manhattan plots for new associations. Shown are (A) hair curl, (B) asparagus anosmia, (C) photic sneeze reflex, and (D) freckling. Plots show scores (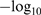 p-values) for all SNPs by physical position. All plots are trimmed at a maximum score of 15. For regions with a more significant association, the strongest score in that region is shown above the region.

**Figure 3:**
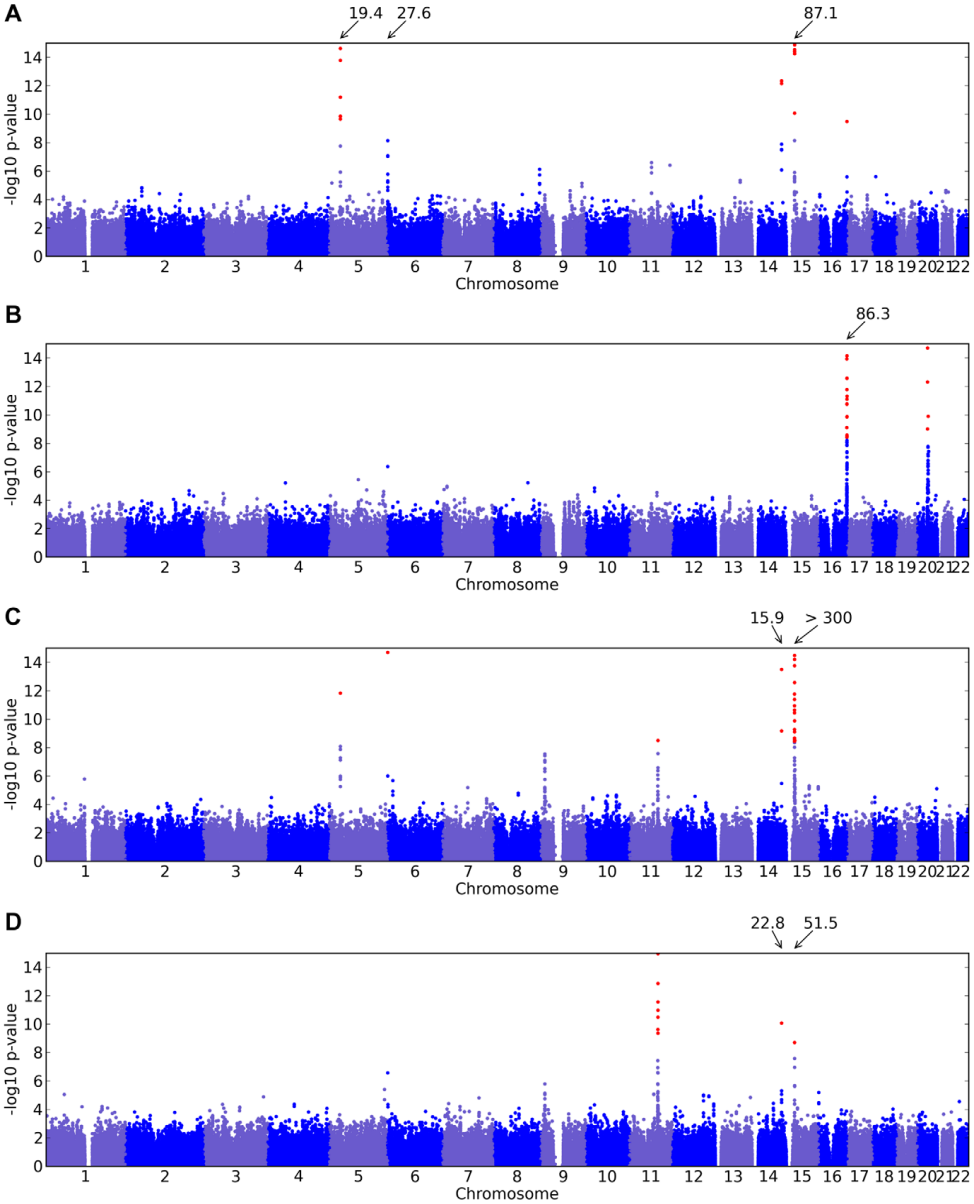
Manhattan plots for replications. Shown are (A) hair color, (B) red hair, (C) blue to brown eye color, and (D) green versus blue eye color. Plots show scores (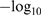 p-values) for all SNPs by physical position. All plots are trimmed at a maximum score of 15. For regions with a more significant association, the strongest score in that region is shown above the region.

**Figure 4:**
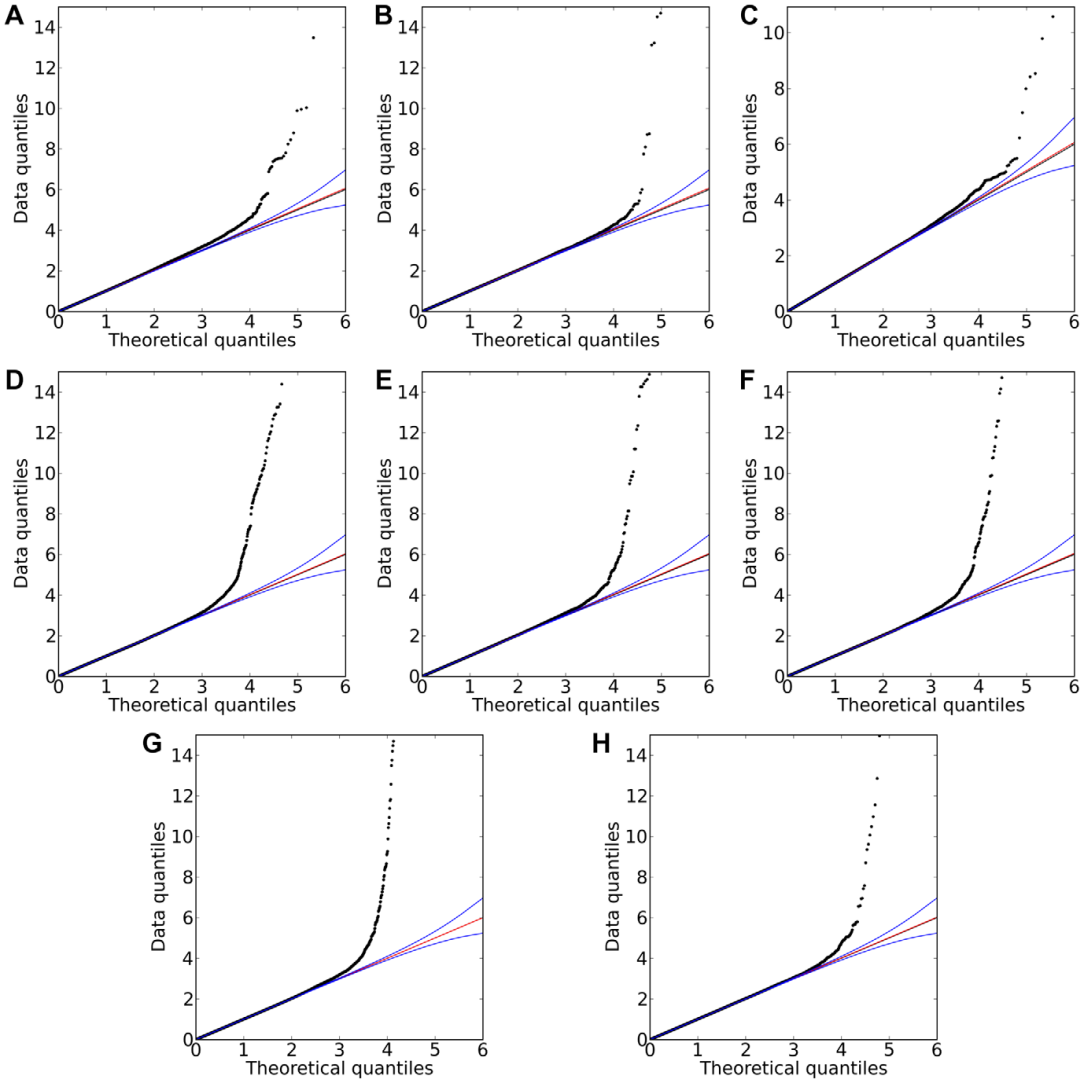
Quantile-quantile plots for new associations and replications. Shown are (A) hair curl, (B) asparagus anosmia, (C) photic sneeze reflex, (D) freckling, (E) hair color, (F) red hair, (G) blue to brown eye color, and (H) green versus blue eye color. All plots are trimmed at a maximum score of 15 to better show details. Approximate 95% CIs are indicated in blue. The red line passes through the median p-value.

Of the 22 studies, eight yielded positive results, with novel associations discovered for four traits and replications for five traits (Table 1). All five replications are for pigmentation-related traits. The novel associations reveal SNPs associated with hair morphology, detection of a urinary metabolite of asparagus, photic sneeze reflex, and freckling. Manhattan and qqplots for the novel associations and replications are shown in Figure 2, Figure 3, and Figure 4.

**Table 1:** Statistics on the 22 studies (with or without associations reaching genome-wide significance).

Phenotype	Size	Top hit	# Loci	Loci
Eye color, blue to brown	4402	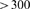	6	OCA2, SLC24A4, IRF4, SLC45A2, TYR, (TYRP1)
Freckles	4405	90.68	5	*IRF4*, MC1R, ASIP, **BNC2**, (TYR)
Hair color, blond to brown	3044	87.07	5	OCA2, IRF4, SLC45A2, SLC24A4, MC1R
Red hair	4422	86.28	2	MC1R, ASIP
Eye color, green/blue	2826	51.52	3	OCA2, SLC24A4, TYR
Hair curl	5385	41.80	3	TCHH, **WNT10A**, (**OFCC1**)
Asparagus anosmia	4742	23.18	1	**OR2M7**
Photic sneeze reflex	5390	10.93	2	**2q22.3**, (**NR2F2**)
Footedness	3079	6.75	0	
Attached earlobes	3915	6.59	0	
Morningness	4264	6.50	0	
Braces	4011	6.45	0	
Optimism	3936	6.29	0	
Astigmatism	7701	6.17	0	
Prefer sweet snacks	3100	6.07	0	
Wisdom teeth	3983	5.89	0	
Cavities	5366	5.81	0	
Glasses	5386	5.76	0	
Ocular dominance	3126	5.70	0	
Hand-clasp	5256	5.66	0	
Motion sickness	2987	5.55	0	
Handedness	4268	5.30	0	

Loci are called significant if they contain a SNP with 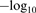 p-value over 8.4 and suggestively significant if they have one between 7.1 and 8.4. Loci that were not previously associated with the given trait are in bold, those where we report a remapping of a previous hit are in italics, and suggestively significant loci are in parentheses. Size refers to the total number of individuals in the study. The “top hit” refers to the largest 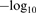 p-value for the given trait. The genomic control inflation factor, 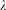, [59] was between 1.0 and 1.02 for all studies. For more details, including 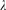, numbers of cases and controls, and covariates used in the analyses, see Supplementary Table 1 of Text S6.

The studies were performed on multiple overlapping datasets drawn from a single cohort. The cohort was derived from the subset of the customer base of 23andMe, Inc. who took surveys relevant to the 22 traits considered here. Of these individuals, only those assessed to be of northern European ancestry were included. In addition, individuals were eliminated until any pair of participants shared at most 700 cM of full or half identity by descent (IBD), approximately the lower end of sharing between a pair of first cousins. Average IBD between a pair of participants was 0.146cM, median IBD was 0 and only 123 pairs of individuals shared more than 100 cM. The resulting cohort consisted of a total of 9126 individuals who had answered at least one of the surveys considered here. Each individual was genotyped on the Illumina HumanHap550+ BeadChip platform (consisting of the HumanHap550 panel along with a custom set of approximately 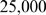 SNPs selected by 23andMe). After quality control (see Methods), 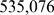 SNPs were used from this platform.

Phenotypes were collected using 13 surveys posted on the 23andMe website. From these surveys, 22 traits met our criteria for inclusion, which required over 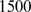 unrelated participants who responded to the relevant survey questions and were assessed to be of northern European origin. In addition, for binary phenotypes we required at least 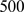 participants with each outcome before analysis. See Text S5 for full descriptions of the phenotypes.

Detailed summaries of results are shown in Table 2, Table 3, and Table 4. These summaries include the SNPs selected within each associated region to give the best predictive model while attempting not to over-fit (using a stepwise regression approach with the AIC, see Methods). The reader should be warned that this approach is anti-conservative about the number of effects fitted and, in particular, all SNPs appearing in these tables are not necessarily independently associated. Throughout, “score” refers to the negative 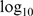 p-value for the association between a SNP and a trait. We also include Bayes factors (see Methods) in the tables and the plots of imputed and genotyped SNPs within each associated region due to their usefulness in comparing associations and their ability to incorporate uncertainty arising from imputation. Because we performed multiple (22) parallel studies, we used a very conservative threshold of 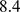 for a SNP before it was claimed to reach genome-wide significance (see Methods). Associations significant under this correction for a single study but not for all studies (that is, with scores between 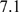 and 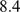) are called “suggestive” and are shown in Table 5.

**Table 2:** Selected SNPs reaching genome-wide significance for hair curl, asparagus anosmia, photic sneeze reflex, and freckling.

Phenotype	SNP	Chr	Position	Locus	Alleles	MAF	Score	BF	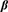	OR
**Hair curl**	rs17646946	1	150,329,391	TCHH	G/A	0.20	41.80	39.90	−0.294	—
	rs499697	1	150,759,778	LCE3E	A/G	0.29	9.95	7.61	0.126	—
	rs7349332	2	219,464,627	WNT10A	C/T	0.14	13.48	11.01	0.193	—
**Asparagus**	rs4481887	1	246,563,486	OR2M7	G/A	0.26	23.18	21.74	−0.514	0.598
	rs4309013	1	246,547,391	OR2M7	T/C	0.27	22.27	20.78	−0.496	0.609
	rs4244187	1	246,560,134	OR2M7	C/T	0.27	22.02	20.44	−0.493	0.611
**Sneeze**	rs10427255	2	145,841,993	2q22.3	T/C	0.46	10.93	8.65	0.280	1.323
**Freckling**	rs12203592	6	341,321	IRF4	C/T	0.18	90.68	87.04	1.611	—
	rs872071	6	356,064	IRF4	A/G	0.49	14.38	12.52	0.508	—
	rs3778607	6	348,799	IRF4	G/A	0.47	12.67	10.65	−0.471	—
	rs9328192	6	379,364	IRF4	A/G	0.50	9.39	7.11	0.395	—
	rs9405675	6	389,600	IRF4	A/G	0.36	8.72	6.86	−0.392	—
	rs12931267	16	88,346,233	MC1R	C/G	0.08	61.08	60.89	1.875	—
	rs8049897	16	88,551,703	MC1R	G/A	0.15	29.79	27.76	1.033	—
	rs1805008	16	88,513,645	MC1R	C/T	0.07	23.18	21.05	1.212	—
	rs1805009	16	88,514,047	MC1R	G/C	0.02	17.32	14.41	2.017	—
	rs11547464	16	88,513,592	MC1R	G/A	0.01	12.85	9.91	2.430	—
	rs11861084	16	88,403,211	MC1R	C/A	0.41	11.27	8.90	−0.445	—
	rs7204478	16	88,322,986	MC1R	C/T	0.45	10.61	8.40	0.427	—
	rs7195066	16	88,363,824	MC1R	C/T	0.32	10.12	7.76	−0.449	—
	rs11648785	16	88,612,062	MC1R	C/T	0.31	8.96	6.70	−0.424	—
	rs8060934	16	88,447,526	MC1R	T/C	0.48	8.86	6.60	−0.390	—
	rs154659	16	88,194,838	MC1R	T/C	0.25	8.62	6.45	0.433	—
	rs619865	20	33,331,111	ASIP	G/A	0.10	13.26	10.78	0.771	—
	rs4911442	20	32,818,707	ASIP	A/G	0.12	10.24	7.98	0.629	—
	rs291671	20	31,414,506	ASIP	A/G	0.10	12.04	9.89	0.772	—
	rs6088316	20	31,890,503	ASIP	A/G	0.17	10.40	8.37	0.568	—
	rs761238	20	31,983,649	ASIP	T/G	0.33	9.90	7.56	0.428	—
	rs17305657	20	31,270,249	ASIP	T/C	0.09	9.82	7.56	0.697	—
	rs4812405	20	34,709,999	ASIP	C/A	0.07	9.70	7.40	0.783	—
	rs1474976	20	34,195,786	ASIP	A/C	0.11	9.50	7.23	0.620	—
	rs4911414	20	32,193,105	ASIP	G/T	0.34	8.52	6.26	0.393	—
	rs2153271	9	16,854,521	BNC2	T/C	0.41	9.40	7.28	−0.402	—

“Score” is the negative 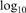 p-value for the (linear or logistic) regression coefficient 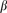 where genotypes were coded as 0, 1, or 2 according to the number of copies of the minor allele. Alleles are listed as major/minor. “OR” refers to the allelic odds ratio (for binary traits). “BF” is the 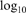 Bayes factor; position is the NCBI build 36 human assembly position. Hair curl was coded as a quantitative trait on a 6 point scale where zero was straight and five was “very tight curls.” “Sneeze” refers to photic sneeze reflex and “Asparagus” to asparagus anosmia, the lack of the ability to smell the urinary metabolites of asparagus. Freckling was coded as a quantitative trait on a 17 point scale where zero referred to the lack of freckles. Confidence intervals for ORs and 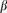 can be found in the tables of the Text S6. Displayed SNPs were selected among all significant hits using a stepwise regression procedure.

**Table 3:** Selected SNPs reaching genome-wide significance for replications of hair color associations.

Phenotype	SNP	Chr	Position	Locus	Alleles	MAF	Score	BF	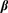	OR
**Hair color**	rs12913832	15	26,039,213	OCA2	G/A	0.23	87.07	85.76	0.974	—
	rs7183877	15	26,039,328	OCA2	C/A	0.08	21.15	18.47	0.759	—
	rs4778138	15	26,009,415	OCA2	A/G	0.12	14.40	11.83	0.510	—
	rs12203592	6	341,321	IRF4	C/T	0.18	27.64	27.17	0.589	—
	rs16891982	5	33,987,450	SLC45A2	G/C	0.03	19.41	16.76	1.104	—
	rs12896399	14	91,843,416	SLC24A4	G/T	0.44	12.34	9.83	−0.308	—
	rs12931267	16	88,346,233	MC1R	C/G	0.08	9.48	7.33	−0.556	—
**Red hair**	rs12931267	16	88,346,233	MC1R	C/G	0.08	86.28	89.80	0.556	—
	rs4238833	16	88,578,190	MC1R	T/G	0.37	42.77	41.02	0.228	—
	rs8049897	16	88,551,703	MC1R	G/A	0.15	42.38	40.65	0.309	—
	rs1805008	16	88,513,645	MC1R	C/T	0.07	37.48	36.19	0.387	—
	rs4408545	16	88,571,529	MC1R	C/T	0.50	24.23	22.14	−0.164	—
	rs1805009	16	88,514,047	MC1R	G/C	0.02	17.43	14.48	0.508	—
	rs2353033	16	87,913,062	MC1R	T/C	0.43	13.93	11.55	0.123	—
	rs2159116	16	88,359,011	MC1R	C/A	0.17	12.58	10.37	0.157	—
	rs7196459	16	88,668,978	MC1R	G/T	0.08	11.31	8.94	0.208	—
	rs7195066	16	88,363,824	MC1R	C/T	0.32	11.10	8.69	−0.118	—
	rs447735	16	88,261,850	MC1R	T/C	0.44	9.85	7.49	−0.103	—
	rs11648785	16	88,612,062	MC1R	C/T	0.31	8.47	6.21	−0.103	—
	rs4347628	16	88,098,136	MC1R	C/T	0.44	8.42	6.28	−0.095	—
	rs291671	20	31,414,506	ASIP	A/G	0.10	14.70	12.45	0.215	—
	rs17305657	20	31,270,249	ASIP	T/C	0.09	12.31	9.89	0.197	—
	rs619865	20	33,331,111	ASIP	G/A	0.10	9.89	7.59	0.165	—

See Table 2 for details. Hair color was coded on an eight point scale from light to dark. Red hair was coded on a four point scale based on the amount of red contained.

**Table 4:** Selected SNPs reaching genome-wide significance for replications of eye color associations.

Phenotype	SNP	Chr	Position	Locus	Alleles	MAF	Score	BF	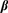	OR
**Eye color**	rs12913832	15	26,039,213	OCA2	G/A	0.23	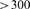	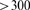	2.491	—
	rs8039195	15	26,189,679	OCA2	T/C	0.14	291.35	293.35	2.088	—
	rs16950987	15	26,199,823	OCA2	G/A	0.05	125.44	121.47	2.169	—
	rs2240204	15	26,167,627	OCA2	G/A	0.05	117.51	113.57	2.127	—
	rs6497253	15	25,962,144	OCA2	G/A	0.21	43.47	40.32	0.710	—
	rs1470608	15	25,961,716	OCA2	G/T	0.14	42.49	39.33	0.839	—
	rs4778232	15	25,955,360	OCA2	C/T	0.21	38.75	35.90	0.663	—
	rs1597196	15	25,968,517	OCA2	G/T	0.17	31.88	28.81	0.648	—
	rs7170451	15	25,865,819	OCA2	G/A	0.27	26.48	23.52	0.503	—
	rs1800407	15	25,903,913	OCA2	C/T	0.08	23.49	20.78	0.823	—
	rs4778220	15	25,894,733	OCA2	A/G	0.17	20.58	17.83	0.531	—
	rs728405	15	25,873,448	OCA2	A/C	0.22	19.91	17.34	0.466	—
	rs1800404	15	25,909,368	OCA2	T/C	0.21	14.47	11.91	0.411	—
	rs1107267	15	25,645,234	OCA2	G/A	0.26	11.76	10.33	0.338	—
	rs3930739	15	25,713,937	OCA2	C/T	0.46	11.38	8.96	−0.288	—
	rs17565953	15	25,692,250	OCA2	G/T	0.40	10.94	8.68	−0.289	—
	rs977588	15	25,852,901	OCA2	A/C	0.41	8.55	6.48	0.252	—
	rs12896399	14	91,843,416	SLC24A4	G/T	0.44	15.95	13.49	−0.343	—
	rs4904868	14	91,850,754	SLC24A4	C/T	0.45	13.49	10.95	0.317	—
	rs12203592	6	341,321	IRF4	C/T	0.18	14.69	13.26	−0.424	—
	rs16891982	5	33,987,450	SLC45A2	G/C	0.03	11.83	9.42	0.840	—
	rs1393350	11	88,650,694	TYR	G/A	0.27	8.49	6.25	−0.278	—
**Green eyes**	rs12913832	15	26,039,213	OCA2	G/A	0.23	51.52	41.34	2.131	8.425
	rs7183877	15	26,039,328	OCA2	C/A	0.08	29.54	21.57	2.275	9.732
	rs12896399	14	91,843,416	SLC24A4	G/T	0.44	22.82	19.49	−0.546	0.579
	rs1847134	11	88,644,901	TYR	A/C	0.32	14.95	13.13	−0.461	0.631
	rs1827430	11	88,658,088	TYR	A/G	0.38	10.97	8.93	−0.377	0.686
	rs11018528	11	88,570,025	TYR	A/G	0.30	10.48	8.73	−0.391	0.676
	rs7120151	11	88,380,027	TYR	G/A	0.29	9.61	8.19	−0.375	0.687
	rs1806319	11	88,677,584	TYR	T/C	0.37	9.35	7.37	−0.348	0.706

See Table 2 for details. Eye color was coded on a seven point scale from blue to brown. Green eye color was treated as a binary trait, where cases had green eyes and controls blue.

**Table 5:** Suggestive associations: those significant under the Bonferroni correction for a single study but not for all 22 studies.

Phenotype	SNP	Chr	Position	Locus	Alleles	MAF	Score	BF	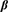	OR
Haircurl	rs1556547	6	10,378,363	OFCC1	A/G	0.40	7.81	5.61	−0.101	—
Eye color	rs10960751	9	12,665,264	TYRP1	C/T	0.37	7.54	5.35	0.240	—
Freckle	rs1042602	11	88,551,344	TYR	C/A	0.37	7.25	5.50	−0.356	—
Sneeze	rs11856995	15	94,126,647	NR2F2	T/C	0.30	7.13	5.71	−0.244	0.784

We have used a conservative threshold for reporting associations due to our increased multiple testing burden arising from the large number of traits analyzed; however, several SNPs score high enough that they would have been deemed significant as part of a single study. Shown are novel associations for haircurl and photic sneeze reflex plus replications for freckling and eye color.

### Phenotype data

The collection of a broad range of data from each participant allows us to control for some sources of bias and to assess the error rate of the phenotype collection.

To control for sources of bias, phenotypes were checked for correlations with a set of covariates (age, sex, and principal components of population variation). Covariates showing significant correlations (at the 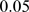 level) were included in the analysis (Supplementary Table 2 in Text S4).

In addition, by asking participants the same question multiple times in different ways, we were able to assess the repeatability of responses. We asked about eye color, hair color, freckles, handedness and age twice each in different places or ways. A total of 177 people were removed from analysis based on a single discordant answer to one of these questions. Overall, a total of only 0.72% of participants answered any pair of these questions inconsistently.

One source of bias unique to our replication studies was the fact that participants were shown their genetic data along with analyses of their data for approximately 100 traits and diseases. In some cases, this led to a severe bias. For example, a survey examining perceived performance in sprint versus long-distance races was placed on a web page within 23andme.com where customers were shown their genotype for rs1815739 (a SNP in *ACTN3* [14]). If they logged on before their genotype data had been processed, they saw the survey question alongside sample data. If their data was available, they were predicted to fall in a category including either world-class sprinters (carriers of the C allele) or endurance athletes (T homozygotes). The response distribution differed significantly (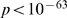) between respondents who had seen their genotypes with the suggested outcome versus those who hadn't. The results (Supplementary Table 1 in Text S5) of this comparison are consistent with large fractions (24.2% of C carriers, 41.2% of T homozygotes) of respondents answering differently than they would have if they had not seen their genotype data and interpretation.

Six of the 13 surveys were posted on pages where customers were shown their genotypes and predictions for related conditions. Due to the possibility of bias from this prediction, primary analysis of these six surveys considered only those participants who took the surveys before receiving their genetic data (so they only saw a sample prediction for the phenotype). As a result, none of these traits made our sample size cutoff.

For the 22 phenotypes considered here, participants were shown predictions for hair color, eye color, and freckling, although they were on separate pages from the surveys for these phenotypes. There was no evidence that for any of these traits participants who saw their genotypes gave different responses from those who did not (Methods). Therefore, we did not restrict attention to only those who hadn't seen their genotypes.

Survey response rates correlated with sex, age and the first (north-to-south) principal component of population structure. That is, women, people of northern European ancestry, and older people were more likely to answer more surveys than men, people of central European ancestry, and younger people (p-values 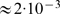, 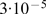, and 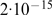), respectively. A genome-wide association study (GWAS) using the number of surveys answered as the trait analyzed did not show any significant associations (with p-values under 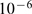) when these covariates were taken into account.

### Hair curl

We found regions associated with hair curl near the genes *TCHH*, *LCE3E* and *WNT10A*, as well as a region suggestively associated near *OFCC1*.

**Figure 5:**
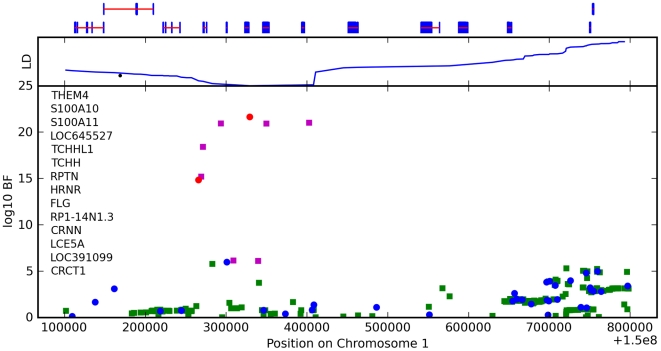
Bayes factors for genotyped and imputed SNPs for hair curl around *TCHH*. Plotted are 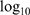 Bayes factors for all SNPs in HapMap in the region. Red/blue circles represent typed SNPs, magenta/green squares imputed SNPs; Red/magenta points have log Bayes factors over 6. Genes are marked in the top track with gene names inside the figure. Linkage disequilibrium (relative to the SNP with the largest Bayes factor) is plotted in the middle track.

We found an association between a SNP near *TCHH*, rs17646946 and hair curl, with score 41.8, see Figure 5 and Table 6. The minor allele is associated with straighter hair, with each A conferring a reduction in curliness of about 0.29 points on a scale from 0 to 5. There is evidence of a second, possibly independent, association in this region: the SNP rs499697, about 430kb away near *LCE3E*, has a score of 9.9. Here the minor, derived allele is associated with curlier hair, 0.13 points per G. These SNPs lie in the epidermal differentiation complex, which contains a large number of genes required for late epidermal differentiation. Many of these genes are involved in the production of the cornified envelope (CE), the highly cross-linked outermost layer of skin that provides mechanical protection from the environment, or are involved in cross-linking the CE with the network of keratin filaments in the cells.

**Table 6:** Counts for hair curl versus genotype at rs17646946.

	GG (%)	GA (%)	AA (%)
straight	923 (27.3)	797 (44.9)	111 (49.8)
slightly wavy	1629 (48.1)	743 (41.8)	96 (43.0)
wavy	568 (16.8)	174 (9.8)	10 (4.5)
big curls	187 (5.5)	42 (2.4)	5 (2.2)
small curls	61 (1.8)	17 (1.0)	1 (0.4)
very tight curls	16 (0.5)	3 (0.2)	0 (0.0)

The LD block containing the most significant SNPs includes four genes: *S100A11*, *TCHHL1* (*trichohyalin-like 1*), *TCHH* (*trichohyalin*), and *RPTN* (*repetin*). All four are putative calcium-binding proteins that contain two EF hand domains. *S100A11*, *TCHH*, and *RPTN* have all been shown to be associated with the CE [15]–[17]. Trichohyalin and repetin are both expressed at high levels in hair follicles—specialized epidermal structures that produce hair—specifically in the inner root sheath layer [17], [18].

**Figure 6:**
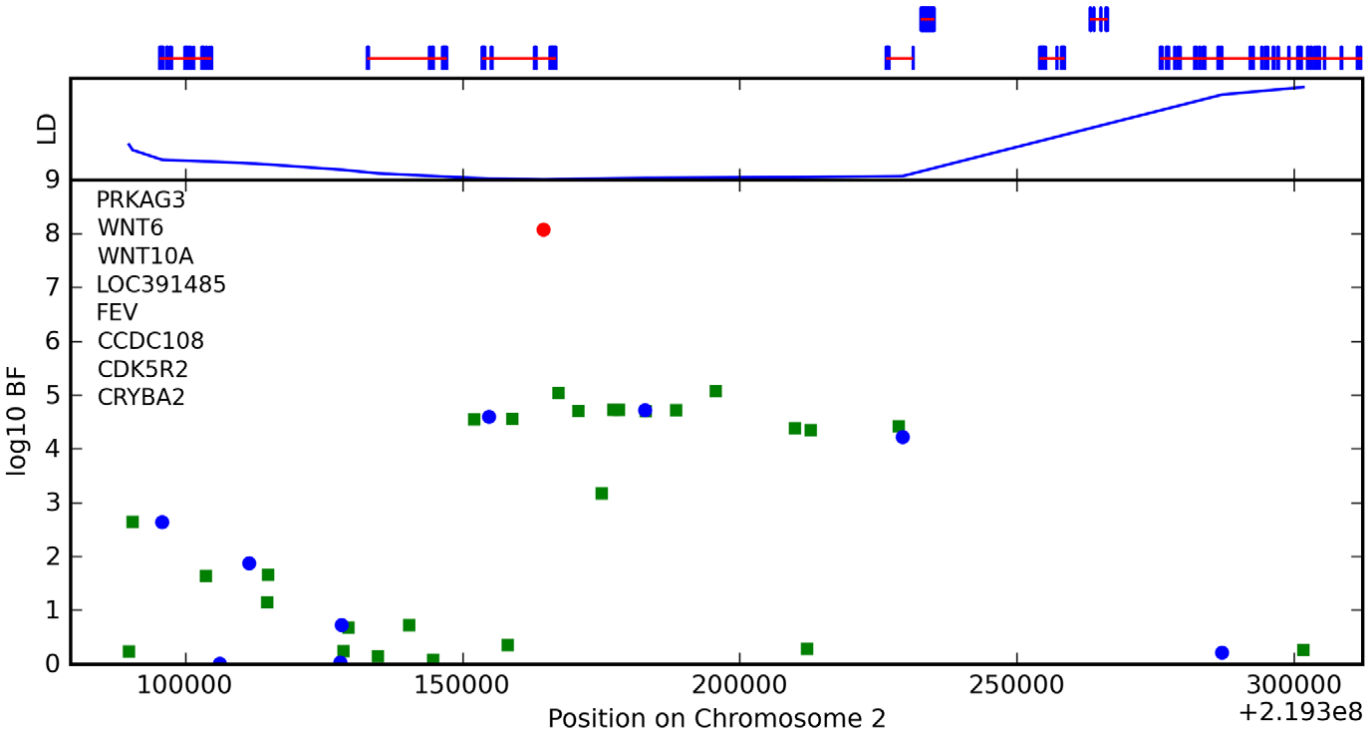
Bayes factors for genotyped and imputed SNPs for hair curl around *WNT10A*. For details, see Figure 5.

We also found an association between rs7349332 and hair curl, with score 13.4, in an intron of *WNT10A*. The minor allele is associated with curly hair, each T is associated with a 0.2 point change. See Figure 6 and Table 7.

**Table 7:** Counts for hair curl versus genotype at rs7349332.

	CC (%)	CT (%)	TT (%)
straight	1432 (36.1)	376 (28.3)	25 (26.3)
slightly wavy	1812 (45.7)	614 (46.2)	41 (43.2)
wavy	513 (12.9)	222 (16.7)	17 (17.9)
big curls	141 (3.6)	88 (6.6)	6 (6.3)
small curls	53 (1.3)	23 (1.7)	4 (4.2)
very tight curls	11 (0.3)	6 (0.5)	2 (2.1)

**Figure 7:**
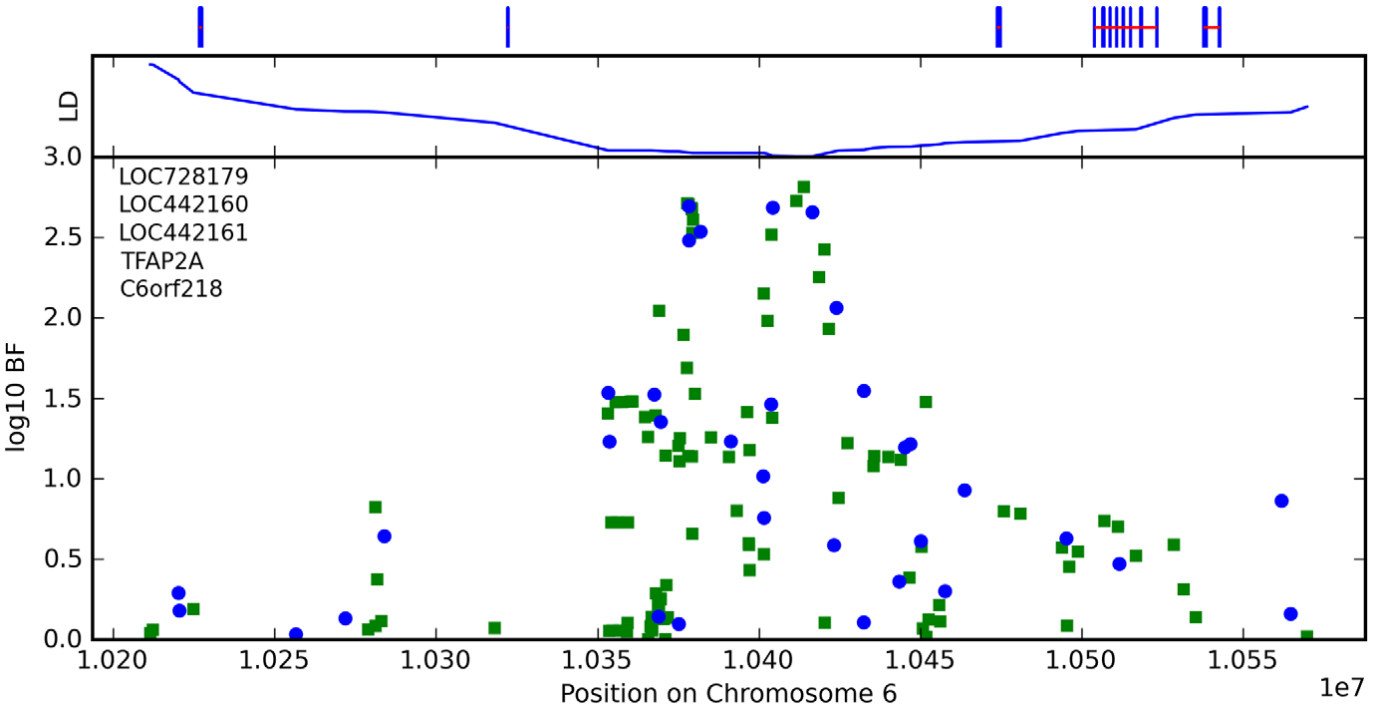
Bayes factors for genotyped and imputed SNPs for hair curl around *OFCC1*. For details, see Figure 5.

Finally, near *OFCC1* (*orofacial cleft candidate 1*), rs1556547 has a score of 7.8 and the minor allele is associated with straighter hair (0.1 points per G, Table 8) and Figure 7.

**Table 8:** Counts for hair curl versus genotype at rs1556547.

	AA (%)	AG (%)	GG (%)
straight	593 (30.6)	898 (34.6)	343 (40.2)
slightly wavy	901 (46.5)	1192 (45.9)	376 (44.0)
wavy	302 (15.6)	356 (13.7)	94 (11.0)
big curls	96 (5.0)	105 (4.0)	34 (4.0)
small curls	35 (1.8)	40 (1.5)	5 (0.6)
very tight curls	9 (0.5)	8 (0.3)	2 (0.2)

### Asparagus anosmia

Odorous urine after eating asparagus is thought to be due to the excretion of methanethiol, a volatile sulfur-containing compound that smells like rotten or boiling cabbage [19]. In mammals, odor detection is mediated through olfactory receptors (ORs), seven-transmembrane domain proteins that are expressed in the cell membranes of olfactory neurons and are responsible for detection of odorants. A number of ORs are known to have specific odor sensitivities, and account for some of the variation in an individual's ability to detect those odors [20].

**Figure 8:**
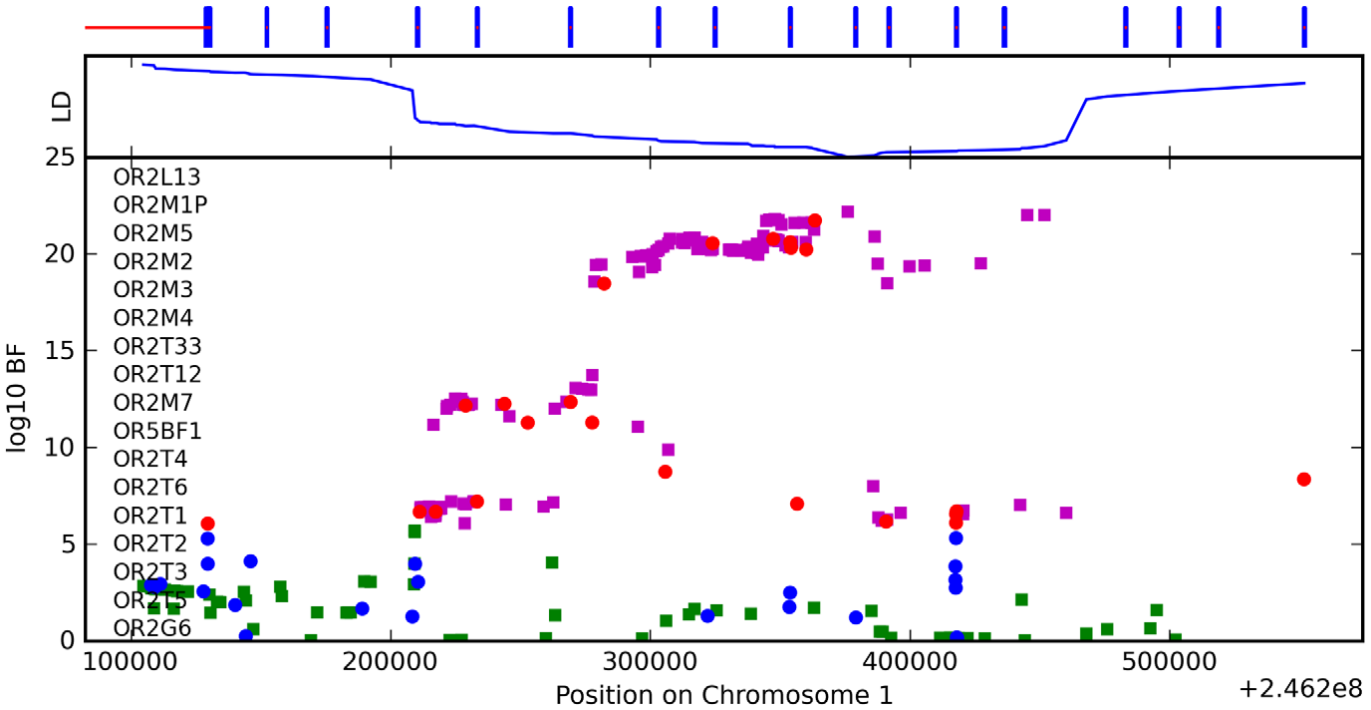
Bayes factors for genotyped and imputed SNPs for asparagus anosmia around *OR2M7*. For details, see Figure 5.

We have found a region on chromosome 1 (Figure 8) containing a cluster of olfactory receptor genes that is significantly associated with the ability to smell asparagus metabolites. This region contains 39 OR genes (ten annotated as pseudogenes). The LD block that contains the most significant SNP found in this study rs4481887 (with a score of 23.21) includes ten genes, all olfactory receptors: *OR2M2*, *OR2M3*, *OR2M4*, *OR2T33*, *OR2T12*, *OR2M7*, *OR5BF1*, *OR2T4*, *OR2T6*, and *OR2T1*. Two of these (*OR2M3*, *OR2T6*) are annotated as pseudogenes in ORDB [21]. The most significant association, at rs4481887 (about 9kb upstream from *OR2M7*), has score 23.2 and appears to be acting in a dominant fashion decreasing the odds of anosmia, with an odds ratio under a dominant model of 0.48 (the odds ratio of 0.60 reported in Table 2 is under an additive model). See Table 9 for details.

**Table 9:** Counts for asparagus anosmia versus genotype at rs4481887.

	GG (%)	GA (%)	AA (%)
Can smell	1458 (56.7)	1313 (70.9)	231 (74.0)
Anosmic	1114 (43.3)	540 (29.1)	81 (26.0)

Another nearby SNP, rs7555310, with a similar odds ratio but slightly weaker score of 21.7, is a non-synonymous change in *OR2M7*. This SNP changes a valine to an alanine and is predicted to lie in one of the transmembrane domains of this protein. Though the valine is well conserved in mammals, the functional significance of this conservative amino acid change is not clear. Due to the extensive linkage disequilibrium in this region, it is impossible to tell without additional evidence which gene most likely codes for the receptor that detects this odorant.

### Photic sneeze reflex

For photic sneeze reflex, we find a novel association with rs10427255 (score 10.9 and an OR of 1.32). This SNP lies in a large intergenic region of 2q22.3 between *ZEB2* and (the annotated pseudogene) *PABPCP2* (725kb and 1.2MB away, respectively).

**Figure 9:**
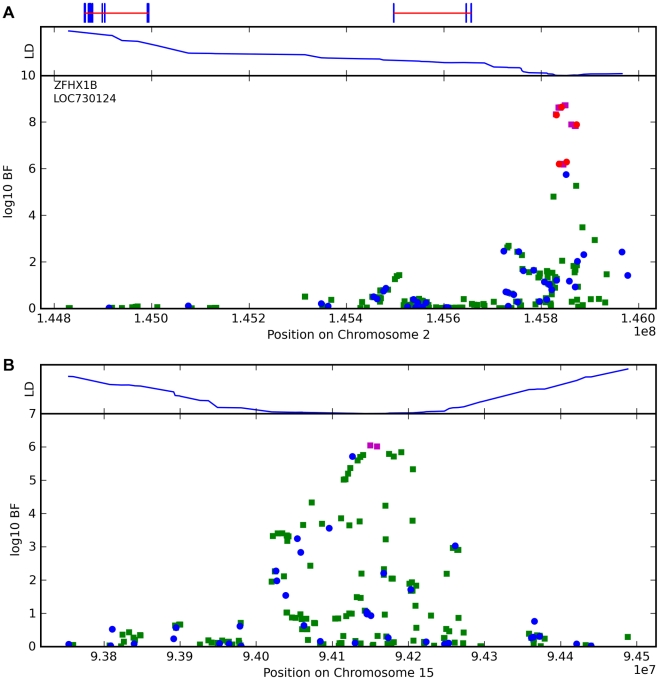
Bayes factors for genotyped and imputed SNPs for two photic sneeze associations. Regions shown are (A) near *ZFHX1B/ZEB2* on 2q22.3 and (B) near *NR2F2* on 15q26.2. For details, see Figure 5.

We also find a suggestive association with rs11856995 (score 7.13 and OR of 0.78). It also lies in a large intergenic region of 15q26.2, with the nearest gene being *NR2F2*, some 560kb away. Details for the SNPs in these regions are shown in Figure 9, Table 10, and Table 11.

**Table 10:** Counts for photic sneeze reflex versus genotype at rs10427255.

	TT (%)	TC (%)	CC (%)
No sneeze	1168 (72.4)	1795 (67.9)	677 (59.9)
Sneeze	446 (27.6)	849 (32.1)	453 (40.1)

**Table 11:** Counts for photic sneeze reflex versus genotype at rs11856995.

	TT (%)	TC (%)	CC (%)
No sneeze	1714 (65.2)	1530 (67.8)	397 (78.8)
Sneeze	916 (34.8)	727 (32.2)	107 (21.2)

### Freckling

We find one new association for freckling and replicate two known regions. The novel association is at rs2153271, in an intron of *BNC2* (Zinc finger protein basonuclin-2), with a score of 9.4 and an estimated 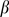 of −0.4 (on a 17 point scale). See Supplementary Table 2 in Text S6 for details.

**Figure 10:**
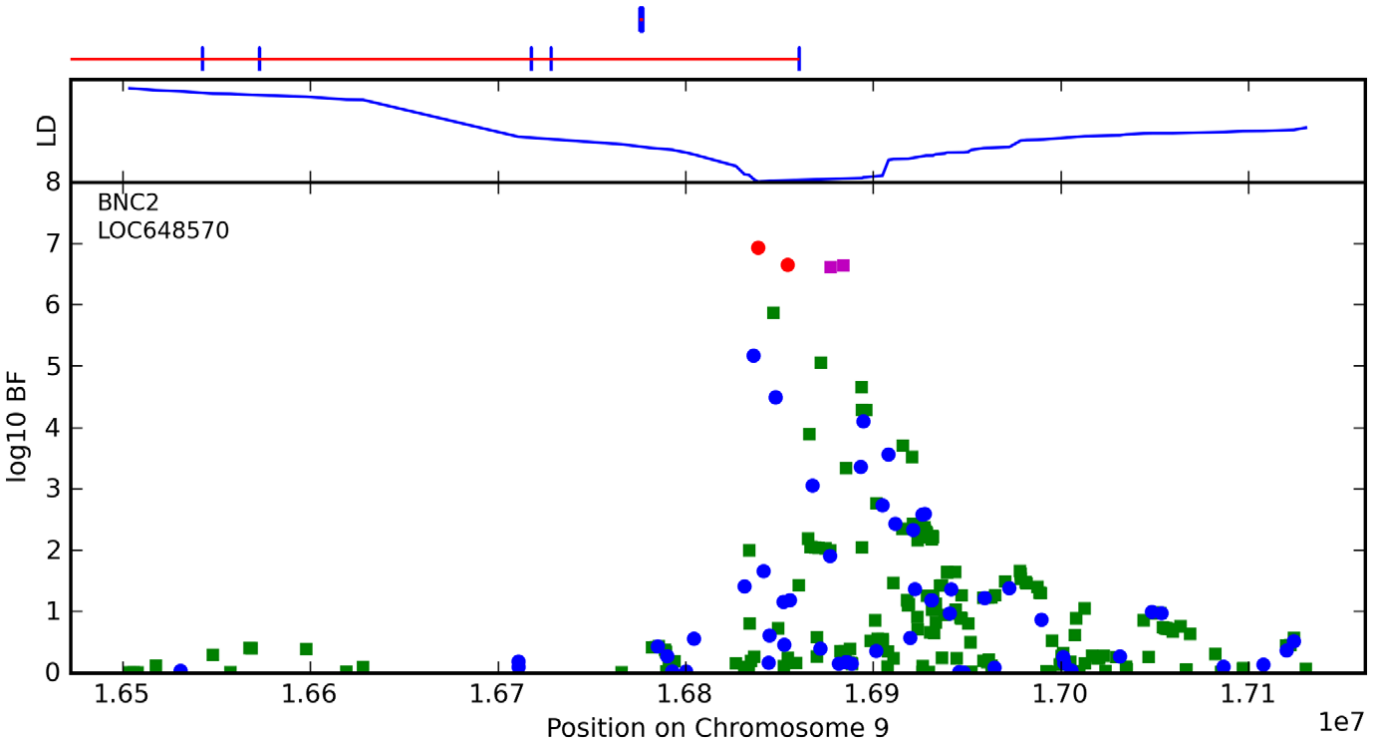
Bayes factors for genotyped and imputed SNPs for freckling around *BNC2*. For details, see Figure 5.

Our most significant association, rs12203592, with score 90.7, lies in an intron in *IRF4* (Figure 10). This SNP was previously associated with hair color, eye color, and tanning response to sunlight [5]. A more mildly associated SNP, rs1540771 (with score 13.2), in this region has previously been associated with freckling (as well as eye color, sensitivity to sun, and hair color) [4], however rs12203592 (60kb away) was not typed in that analysis. For eye and hair color and tanning ability it was suggested [5] that in fact rs12203592 was in closer LD with the causal SNP. Here we confirm this finding for hair and eye color and establish the same for freckling.

The other loci we associate with freckling are *MC1R*, *ASIP*, and *TYR*, all known associations [2], [22]. Although the SNPs selected by the regression procedure as most influential are slightly different than those for red hair, the sets are quite similar for these highly correlated phenotypes.

### Hair color

We confirm known associations for hair color, both blond to brown and non-red to red. For blond to brown, excluding red, we find hits in five regions: *OCA2/HERC2*, *IRF4*, *SLC24A4*, *SLC45A2*, and *MC1R* (aside from *MC1R*, the same set of regions as [5] in their analysis excluding red hair). A multiple regression using the seven SNPs in Table 3 (with sex and five principal components) estimates that these five regions together explain about 28.1% of the variance in hair color (blond to brown) within northern Europe.

In the *OCA2/HERC2* region, rs12913832, first found by [3], has a score of 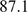 and 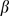 of 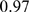, explaining 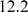
% of the variance. These numbers (as well as those for the other SNPs) concord well with those in [5] (which estimated 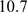
% of the variance was explained by this SNP and 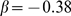 using a five point scale from dark to light as compared to our eight point scale from light to dark). For *IRF4*, rs12203592 has an estimated 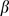 of 0.59 and explains 3.9% of the variance. For *SLC24A4*, rs12896399 has an estimated 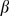 of 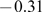 and explains 1.7% of the variance. In *SLC45A2*, rs16891982 has 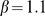 and explains 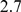
% of the variance. Finally, rs12931267 near *MC1R* has 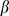 of 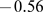 and explains 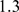
% of the variance.

Sulem et al. [4] found associations for hair color in four of these five regions (excluding *SLC45A2*) as well as *KITLG*. The SNP rs12821256 in *KITLG* showed a mild but significant association with hair color in our study, with a score of 4.2 and 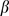 of 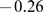 (95% CI from 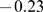 to 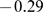). This is similar to the relatively weak effect for this SNP found in [5].

For the other direction of variation in hair color (red versus non-red hair) we found many associated SNPs in the *MC1R* region, long known to be associated with this phenotype [2]. Although some of the SNPs contributing to the model in this region lie far from MC1R, some of the biggest effects are from rs1805008 and rs1805009, non-synonymous changes in the *MC1R* gene. This region is strongly associated with common variation in red hair [4], [5]. We also replicated the claim that a large haplotype containing the pigmentation gene *ASIP* is associated with red hair (also associated with burning and freckling in [22]). While rs291671 is about 900kb away from ASIP, it appears to be tagging the same haplotype found there.

### Eye color

We confirm known associations for each of two traits: blue to brown and green versus blue eye color (Table 4). For blue to brown, we confirm six regions: *OCA2/HERC2*, *SLC24A4*, *IRF4*, *SLC45A2*, *TYR*, and *TYRP1*. In *HERC2*, the well-known SNP rs12913832 has a score over 300 and 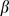 of 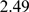 (thus the A allele is associated with darker eyes). This single SNP explains 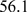
% of the variance in eye color. In *SLC24A4*, rs12896399 has a score of 15.9 and 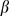 of 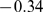, explaining 1.6% of the variance in eye color. In *IRF4*, rs12203592 has a score of 14.7 and 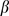 of 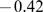, explaining 1.4% of the variance. In *SLC45A2*, rs16891982 has a score of 11.8, 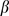 of 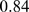, and explains 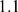
% of the variance. In *TYR*, rs1393350 has a score of 8.5, 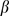 of 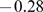, and explains 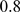
% of the variance. Finally, as shown in Table 5, (as it does not quite meet our strict genome-wide significance levels), rs10960751 in *TYRP1* has a score of 7.5, 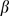 of 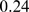, and explains 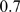
% of the variance.

For green versus blue eyes, three regions show associations: *OCA2/HERC2*, *SLC24A4*, and *TYR*. For this trait, SNP rs12913832 in *OCA2/HERC2* has a score of 51.5 and an estimated allelic OR of 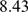. The SNP rs1667394 in this same region has an estimated OR of 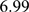 (4.85–10.06), very close to the ORs in [4], which range from 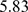 to 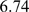 in their three populations. In *SLC24A4*, rs12896399 has a score of 22.8 and OR of 0.58 (0.52–0.65), again similar to [4], where the ORs range from 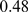 to 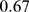. In *TYR*, we report rs1847134 with a score of 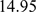. The nearby SNP rs1393350, which was reported to have ORs between 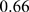 and 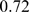 in Sulem et al. has an OR of 0.64 (0.57–0.72) in our data.

## Discussion

We have conducted genome-wide association studies for 22 traits. The sheer size of this study was possible due to an original, web-based, parallel design. We have found novel associations for hair curl, freckling, photic sneeze reflex, and the ability to smell the urinary metabolites of asparagus. In addition, we have replicated a wide array of associations for pigmentation traits; these replications show great consistency with the numbers reported in other studies.

### Associations

#### Hair curl

We have found associations between hair curl and SNPs near the genes *TCHH*, *LCE3E*, *WNT10A*, and *OFCC1*. that together explain about 6% of the variance in hair curl within northern Europe. The association with *TCHH* replicates the finding of [23] (which also mentions *WNT10A* as a non-significant but interesting association).

In the *TCHH* region, the strongly associated imputed SNP rs11803731 (in an exon of *TCHH*, causing the change M790L) is of note. This position is a possible helix initiator (between two prolines and a probable helix [24]), and differences in the ability to initiate the helix due to mutations at this position could lead to changes in protein behavior.

Experiments with *in vitro* cultures of curly and straight human hairs have shown that hair shape appears to be programmed from the basal area of the follicle, which includes the inner root sheath. Curly hairs originate from follicles that have a golf-club like bend at the base, while straight hairs originate from straight follicles [25]. Asymmetry in the thickness of the inner root sheath, which surrounds and provides protection for the growing hair at the base of the follicle, may play a role in moulding the shape of the hair [26]. Wool follicles from sheep also have a curved bulb, and exhibit an asymmetry in the thickness of trichohyalin containing structures, with more trichohyalin on the concave side [27]. Biochemical studies of the trichohyalin protein suggest that trichohyalin provides mechanical strength to the hair follicle by cross-linking the CE with the cytoplasmic keratin filament network in inner root sheath cells [28].

The bulk of this evidence points to *TCHH* as the most likely candidate for controlling hair curl in this region. However, due to the related nature of these genes, it is difficult to rule out *S100A11*, *TCHHL1*, or *RPTN* as playing a role. A second association 400kb away appears to be independent, but again there are many genes nearby (*LCE3E*, *LCE5A*, etc.) that could play a role.

For *WNT10A*, there appears to be a direct connection to hair morphology: mutations in this gene are known to cause odonto-onycho-dermal dysplasia, which includes dry, misformed hair as a symptom [29]. Also, WNT10A is upregulated in hair follicles at the beginning of a new growth cycle [30].

Finally, a translocation breakpoint in *OFCC1* has been associated with orofacial clefting [31]. Orofacial clefting and ectodermal dysplasia can appear together, for example in EEC, Rapp-Hodgkin, and Hay-Wells syndromes [32].

#### Asparagus anosmia

It has been debated whether variance in the production or the detection of methanethiol explains the differences in the reporting of the ability to detect asparagus metabolites in urine. One study suggested that production is an autosomal dominant trait [10]; a second study concluded that the variation is instead in the ability to detect the compound and that it is a roughly bimodal trait with respect to the dilution at which the odor is detected [11].

We have identified a locus associated with this trait. This locus lies within a region containing many olfactory receptors and appears to act in a dominant fashion. Both of these facts suggest that the genetic variation in this trait is in the ability to detect the odorant.

#### Freckling

We have found a novel association between freckling and rs2153271 in an intron of *BNC2*. *BNC2* is a potential transcriptional regulator in keratinocytes based on its close similarity to *BNC1* (*basonuclin 1*) [33] (which is involved in keratinocyte proliferation), however there is evidence that *BNC1* and *BNC2* have different functions [34]. As the pigment that shows up in freckles is housed in keratinocytes, there could well be a link between *BNC2* and freckling.

We have also found a strong association between rs12203592 in an intron of *IRF4* and freckling. This region has previously been associated with freckling, however this SNP appears to more closely tag the causal variant than the previous association in this region [4]. Like many other genes associated with pigmentation, *IRF4* appears to be regulated by *MITF* [35]. Also, there is a striking, sudden drop-off in significance around this SNP that does not appear to be due to the presence of any recombination hotspots nearby. There is evidence of substantial copy-number variation (CNV) in this region (cf. [36], [37]). Although we are unable to detect CNVs here, it could explain the LD pattern.

#### Hair color

We replicated many known associations for hair color, both blond to black and red versus non-red. Of interest is the fact that rs12821256 in *KITLG*, discovered in [4], does not have a large effect size either here or in [5]. This raises the question of whether the OR of 1.9 reported in [4] is an overestimate or whether the discordant results are due to differences in the populations studied (as Han et al. studied a U.S. population probably similar to our cohort while Sulem et al. studied Icelandic and Dutch populations).

#### Photic sneeze

There is evidence that photic sneeze reflex is genetic: Peroutka and Peroutka [38] concluded that this trait is inherited in an autosomal dominant fashion. We find one region associated with photic sneeze reflex about 725kb away from *ZEB2* and a second suggestively associated about 550kb away from *NR2F2*.

*ZEB2* is mutated in individuals with Mowat-Wilson syndrome, which has seizures as a common symptom. There may be a link between photosensitive epileptic seizures and photic sneeze reflex (triggered by a sudden switch from being dark-adjusted to light) [39], providing a possible link between this region and photic sneeze reflex.

*NR2F2* (also known as *COUP-TFII*), also has a possible link to sneezing: it interacts with *NR2F6* [40] and *NR2F6* knockout mice show defects in the locus ceruleus (part of the brainstem) [41]. Certain stimulations cause signals to be sent to the brainstem to cause a sneeze; it is thought that photic sneeze reflex also progresses through this fashion [42], [43]. Also, the locus ceruleus is disrupted in Rett syndrome, which also has seizures as a common symptom [44], [45].

#### Handedness

Handedness has putative genetic associations [12] (an association found only in dyslexic siblings). The haplotype there associated with left-handedness (when paternally inherited) was at most moderately associated with left-handedness in our data (estimated OR of 1.17, 95% CI of 0.96–1.42, p-value of 0.06). However, we are underpowered to detect such a small OR in this fairly rare (8% frequency) haplotype.

Looking at the laterality measures (handedness, footedness, ocular-dominance, and hand-clasp) as a whole, there is no overlap between the marginal hits for any of these phenotypes: no SNP has a score of above 5 for more than a single one of these measures.

### Research framework

In this initial set of results, we have shown parallel, web-based phenotype collection to be quick and reliable. The population structure present in the wider dataset makes statistical analysis more involved than in a typical GWAS, however it does not influence the results. Furthermore, this complex population structure facilitates studies of multiple ethnicities simultaneously. It also compels the development of robust methods for genetic research in populations reflecting the complexities of human populations.

This design has several statistical advantages over traditional studies. By centralizing many studies, we have the ability to avoid publication bias by reporting statistics on a large collection of independent association studies, including both positive and negative results. Such a bias is a concern [46]. For example, imagine that 20 separate groups each perform a GWAS on a phenotype with no true association. One of them will probably find a result by chance, and this will be the only result submitted and published. While false positives are unavoidable in a statistical test, we can reduce them by incorporating the total number of simultaneous trials into the multiple testing calculation. We thus formalize the notion of a significance and testing burden across multiple scans, rather than with respect to a single GWAS.

Ignoring linkage disequilibrium (i.e., if one assumes the approximately 10 million tests performed in this paper are all independent), one would expect to see one or two suggestive associations (with scores between 7.1 and 8.4) by chance in about half of all studies similar to ours. This is a conservative estimate; the false-discovery rate (FDR) analysis estimates a FDR of about 5.2% for a cutoff of 7.1, meaning that we would expect 5.2% of the associations with scores in this range to be false positives.

In addition, genotypes for cases and controls are collected and treated the same way, avoiding sources of bias (cf. [47]). In fact, an individual will typically be a case for several studies and a control for several others. This is an extension of the model used in [48], where controls were shared across seven studies.

**Figure 11:**
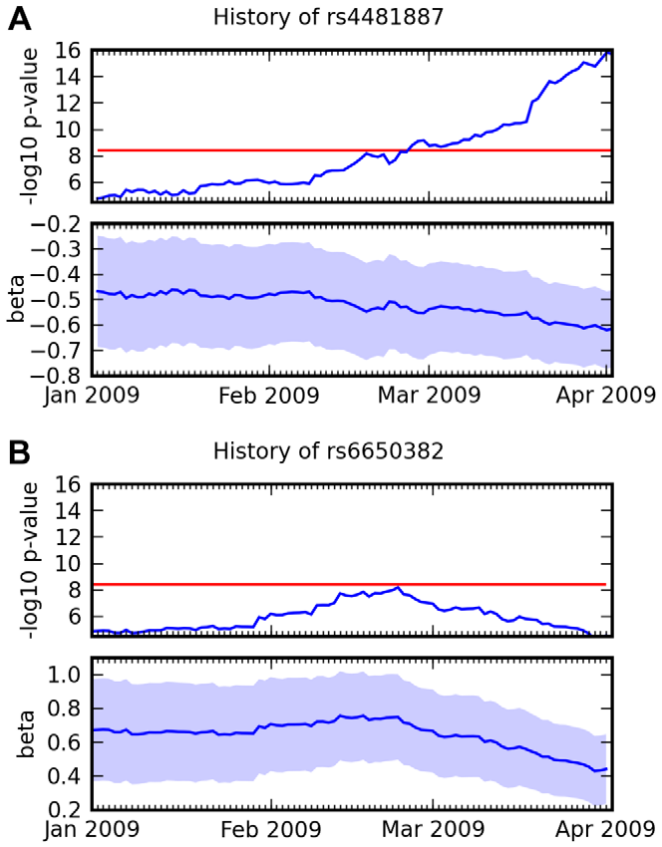
Association over time for two SNPs and two traits. (A) Association between rs4481887 and asparagus anosmia, steadily increasing in certainty. (B) Association between rs6650382 and photic sneeze: a very promising initial result, but 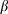, the log odds ratio, regressed towards 0. Both traits were assessed in the same survey, so they had approximately the same number of responses at all points in time. Scores (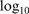 p-values) and regression coefficients are plotted using the genotype and phenotype data that was available at various points in time. The red line indicates our significance threshold of 8.4.

This new research model raises interesting methodological questions. For example, since new participants join the study continuously, results are constantly changing. For this study, we chose to set an end date and inclusion criteria in advance to avoid possible bias due to choosing a stop date according to the latest results. However, it is an interesting problem to design a criteria for significance using a continually expanding cohort to replace the traditional design of phased data collection with discovery followed by replication. For example, Figure 11 shows the history of two SNPs from this study that both had scores over 8.4 at one point in time. Further data shows that one appears to have been a true positive and one a false positive.

While we have not formalized this notion in this paper, we note that the novel associations we report here have been steadily increasing in significance (cf. Figure 11A and Supplementary Figure 7 in Text S6) and that they have several other significant SNPs in the same LD block. These facts are strong evidence that these novel associations are not the result of some genotyping artifact.

Our research model makes possible studies that might be infeasible otherwise due to the low marginal cost of asking additional questions over the web and the speed of broadcasting recruitment messages in parallel online. This speed and flexibility allows us to easily study traits for which funding may not be readily available, such as asparagus anosmia.

We believe that providing participants with well-explained descriptions of their genetic data can substantially benefit genetic research as a whole. It is an opportunity to harness the public interest in genetics and make the participants a part of the study. This participation provides a wonderful chance to educate the public about genetics, statistics and research and to give something back to the individuals who contribute to genetic research.

## Methods

### Cohort

Participants were drawn from the customer base of 23andMe. After purchase, customers were shipped a kit for saliva collection, containing a bar-coded tube to be returned and a code to claim that kit online. They entered this code on the 23andme.com website, created an account, and agreed to a Consent and Legal Agreement. Upon signing up for 23andWe (the portion of the website that allows participants to enter phenotype data through a series of surveys), participants were reminded that they had consented to the use of survey responses for research.

The Consent and Legal Agreement stated that participants' genotype data and whatever phenotype data they entered would be used for internal research after being coded and stripped of individually identifying information (“anonymized”). Individually identifying information refers to personal information that is collected during purchase, such as name, credit card information, billing and shipping addresses, and contact information such as an email address or telephone number. The consent form stated that aggregate genetic data might be shared publicly (as it is in this paper). However, it provided that individual-level genetic data would not be shared with outside researchers without separate consent. For this study, all access to individual-level data occurred at 23andMe by full-time 23andMe employees. One author (IP) was working as a 23andMe consultant and did not access individual-level data. Another author with a secondary, non-23andMe affiliation (JM) was acting in her capacity as a 23andMe employee during her work on this study.

As part of the consent form, participants were told they could withdraw from all future research at any time by emailing 23andMe. This information was also present as a FAQ on the 23andMe website. A small number of customers have elected this option and those who did so before January 30, 2010 were not included in any analysis in this paper.

All anonymized data was placed in a secure research environment accessible only by 23andMe scientists. These scientists had no access to individually identifying information that was held separately at the company. The scientists also had no interaction with study participants.

To obtain formal recognition of this strict partitioning of the research, following consultation with the editors at PLoS, we sought and were granted an independent determination by a commercial Institutional Review Board (IRB) that this research did not involve human subjects under the Department of Health and Human Services definition, 45 CFR 46.102(f). This definition states that research performed on anonymized data with no contact between investigators and participants does not constitute research on human subjects. It is 23andMe policy that every scientist who obtains access to the restricted research environment first undergo online human subject assurance training made available by the Office for Human Research Protections (OHRP).

23andMe is committed to an ongoing evaluation of our process of Institutional Review and oversight of Consent as part of our commitment to protection of participant rights. There are significant novel challenges in this process relating to the evolution of policies concerning privacy of genetic data, the ongoing nature of our study, and the return of data to participants. Our study participants arguably have greater opportunity to review and consent to their involvement than most participants in genetic studies. However, they also have greater access to their own genetic data and hence to personal results that may impact their self-perception. We expect the definition of human subjects research to evolve along with standards for protecting individual identity and providing public access to GWAS results. We are continually evaluating our protocols with an external, AAHRPP accredited IRB in an effort to set the standard for web-based genetic studies.

### Genotyping and SNP quality control

DNA extraction and genotyping were performed on saliva samples by National Genetics Institute (NGI), a CLIA licensed clinical laboratory and a subsidiary of Laboratory Corporation of America. Samples were genotyped on the Illumina HumanHap550+ BeadChip platform, which included SNPs from the standard HumanHap550 panel in addition to a custom set of about 25,000 SNPs selected by the 23andMe staff. Every sample that failed to reach 98.5% call rate was re-analyzed. Individuals whose analyses failed repeatedly were re-contacted by 23andMe customer service to provide additional samples, as is done for all 23andMe customers.

Two slightly different versions of the genotyping platform were used in this study. See Text S3 for details about the two versions and quality control measures used to eliminate a subset of poorly performing assays.

SNPs with a call rate under 98% were excluded from analysis, as were those with minor allele frequency under 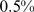 or a p-value for Hardy-Weinberg equilibrium (using the test from [49] within all the northern Europeans in the database) under 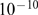. Both minor allele frequency and Hardy-Weinberg statistics were calculated within our dataset. Due to the two slightly different platforms in the analysis the no-call rate was calculated only among individuals genotyped on a given platform. However, all SNPs discussed in this paper are contained in the intersection of the two platforms. In addition, a total of 1553 SNPs with Mendelian discordance rates (the fraction of trios within the entire 23andMe customer database in which the called SNPs followed an impossible inheritance pattern) of at least 1% were discarded. In the end, 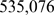 SNPs were used with an average call rate per person on these SNPs of 99.91% and a median call rate of 99.97%.

### Statistical analysis

All p-values were calculated using linear or logistic regressions as appropriate (or the corresponding score tests in the case of analyses without covariates). For linear regressions with covariates, we used a simple multilevel model: first we performed the regression of the phenotype solely on the covariates and then regressed the residuals against individual SNPs. This multilevel model is similar to the model in EIGENSTRAT [50]. In addition to being faster this multilevel model avoids multicollinearity. Regressions were performed using R [51], PLINK [52], [53], or internal software packages. The estimated regression coefficient for each SNP, denoted by 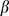 throughout, refers to a coding of genotypes as 0, 1, 2 counting the number of minor alleles present. The strand used was determined from NCBI build 36 of the human genome. Codings for the phenotypes discussed are given below and in Text S5.

Bayes factors (shown in region plots and in Text S6) were calculated using the default prior in BIMBAM [54], [55]. Regions of interest were further analyzed by imputation against the phased HapMap CEU subset [56] using BIMBAM.

Manhattan and quantile-quantile plots were trimmed at a p-value of 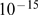 in order to better show the details and also since for extremely small p-values the standard approximations become less exact.

For the tables (e.g., Table 2), the set of significant SNPs was pared down within each region using a backwards stepwise regression procedure that attempted to minimize the AIC (Akaike's information criterion, using the step command in R). As input to this procedure, all SNPs within the region with p-values under 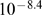 were included and only those that contributed to the optimal model were displayed. Of particular note is that the displayed SNPs were not necessarily all significant upon correction for the other SNPs in that region, only that the AIC judged that a SNP usefully contributed to the joint model. See Table S1 for all SNPs in these studies with scores above 6.0.

### Multiple testing

We give two estimates for the multiple testing burden over all analyses. Most conservatively, a Bonferroni correction that takes into account the number of SNPs tested and the number of phenotypes tested yields a score of 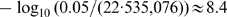 corresponding to a significance level of 0.05 for all 22 studies simultaneously.

In order to estimate the chance that the suggestive associations are true positives, we calculated the false-discovery rate (FDR) [57] over the set of p-values for all 22 studies. For each study we excluded all SNPs within 100kb of a SNP with score over 8.4 from the FDR analysis (removing the “true positives” and most SNPs in LD with them from the analysis). The analysis concluded that a cutoff of 7.1 corresponds to an estimated FDR of 5.2% (meaning that 5.2% of the SNPs with scores between 7.1 and 8.4 would be expected to be false positives). A cutoff of 6.5 raises the FDR to 10.4% and a cutoff of 6.0 raises the FDR to 19.8%.

### Analysis of related individuals

We measured identity by descent (IBD) for all pairs of participants using a novel algorithm that acts on unphased data by comparing homozygous calls in a window (Text S2). A set of “unrelated” participants was defined by requiring that no two individuals share over 700 cM IBD, counting both full (diploid) and half (haploid) levels of identity by descent. This level of relatedness (approximately 20% of the genome) corresponds approximately to the minimal expected sharing between first-cousins in an outbred population (Supplementary Figure 1 of Text S2).

### Population stratification

Extensive population structure exists in the customer base as a whole, which includes individuals from around the world. This structure might yield spurious associations if not taken into account [50]. We selected a subset of individuals having northern European ancestry (including western Europe as well) using multi-dimensional scaling (MDS) and a support vector machine (SVM) trained on three datasets containing individuals of known ancestry. Two of these datasets were the 1043 HGDP-CEPH individuals [58] and 326 individuals of European ancestry from Illumina's iControlDB and Peter Gregersen. The third dataset consisted of several hundred customers who reported having four grandparents either of Ashkenazi descent or having lived in a single European country for several countries chosen to complement the existing datasets. See Text S1 for details.

Only unrelated (in the aforementioned sense) individuals from this subset were considered during the present association studies. Although the sample included only individuals of northern European ancestry, mild population structure still existed. Inspection of the eigenvalues showed that the first five principal components captured the majority of this structure (Supplementary Figure 2 of Text S1). Thus, to guard against spurious associations we included each individual's first five principal components as covariates in the regression model for those traits that showed association with the principal components. For each phenotype, we also computed genomic control inflation factors (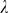) [59]. They were quite close to unity for the adjusted models (Table 1). See Text S1 for details.

### Phenotypes

Phenotype data was collected via 13 surveys administered to research participants via the 23andMe.com web site. See Figure 1 and Text S5 for further details.

#### Inclusion criteria

Due to the frequent release of new questions and the continual accumulation of responses, criteria for inclusion of genotype and phenotype data were established before the writing of this paper. We analyzed all phenotypes derived from the 13 surveys released between May and October of 2008 that met our criteria. Only responses and genotypes obtained before January 30, 2010 were used.

Our requirements for selection of phenotypes were as follows:

1. At least 1500 responses among unrelated northern Europeans.
2. For binary phenotypes, at least 500 cases.
3. For surveys that were displayed alongside genetic predictions, only respondents who answered before their data was ready were included.
4. For phenotypes with significant correlations with covariates, only respondents who had provided those covariates were included.

Twenty-two traits met these criteria, they came from the six surveys “Ten Things About You,” “Ocular Dominance,” “Handedness,” “Optimism,” “Ten More Things About You,” “Footedness” and “Pigmentation.” The phenotypes analyzed in the paper are described in detail below, see Text S5 for details on the others.

For the analysis of whether seeing genotypes influenced survey responses, we looked at SNPs that were reported to the customer as influencing five traits analyzed here: rs12896399 and rs1393350 (green eye color), rs12913832 (eye color), rs1805007 (red hair), rs1667394 (hair color), rs4778138 and rs1805007 (freckling). For each SNP, we tested whether seeing their data influenced their responses, controlling for their genotype at that SNP, sex, age, and five principal components. Unadjusted p-values (using a logistic regression) were all over 0.1 except for rs1667394 and hair color, which had a p-value of 0.02. After adjusting for the seven tests performed, we fail to reject the hypothesis that seeing the data did not influence people's responses.

#### Hair curl

Participants were asked “Is your hair naturally straight or curly?” Answer choices were presented as a series of six pictures with accompanying descriptive text. Analysis used the codings (0 = “stick straight,” 1 = “Slightly wavy,” 2 = “Wavy,” 3 = “Big curls,” 4 = “Small curls,” 5 = “Very tight curls”) in a linear regression. The pictures are shown in Supplementary Figure 1 of Text S5.

#### Ability to smell the urinary metabolites of asparagus

The question asked was “Have you ever noticed a peculiar odor when you pee after eating asparagus?” This phenotype was scored as cases and controls where those who could not smell the odor after eating asparagus were considered cases. Participants who answered that they did not know or did not eat asparagus were excluded.

#### Freckling

Participants were asked to compare the amount of freckling on their face, arms, and shoulders to three series of images (Supplementary Figure 2 of Text S5). There were six images in the “arms” and “shoulders” categories and seven images in the “face” category. Each category was scored from zero to five or six and then the three categories were summed, leading to a score between zero and sixteen ranging from not-freckled to heavily freckled.

#### Hair color

We performed two analyses of hair color: blond to brown and red versus not red. For blond to brown, we regressed an ordinal coding (0 = blond, 1 = dark blond, 2 = light brown, 3 = reddish brown, 4 = medium brown, 5 = dark brown, 6 = black) for hair color on the ordinal coding for genotype (0, 1, 2) as well as five principal components and sex.

For red versus non-red hair color, participants were asked to “describe the amount of red in my hair (before I went gray, if I am gray now)” with available choices “No red at all,” “A tinge of red,” “Some red” and “A lot of red” which were coded from 0 to 3 in a linear regression.

#### Eye color

We performed two analyses of eye color: blue to brown and blue versus green. Eye color was assessed by asking participants to match their eye color to a set of 7 pictures (without accompanying text) ranging from blue to dark brown. These were coded as 0 = blue through 6 = dark brown for the main analysis. See Supplementary Figure 4 of Text S5 for the pictures.

For blue versus green eye color, blue eyes were treated as controls and greenish-blue or green eyes were treated as cases.

#### Photic sneeze reflex

Participants were asked one question for this trait: “Do you have a tendency to sneeze when exposed to bright sunlight?” Available answers were “Yes” and “No, what are you talking about?” People who did sneeze were treated as cases, those who did not were controls.

#### Sweet taste preference

Participants were asked “When you're in the mood for a snack, what kind of snack do you usually reach for?” Available answers were “Sweet” “Salty or savory” “Both” or “Neither.” People answering either both or neither were disregarded. People reaching for sweet snacks were treated as cases.

#### Handedness

Handedness was scored on an eight-point scale (where 1 was “pure right” and 8 was “pure left”) using the questions and scoring from [60]. The haplotype analysis used Beagle [61] to phase data on chromosome 2 between positions 80375000 and 80523000. To calculate an odds ratio, we collapsed the eight-point scale to a binary scale, using people scored as pure right-handed or right-handed with weak left-handed tendencies as controls and all others as cases.

## Supporting Information

Table S1 All significant and marginal SNPs for the 22 studies. All SNPs with p-values under 10^−6^ for the 22 studies.
(0.04 MB XLS)

Text S1 Selection of study individuals by ancestry.
(1.17 MB PDF)

Text S2 Relatedness.
(0.06 MB PDF)

Text S3 Genotyping and SNP quality control.
(0.04 MB PDF)

Text S4 Phenotypes.
(0.06 MB PDF)

Text S5 Questions for individual phenotypes.
(0.18 MB PDF)

Text S6 Details for specific associations.
(6.54 MB PDF)
