# Supplementary material for: Web-Based, Participant-Driven Studies Yield Novel Genetic Associations for Common Traits
Source: PLoS Genet. 2010 Jun 24;6(6):e1000993. doi: 10.1371/journal.pgen.1000993 (PMC2891811; doi:10.1371/journal.pgen.1000993)
Supplement: Text S1 — Selection of study individuals by ancestry. (1.17 MB PDF) [file pgen.1000993.s002.pdf]

Web-based, Participant-driven Studies Yield Novel Genetic Associations for Common Traits

Eriksson, Macpherson, Tung, Hon, Naughton, Saxonov, Avey, Wojcicki, Pe'er, Mountain

PLoS Genetics, 2010

S.1 Selection of Study Individuals by Ancestry

To avoid spurious associations due to population structure, we selected a subset of unrelated individuals having northern European ancestry from the wider dataset using multidimensional scaling (MDS), via the MDS routine implemented in the `R` function `cmdscale` [1]. This procedure is functionally equivalent to principal component analysis (the difference being that we used the allele-sharing distance (ASD, definition below) instead of the Euclidean distance as input to the MDS), therefore we abuse notation slightly in the main paper and call the MDS coordinates the principal components.

In order to find this subset, we computed the two-dimensional MDS over a set of genotypes comprising those of from the unrelated 23andMe dataset described above with the addition of the 1043 HGDP-CEPH individuals [2] and 326 individuals of self-reported European ancestry from Illumina's public genotype database iControlDB[1]. All iControlDB-derived individuals have four grandparents born in the same country. The ancestry information corresponding to these genotypes was provided by Peter Gregersen (personal communication).

The between-individual distance supplied to the MDS routine was the allele-sharing distance (ASD), $P_0 + P_1/2$, where P_k represents the proportion of loci at which the individuals shared exactly k alleles identical in state. If either of the SNP genotypes in a pair was missing, that locus was ignored in that pairwise calculation. Pairwise ASD was computed over a subset of the autosomal SNPs with no-call rates less than 1% in the participant and reference individual dataset. To minimize artifacts due to large regions of LD, we excluded several genomic regions known, likely or observed to exhibit long-range LD, including the HLA region, the *LCT* region, and the 23 polymorphic inversions of length greater than 500kb from the Database of Genomic Variants, version 6, for Build 36 (Nov 2008) [3]; the locations of the removed regions are shown in Table 1. We also required that no two SNPs were within 0.001 cM of each other, according to the fine-scale genetic map provided by HapMap [4]. After these filtering procedures, 313,847 SNPs remained, from an initial set of 547,347 autosomal SNPs that were common to both the participant and reference datasets.

All individuals from both the CEPH and iControlDB datasets were labeled as "European" or "non-European". In the case of CEPH, the dataset includes designations from seven world biogeographic regions, including Europe, Central/South Asia, East Asia, Near East, Africa, Oceania, and the Americas [2]; we thus labeled CEPH individuals given the designation Europe as "European", and all others "non-European". In the case of iControlDB, all individuals were labeled European. Only CEPH individuals from the "H971" reference subset, which contains no individuals with a first-degree relationship (parent/offspring or full siblings) were used [5]. All reference individuals and participants were projected together into a two-dimensional MDS configuration. We then removed individuals from the dataset if they did not cluster with the European-labeled reference individuals in the MDS projection. To determine cluster membership, we used the support vector machine classifier implemented in `libsvm 2.86` [6] on the two MDS axes (`libsvm` control parameters: $C = 32$, $\gamma = 2$; these were determined by fivefold cross-validation on the reference dataset's MDS coordinates.)

This procedure yielded a set of individuals likely to have near-complete European ancestry. We then repeated the above procedure on the genotypes from the putatively Europe-descended subset and the Europe-labeled reference individuals, this time augmented with 171 customers who reported four European or Ashkenazi grandparents. The European reference individuals and the 171 customer references were labeled as either "northern European", "Ashkenazi", "southern European", or "eastern European"

[1] `http://www.illumina.com/pages.ilmn?ID=231`

Annotation	Chrom.	Start (bp)	End (bp)
HLA	6	29000000	33000000
LCT	2	136200000	136400000
Inversion	1	141482868	142096907
Inversion	1	144543331	145858228
Inversion	3	12127602	12876345
Inversion	3	196882966	198870687
Inversion	5	69770285	70439401
Inversion	7	5948840	6827030
Inversion	8	126775889	129803035
Inversion	8	7207070	12500379
Inversion	9	66196555	67903533
Inversion	9	68448984	70110408
Inversion	9	85701736	87641082
Inversion	13	24476805	25541515
Inversion	13	99738307	100312195
Inversion	14	18537960	19135372
Inversion	15	18870124	20077222
Inversion	15	28524207	30669954
Inversion	15	72151413	73356183
Inversion	16	21485316	22615829
Inversion	16	32061335	32742620
Inversion	16	32906698	33539015
Inversion	17	16658684	18263022
Inversion	17	31888441	33603080
Inversion	17	40899921	41989253

Table 1. Genomic regions excluded from ASD computation.

Label	Countries, Regions, or Ethnicities included
northern European	Ireland, Netherlands, Norway, France, Germany, Sweden, United Kingdom, Switzerland, Denmark, Austria, Belgium
Ashkenazi	Ashkenazi Jewish descent
southern European	Italy, Tuscany, North Italy, Greece, Spain, Greece
eastern European	Finland, Estonia, Romania, Slovakia, Slovenia

Table 2. Countries or regions used as the basis for SVM training within Europe. Individuals from the CEPH, iControlDB, and customer reference populations who reported four grandparents from the given region/ethnicity were included in the training set.

according to Table 2. A second two-dimensional MDS was run, and a second SVM classification was obtained (`libsvm` control parameters: $C = 8$, $\gamma = 0.5$; determined as above, but over the set of reference individuals remaining after the first SVM classification). This yielded a set of individuals classified as northern European.

References

1. R Development Core Team (2007) R: A Language and Environment for Statistical Computing. R Foundation for Statistical Computing, Vienna, Austria. URL http://www.R-project.org. ISBN 3-900051-07-0.

2. Cann HM, de Toma C, Cazes L, Legrand MF, Morel V, et al. (2002) A human genome diversity cell line panel. Science 296: 261–262.

3. Iafrate AJ, Feuk L, Rivera MN, Listewnik ML, Donahoe PK, et al. (2004) Detection of large-scale variation in the human genome. Nat Genet 36: 949–951.

4. International HapMap Consortium, Frazer KA, Ballinger DG, Cox DR, Hinds DA, et al. (2007) A second generation human haplotype map of over 3.1 million SNPs. Nature 449: 851–861.

5. Rosenberg NA (2006) Standardized subsets of the HGDP-CEPH Human Genome Diversity Cell Line Panel, accounting for atypical and duplicated samples and pairs of close relatives. Ann Hum Genet 70: 841–847.

6. Chang CC, Lin CJ (2001) LIBSVM: a library for support vector machines. Software available at http://www.csie.ntu.edu.tw/~cjlin/libsvm.

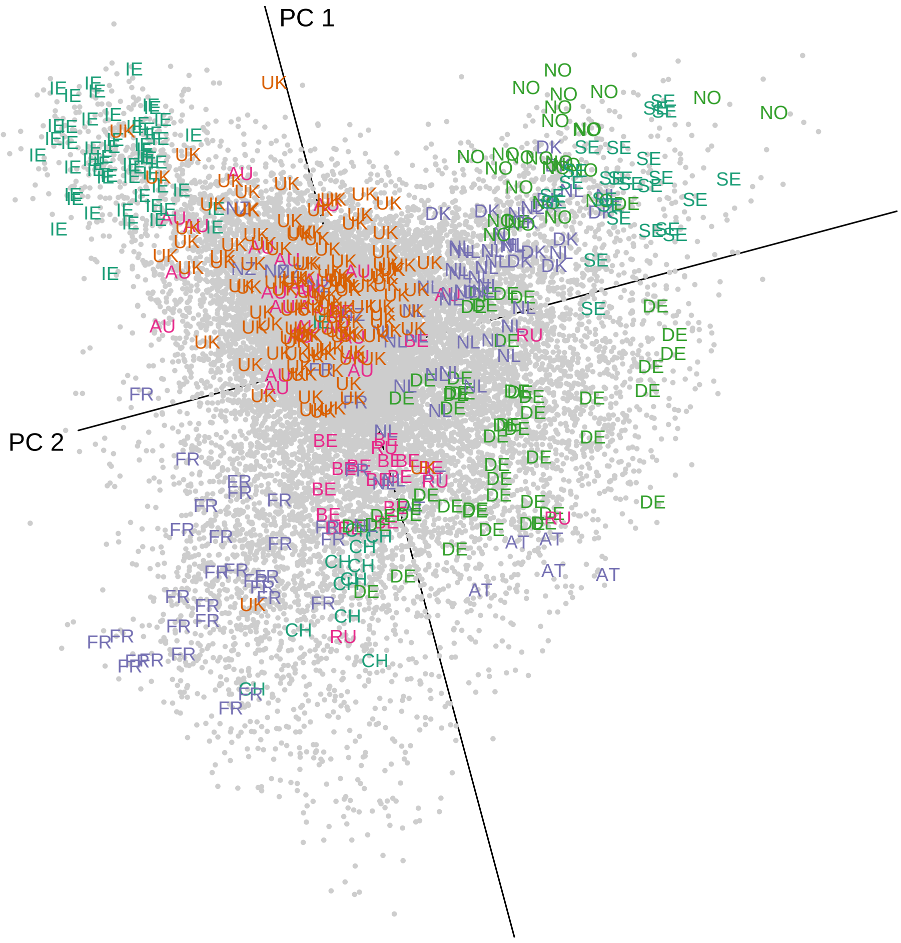

Figure 1. First two coordinates of variation for northern European participants. Those participants with all four grandparents born in the same country who provided ancestry information are highlighted in the plot. Two-letter country codes of these individuals are overlaid, as follows: AU – Australia, BE – Belgium, DE – Germany, FR – France, IE – Ireland, NL – The Netherlands, NO – Norway, SE - Sweden, UK – United Kingdom. Other participants are plotted as gray dots. The plot has been rotated 75° clockwise to make the geographic correspondence clearer.

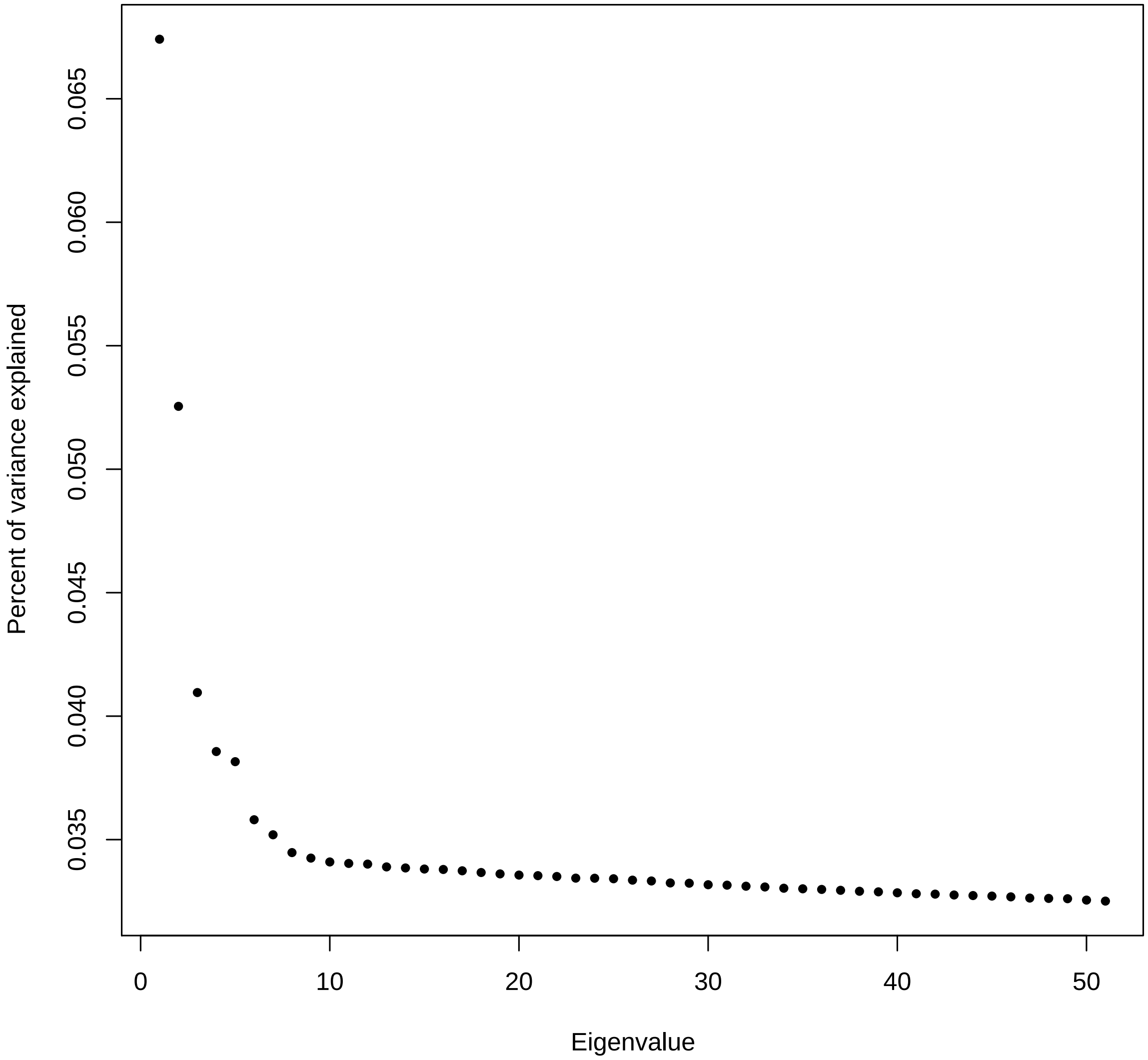

Figure 2. Percent of variance explained by MDS coordinates within northern European group.
