# Supplementary material for: Web-Based, Participant-Driven Studies Yield Novel Genetic Associations for Common Traits
Source: PLoS Genet. 2010 Jun 24;6(6):e1000993. doi: 10.1371/journal.pgen.1000993 (PMC2891811; doi:10.1371/journal.pgen.1000993)
Supplement: Text S2 — Relatedness. (0.06 MB PDF) [file pgen.1000993.s003.pdf]

Web-based, Participant-driven Studies Yield Novel Genetic Associations for Common Traits

Eriksson, Macpherson, Tung, Hon, Naughton, Saxonov, Avey, Wojcicki, Pe'er, Mountain

PLoS Genetics, 2010

S.2 Relatedness

We used a novel method to calculate identity by descent (IBD).Using unphased data, the algorithm finds evidence of an absence of IBD by looking for calls in which one genotype is homozygous for AA but the other genotype is homozygous for BB, showing that neither allele could have come from a shared ancestor. Therefore, by looking for stretches of a lack of "opposite homozygotes," we can define regions that contain at least half IBD. To increase the confidence that a given region contains IBD, we require that the region be at least 5 cM and contains 400 genotyped SNPs, to make sure that there is both sufficient genotype coverage and genetic distance defining the IBD region. We minimized the effects of genotyping error in two ways: first, we filtered out SNPs that had a large number of Mendelian errors when comparing data from father-mother-child trios, had a high no call rate, or otherwise failed quality control measures. Second, in an IBD segment, we allowed for occasional opposite homozygotes if there was at least 3 cM and 300 SNPs surrounding the opposite homozygote that was free of opposite homozygotes.

Using IBD, we defined a set of individuals in which all pairs of individuals were guaranteed to be more distant than a first cousin relationship. Looking at the distribution of at least half IBD between customer pairs in Figure 1(a), first cousins clustered around the expected amount of shared half IBD of 900 cM, with a minimum of approximately 700 cM. We therefore chose 700 cM (or 19.4% half IBD) as the threshold for relatedness. To maximize the size of the set of individuals conforming to these criteria, we employed a greedy search that preferentially chooses grandparent and parent relationships derived trio data, and further prefers individuals who have answered multiple surveys.

To understand the relationship between IBD and ASD (allele-sharing distance, defined in Supplement, Text S1), we looked at their correlation in Figure 1(b). IBD correlates well with ASD (correlation of -0.957), but shows greater sensitivity in relatively distant relationships.

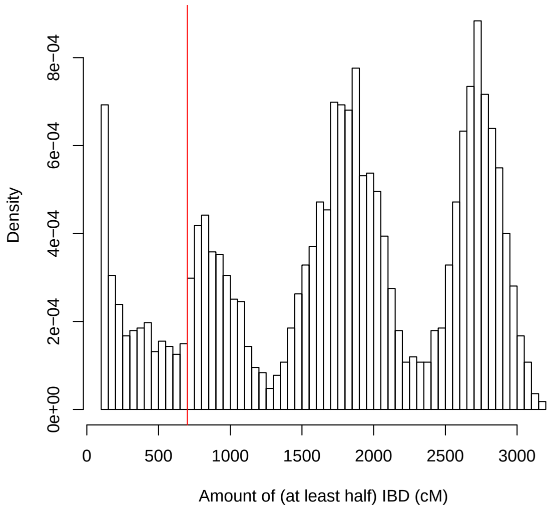

(a) The three clusters centered around 900, 1800, and 2700 cM represent 1st cousins, grandparent/grandchildren and avuncular relationships, and sibling relationships, respectively. The red line at 700 cM denotes the edge of the 1st cousin cluster.

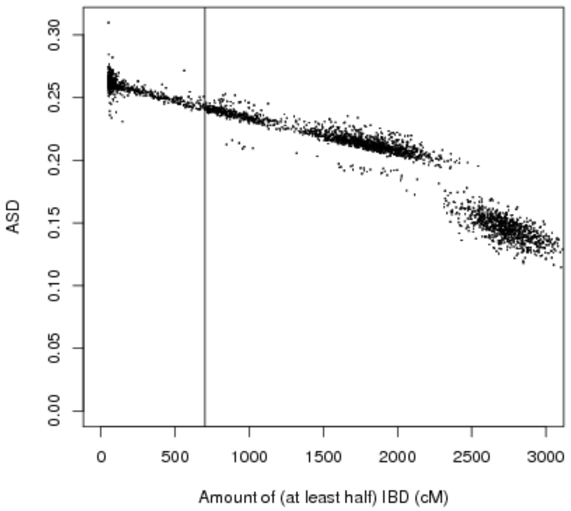

(b) Amount of IBD versus ASD for all pairs of participants with greater than 50cM IBD.

Figure 1. Distribution of half or greater IBD among participants.
