# Supplementary material for: Web-Based, Participant-Driven Studies Yield Novel Genetic Associations for Common Traits
Source: PLoS Genet. 2010 Jun 24;6(6):e1000993. doi: 10.1371/journal.pgen.1000993 (PMC2891811; doi:10.1371/journal.pgen.1000993)
Supplement: Text S3 — Genotyping and SNP quality control. (0.04 MB PDF) [file pgen.1000993.s004.pdf]

Web-based, Participant-driven Studies Yield Novel Genetic Associations for Common Traits

Eriksson, Macpherson, Tung, Hon, Naughton, Saxonov, Avey, Wojcicki, Pe'er, Mountain

PLoS Genetics, 2010

S.3 Genotyping and SNP Quality Control

Samples were genotyped on two slightly different versions of the Illumina HumanHap550+ BeadChip platform. The first version of the BeadChip included 561,466 SNPs from the 550 v3 Illumina panel and additional 25,536 custom SNPs. Another subset of customers was genotyped on the second version of the chip, which included 560,136 SNPs from the 550Quad v1 Illumina panel and an additional set of 24,811 custom SNPs. The two platforms had 567,205 SNPs in common. For individuals that were genotyped on both versions of the BeadChip, we used a union of their datasets in our analyses. Calls from version 2 of the platform took precedence over those of version 1. Where version 2 had a no-call the call from version 1 was used.

Several quality control measures were used to pare down the set of SNPs used in the analysis. The initial production of the custom platform was accompanied by the genotyping of over 200 HapMap samples. SNPs with call rates below 40% were eliminated. It should be noted that we were generous with the call-rate threshold at this stage to allow for numerous haploid (Y and mtDNA) SNPs, which tend to yield high no-call rates with Illumina's Beadstudio software. Calls from the initial HapMap run were compared against the calls published by the HapMap consortium. SNPs with high rates (over 1%) of discordances were manually examined and excluded if their cluster plots appeared questionable. In addition, cluster plots for approximately 4,000 SNPs were examined and SNPs of questionable quality were eliminated from further analyses. Two additional checks were performed to eliminate SNPs with potential problems due to incorrect annotations in dbSNP. We removed SNPs that appeared monomorphic and whose flanking sequences in dbSNP did not match the reference genome sequence. We also excluded SNPs whose dbSNP entries suggested that they were tri-allelic but our chip only had probes to assay two of the alleles. In total, all of these measures eliminated 8,982 SNPs from version 1 and 6,598 from version 2 of our genotyping platform.

Subsequent quality control measures were applied on the basis of the entire dataset used in this analysis. For example, SNPs were checked for impossible patterns of inheritance in all trios within the 23andMe customer database. The 1553 that exhibited such a pattern in at least 1% of analyzed trios were excluded.
