# Supplementary material for: Web-Based, Participant-Driven Studies Yield Novel Genetic Associations for Common Traits
Source: PLoS Genet. 2010 Jun 24;6(6):e1000993. doi: 10.1371/journal.pgen.1000993 (PMC2891811; doi:10.1371/journal.pgen.1000993)
Supplement: Text S4 — Phenotypes. (0.06 MB PDF) [file pgen.1000993.s005.pdf]

Web-based, Participant-driven Studies Yield Novel Genetic Associations for Common Traits

Eriksson, Macpherson, Tung, Hon, Naughton, Saxonov, Avey, Wojcicki, Pe'er, Mountain

PLoS Genetics, 2010

S.4 Phenotypes

All surveys released between May and October 2008 are shown in Table 1. Correlations between phenotypes and covariates are shown in Table 2.

Survey	Date	Traits analyzed
“Ten Things About You”	May 27, 2008	asparagus odor, photic sneeze, hair curl, morning/evening preference, hand-clasp, glasses, cavities, sweet-tooth, *handedness*, *freckling*
“Ocular Dominance”	June 25, 2008	ocular dominance
“Handedness”	June 25, 2008	handedness
“Optimism”	September 3, 2008	optimism
“Ten More Things About You”	September 3, 2008	attached earlobes, wisdom teeth, motion sickness in a car, astigmatism, braces, *motion sickness on a boat*, *motion sickness on a plane*, *tendency to jiggle ones leg*, *hair whorl direction*, *eyelid crease*
“Footedness”	October 2, 2008	footedness
“Pigmentation”	October 8, 2008	eye color, green eye color, hair color, red hair, freckling
“Tortoise or Hare?”	May 27, 2008	*preference for sprint/endurance exercise*
“Feeling Flush”	May 27, 2008	*alcohol flush*
“A Hint of Bitterness”	May 27, 2008	*bitter taste detection*
“A Sticky Question”	May 27, 2008	*type of earwax*
“Does Milk Do Your Body Good?”	May 27, 2008	*lactose intolerance*
“The Eyes Have It”	July 31, 2008	*eye color*

Table 1. All surveys released with derived phenotypes. The phenotypes that did not meet our criteria and thus were not analyzed in this paper are italicized. The last six surveys were displayed alongside predictions the related traits based on customer genotypes.

Name	sex	age	PC1	PC2	PC3	PC4	PC5
asparagus	0.064 (0.294)	0.012 ($6.3 \cdot 10^{-9}$)	-19.031 (0.009)	21.629 (0.005)	-32.795 ($4.7 \cdot 10^{-4}$)	4.374 (0.644)	-0.660 (0.946)
cavities	-0.051 ($2.2 \cdot 10^{-4}$)	-0.014 ($3.0 \cdot 10^{-179}$)	-0.502 (0.754)	-0.281 (0.869)	0.004 (0.999)	0.436 (0.838)	-2.332 (0.284)
eyecolor	0.240 ($6.1 \cdot 10^{-5}$)	-0.007 ($4.9 \cdot 10^{-4}$)	66.483 ($2.7 \cdot 10^{-20}$)	-41.371 ($4.0 \cdot 10^{-8}$)	13.589 (0.135)	2.977 (0.748)	-12.406 (0.187)
green-eyecolor	0.432 ($1.9 \cdot 10^{-8}$)	-0.010 ($1.1 \cdot 10^{-4}$)	18.867 (0.055)	-7.094 (0.456)	-6.349 (0.583)	-4.157 (0.727)	-0.012 (0.999)
footedness	1.444 ($7.6 \cdot 10^{-7}$)	0.023 (0.016)	74.937 (0.030)	-17.507 (0.628)	11.132 (0.799)	-53.058 (0.240)	57.920 (0.203)
freckle	0.700 ($1.5 \cdot 10^{-14}$)	-0.025 ($1.9 \cdot 10^{-16}$)	-113.337 ($3.8 \cdot 10^{-25}$)	-54.356 ($2.0 \cdot 10^{-6}$)	32.325 (0.020)	-8.568 (0.542)	-16.010 (0.263)
red-hair	0.368 ($2.0 \cdot 10^{-57}$)	-0.003 ($1.8 \cdot 10^{-5}$)	-12.527 ($4.5 \cdot 10^{-6}$)	-10.279 ($3.4 \cdot 10^{-4}$)	3.937 (0.255)	-3.210 (0.362)	-3.315 (0.355)
haircolor	-0.563 ($2.8 \cdot 10^{-19}$)	-0.009 ($5.5 \cdot 10^{-5}$)	14.606 (0.040)	-81.260 ($4.8 \cdot 10^{-26}$)	31.202 ($8.3 \cdot 10^{-4}$)	-22.825 (0.016)	6.419 (0.511)
haircurl	0.141 ($2.1 \cdot 10^{-8}$)	-0.002 (0.006)	5.050 (0.081)	-18.880 ($1.1 \cdot 10^{-9}$)	6.218 (0.099)	-0.212 (0.956)	2.864 (0.468)
handedness	-0.291 ($1.4 \cdot 10^{-6}$)	-0.008 ($1.6 \cdot 10^{-4}$)	-0.845 (0.905)	-5.946 (0.425)	-0.252 (0.978)	5.067 (0.586)	-12.442 (0.185)
astigmatism	0.333 ($6.1 \cdot 10^{-13}$)	0.013 ($2.2 \cdot 10^{-16}$)	7.976 (0.150)	5.873 (0.314)	-6.018 (0.390)	-0.849 (0.906)	5.259 (0.469)
braces	-0.221 ($8.0 \cdot 10^{-4}$)	0.035 ($4.2 \cdot 10^{-52}$)	-4.405 (0.576)	-1.070 (0.897)	10.615 (0.287)	-13.238 (0.191)	-6.759 (0.513)
glasses	-0.372 ($1.8 \cdot 10^{-6}$)	-0.057 ($1.7 \cdot 10^{-90}$)	-9.922 (0.247)	-0.180 (0.984)	-3.831 (0.734)	-4.572 (0.698)	-8.303 (0.487)
handclasp	0.120 (0.033)	0.008 ($9.0 \cdot 10^{-5}$)	9.820 (0.131)	-3.325 (0.631)	-5.224 (0.538)	-11.629 (0.181)	-5.238 (0.555)
morningness	0.184 (0.004)	0.041 ($2.7 \cdot 10^{-67}$)	7.135 (0.341)	-9.813 (0.223)	-28.673 (0.003)	-6.847 (0.494)	16.263 (0.111)
ocular-dominance	0.009 (0.902)	0.004 (0.111)	14.422 (0.104)	6.291 (0.493)	7.582 (0.497)	-3.867 (0.738)	1.141 (0.922)
sneeze	-0.020 (0.738)	-0.005 (0.007)	-4.565 (0.503)	1.821 (0.802)	-12.885 (0.147)	0.281 (0.975)	-2.516 (0.786)
sweettooth	0.147 (0.042)	-0.001 (0.654)	-1.371 (0.870)	8.661 (0.329)	0.536 (0.961)	-0.268 (0.981)	-2.918 (0.801)
wisdom-teeth	-0.338 ($1.5 \cdot 10^{-6}$)	0.008 ($4.8 \cdot 10^{-4}$)	-5.077 (0.545)	4.906 (0.575)	8.196 (0.436)	0.668 (0.951)	-14.854 (0.175)
earlobes	-0.215 (0.004)	-0.002 (0.326)	-3.618 (0.686)	-8.271 (0.374)	-7.018 (0.532)	-7.614 (0.507)	1.087 (0.926)
motionsick	0.634 ($1.7 \cdot 10^{-15}$)	-0.010 ($1.3 \cdot 10^{-4}$)	9.084 (0.341)	-17.676 (0.082)	-7.536 (0.538)	-12.583 (0.311)	-5.843 (0.640)

Table 2. Regression coefficients (p-values) for phenotypes on covariates. Sex was coded as 0 = male and 1 = female.
