# Supplementary material for: Web-Based, Participant-Driven Studies Yield Novel Genetic Associations for Common Traits
Source: PLoS Genet. 2010 Jun 24;6(6):e1000993. doi: 10.1371/journal.pgen.1000993 (PMC2891811; doi:10.1371/journal.pgen.1000993)
Supplement: Text S5 — Questions for individual phenotypes. (0.18 MB PDF) [file pgen.1000993.s006.pdf]

Web-based, Participant-driven Studies Yield Novel Genetic Associations for Common Traits

Eriksson, Macpherson, Tung, Hon, Naughton, Saxonov, Avey, Wojcicki, Pe'er, Mountain

PLoS Genetics, 2010

S.5 Questions for individual phenotypes

S.5.1 Hair curl

Participants were asked one question about hair curl: "Is your hair naturally straight or curly?" The available answer choices and their codings are shown in Figure 1. Answer choices were presented as a picture with descriptive text but without the coding. Analysis used these codings in a linear regression.

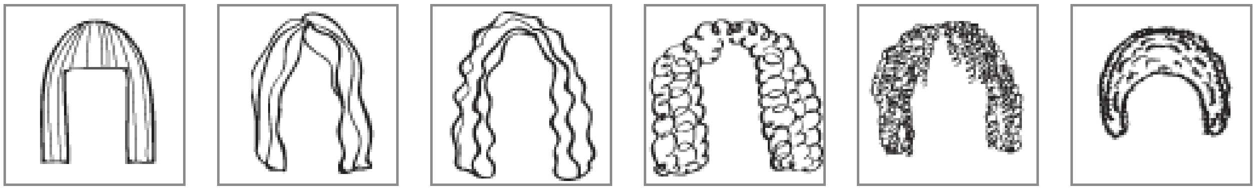

Stick straight	Slightly wavy	Wavy	Big curls	Small curls	Very tight curls
0	1	2	3	4	5

Figure 1. Pictures used to assess hair curl

S.5.2 Freckling

Participants were asked the following questions, with options shown in Figure 2. The numerical scores were not displayed on the questionnaire.

- The freckling (not moles—moles can be present at birth and can form even in parts of the body not exposed to the sun) on my face is most similar to:
- The freckling (not moles) on my arms is most similar to:
- The freckling (not moles) on my shoulders is most similar to:

S.5.3 Hair color

The hair color phenotypes were based on two questions. In addition, a third question was used in order to test the consistency of responses.

1. I would describe the amount of red in my hair (before I went gray, if I am gray now) as:
   - No red at all
   - A tinge of red
   - Some red
   - A lot of red
2. The natural color of my hair (before I went gray, if I am gray now) is most similar to:
   - I don't know

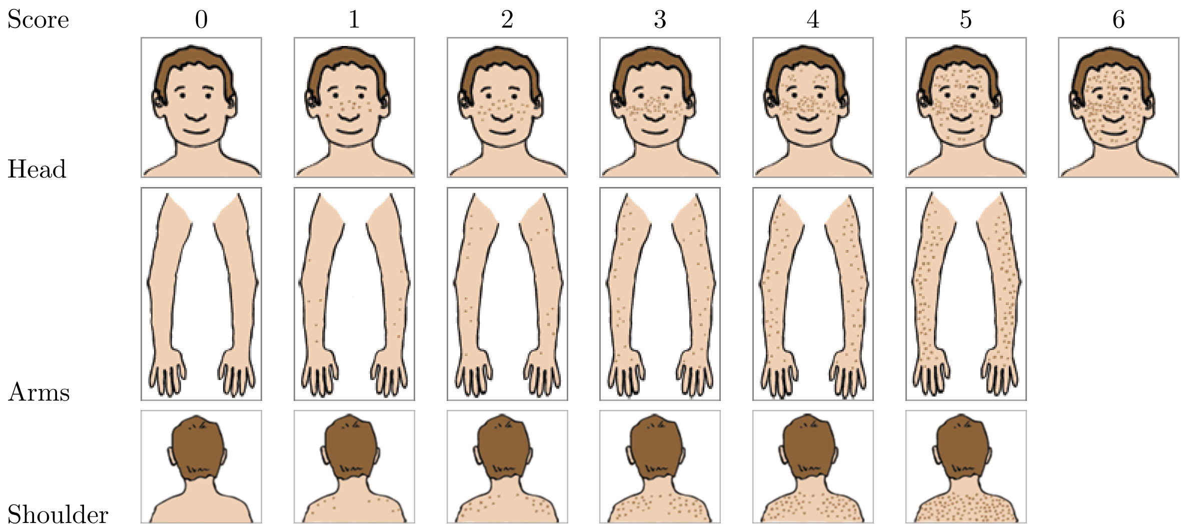

Figure 2. Freckling was scored on a scale from 0 to 16 as the sum of the scores on the face, arms, and shoulders.

- One of the 9 pictures in Figure 3.

3. I would describe the natural color of my hair (before I went gray, if I am gray now) as:
    - Light blond
    - Dark blond
    - Red
    - Light brown
    - Dark brown
    - Black
    - I don't know

S.5.4 Eye color

The eye color analyses were based on participants matching their eye color to a series of pictures.

They were asked to "Look very closely at the color of your eyes, preferably under bright natural light. Then complete the following: the image that most closely resembles the color of my eyes is:" with the pictures in Figure 4 (without text) as answer options, along with "None of the below, because my eyes are totally different colors from each other." People giving this answer were removed.

S.5.5 Muscle performance

The question "Do you think you'd perform better in a sprint or in a longer distance race?" was asked. Available answers were

- Sprint race
- Longer distance race

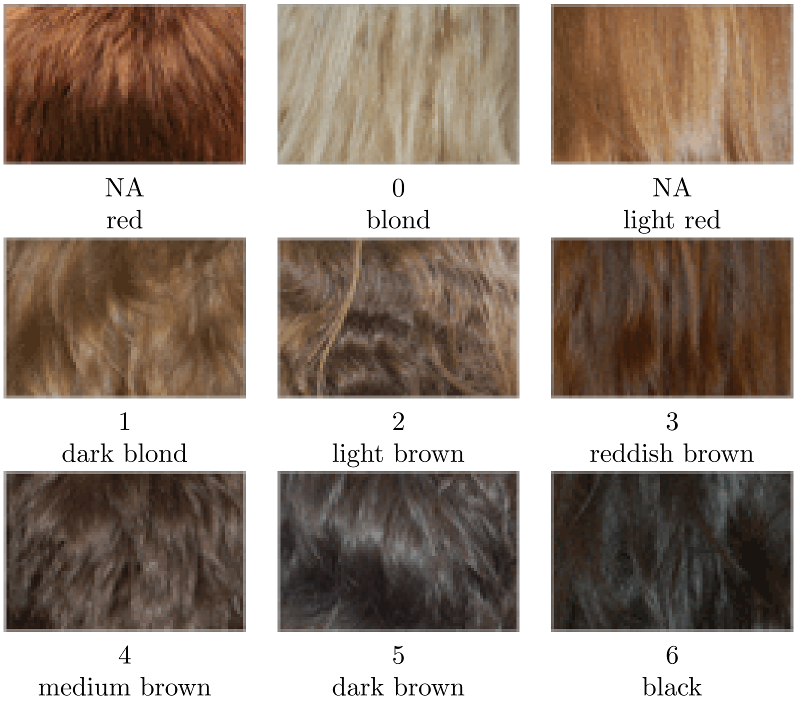

Figure 3. Images for hair color used for the blond to brown analysis excluding red. Scorings are shown here but were not shown on the questionnaire.

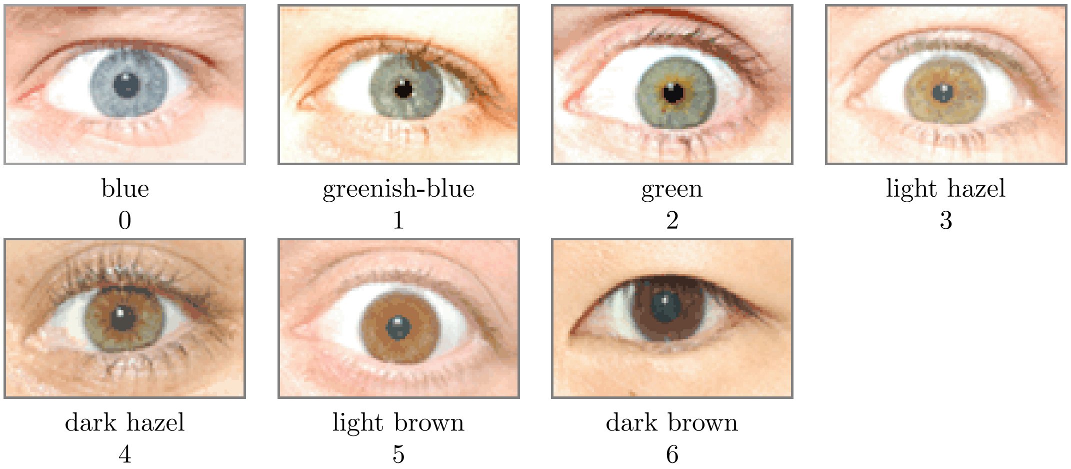

Figure 4. Images for eyecolor. Eyecolor was scored on a scale from 0 to 6.

	Genotypes Unseen		Genotypes Seen	
	CC or TC	TT	CC or TC	TT
Sprint	480	107	1052	113
Endurance	386	103	530	290

Table 1. Counts of respondents by genotype at rs1815739 according to whether they had seen a prediction based on their genotype or not.

- Both—I'm like the wind
- Neither

For the analysis (Results, Phenotype Data), people answering "Both" or "Neither" were disregarded.

References

1. Elias LJ, Bryden MP, Bulman-Fleming MB (1998) Footedness is a better predictor than is handedness of emotional lateralization. Neuropsychologia 36: 37-43.
2. Annett M (1970) A classification of hand preference by association analysis. Br J Psychol 61: 303–321.
3. Miles WR (1929) Ocular dominance demonstrated by unconscious sighting. J Exp Psych : 113–126.
4. Scheier MF, Carver CS, Bridges MW (1994) Distinguishing optimism from neuroticism (and trait anxiety, self-mastery, and self-esteem): a reevaluation of the life orientation test. J Pers Soc Psychol 67: 1063-1078.

Phenotype	Question	Scoring
Astigmatism	“Have you ever been told by a doctor that you have astigmatism?”	Binary
Braces	“Have you ever been told by a dentist that you needed braces to straighten your teeth?”	Binary
Cavities	“Have you ever had any cavities?”	Scored as QT: 2 = Yes, more than three / 1 = Yes, but three or fewer / 0 = No, I’m a dental superstar! / NA = I don’t know
Earlobes	”Are your earlobes attached or unattached?”	Binary
Footedness	10 question survey from [1]	Scored as QT
Glasses	“Have you ever had to wear contact lenses or eye glasses?”	Binary
Hand-clasp	“When you clasp your hands together comfortably, which thumb is on top?”	Binary
Handedness	survey from [2]	8 point scoring system from [2]
Morning/evening preference	“Are you naturally a night person or a morning person?”	Night owl / Early bird / Neither
Motion sickness	“Have you experienced motion sickness while riding in a car (car sickness)?”	CASE = Yes, I do now frequently / CASE = Yes, I did frequently, but only as a child / NA = Yes, occasionally / CONTROL = No / NA = Don’t know
Ocular dominance	single question from [3]	Binary
Optimism	survey from [4]	scored as QT
Wisdom teeth	“Have you ever been told by a dentist that you needed to have your wisdom teeth removed for medical reasons?”	Binary

Table 2. Questions for traits not described elsewhere.
