# Supplementary material for: Web-Based, Participant-Driven Studies Yield Novel Genetic Associations for Common Traits
Source: PLoS Genet. 2010 Jun 24;6(6):e1000993. doi: 10.1371/journal.pgen.1000993 (PMC2891811; doi:10.1371/journal.pgen.1000993)
Supplement: Text S6 — Details for specific associations. (6.54 MB PDF) [file pgen.1000993.s007.pdf]

Web-based, Participant-driven Studies Yield Novel Genetic Associations for Common Traits

Eriksson, Macpherson, Tung, Hon, Naughton, Saxonov, Avey, Wojcicki, Pe'er, Mountain

PLoS Genetics, 2010

S.6 Details for specific associations

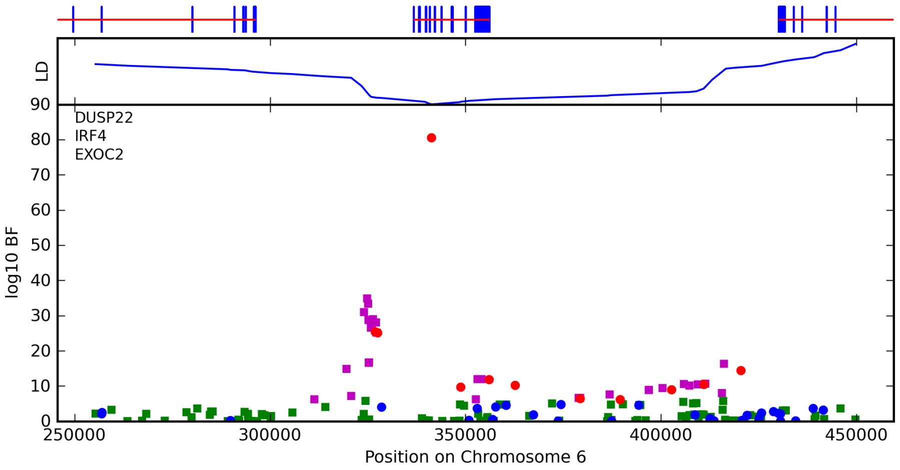

(a) Bayes factors around IRF4

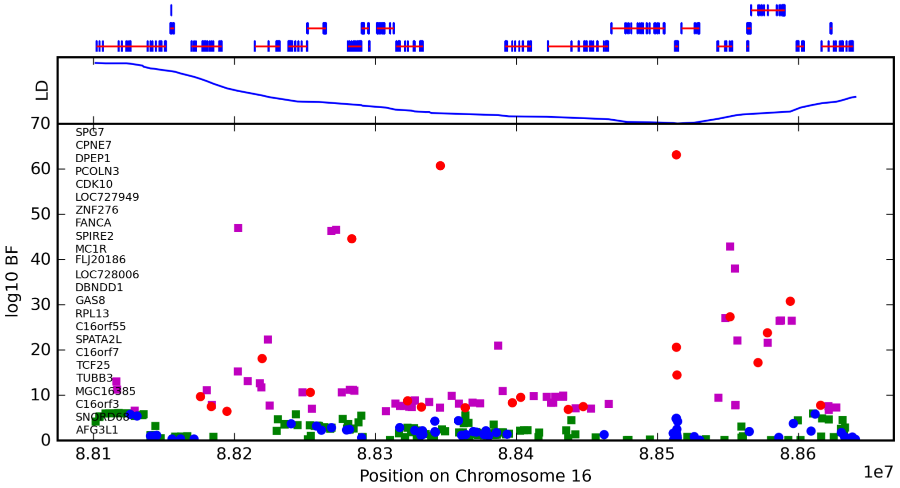

(b) Bayes factors around MC1R

Figure 1. Bayes factors for freckling around *IRF4* and *MC1R*

Phenotype	Size	Cases	Controls	Covariates	λ	Top hit	# Loci	Loci
Eye color, blue to brown	4402	—	—	pca, sex, age	1.00	>300	6	OCA2/HERC2, SLC24A4, IRF4, SLC45A2, TYR, (TYRP1)
Freckles	4405	—	—	pca, sex, age	1.01	90.68	5	*IRF4*, MC1R, ASIP, **BNC2**, (TYR)
Hair color, blond to brown	3044	—	—	pca, sex, age	1.01	87.07	5	OCA2/HERC2, IRF4, SLC45A2, SLC24A4, MC1R
Red hair	4422	—	—	pca, sex, age	1.01	86.28	2	MC1R, ASIP
Eye color, green/blue	2826	1505	1321	sex, age	1.00	51.52	3	OCA2/HERC2, SLC24A4, TYR
Hair curl	5385	—	—	pca, sex, age	1.02	41.80	3	TCHH, **WNT10A**, (**OFCC1**)
Asparagus anosmia	4742	1737	3005	pca, age	1.02	23.18	1	**OR2M7**
Photic sneeze reflex	5390	1750	3640	age	1.02	10.93	2	**2q22.3**, (**NR2F2**)
Footedness	3079	—	—	pca, sex, age	1.00	6.75	0	
Attached earlobes	3915	2940	975	sex	1.01	6.59	0	
Morningness	4264	2028	2237	pca, sex, age	1.01	6.50	0	
Braces	4011	1951	2062	sex, age	1.01	6.45	0	
Optimism	3936	—	—	sex, age	1.00	6.29	0	
Astigmatism	7701	3982	3727	sex, age	1.01	6.17	0	
Prefer sweet snacks	3100	1484	1616	sex	1.03	6.07	0	
Wisdom teeth	3983	1210	2773	sex, age	1.00	5.89	0	
Cavities	5366	—	—	sex, age	1.01	5.81	0	
Glasses	5386	947	4440	sex, age	1.00	5.76	0	
Ocular dominance	3126	1956	1170	None	1.00	5.70	0	
Hand-clasp	5256	2280	2977	sex, age	1.00	5.66	0	
Motion sickness	2987	963	2024	sex, age	1.02	5.55	0	
Handedness	4268	—	—	sex, age	1.00	5.30	0	

Table 1. Statistics on the 22 studies (with or without associations reaching genome-wide significance). Loci are called significant if they contain a SNP with $-\log_{10}$ p-value over 8.4 and suggestively significant if they have one between 7.1 and 8.4. Loci that were not previously associated with the given trait are in bold, those where we report a remapping of a previous hit are in italics, and suggestively significant loci are in parentheses. Size refers to the total number of individuals in the study. For binary traits, we additionally report the number of cases and controls . The listed covariates were included in the analysis (if "pca" is listed as a covariate the first five axes of genetic variation within northern Europe were used). The "top hit" refers to the largest $-\log_{10}$ p-value for the given trait. We report the genomic control inflation factor, λ, [1] for the analysis including covariates.

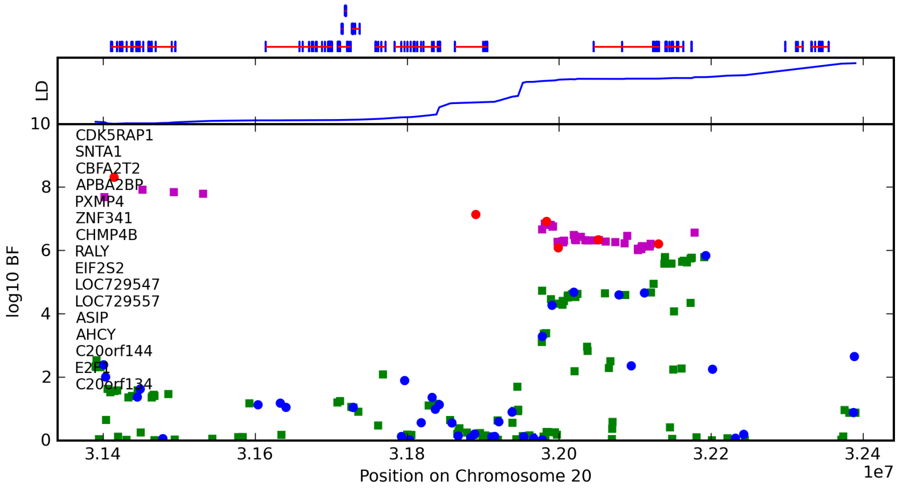

(a) Bayes factors around ASIP

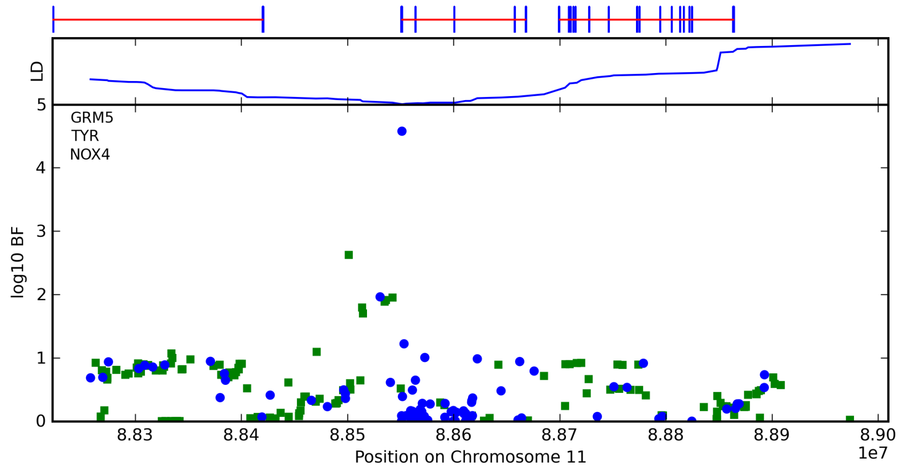

(b) Bayes factors around TYR

Figure 2. Bayes factors for freckling around *ASIP* and *TYR*

	TT (%)	TC (%)	CC (%)
0	391 (25.3)	628 (29.9)	233 (31.0)
1	242 (15.6)	375 (17.9)	137 (18.2)
2	267 (17.3)	383 (18.3)	151 (20.1)
3	173 (11.2)	227 (10.8)	77 (10.2)
4	108 (7.0)	133 (6.3)	37 (4.9)
5	89 (5.8)	88 (4.2)	33 (4.4)
6	65 (4.2)	56 (2.7)	20 (2.7)
7	50 (3.2)	48 (2.3)	22 (2.9)
8	42 (2.7)	42 (2.0)	9 (1.2)
9	26 (1.7)	25 (1.2)	11 (1.5)
10	20 (1.3)	26 (1.2)	7 (0.9)
11	18 (1.2)	17 (0.8)	6 (0.8)
12	20 (1.3)	17 (0.8)	2 (0.3)
13	11 (0.7)	9 (0.4)	1 (0.1)
14	11 (0.7)	9 (0.4)	4 (0.5)
15	8 (0.5)	6 (0.3)	0 (0.0)
16	6 (0.4)	9 (0.4)	2 (0.3)

Table 2. Counts for freckling versus genotype at rs2153271 (in *BNC2*). Higher scores correspond to more freckles.

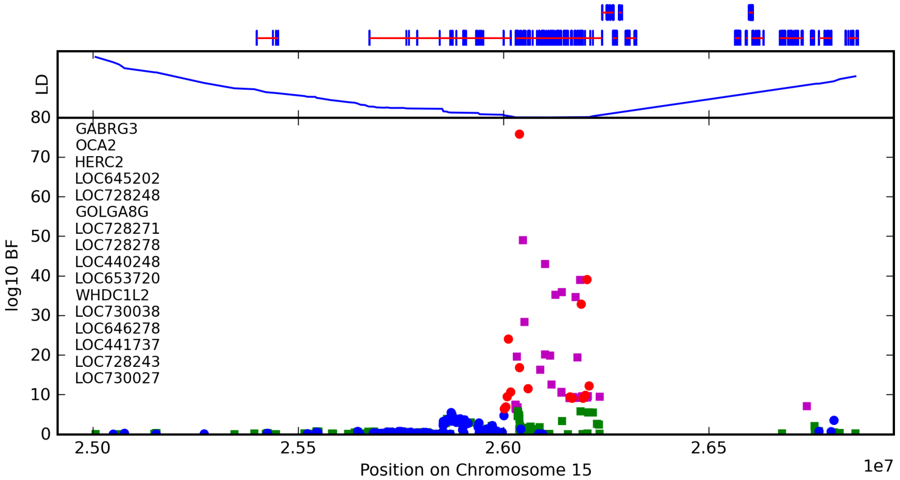

(a) Bayes factors around OCA2

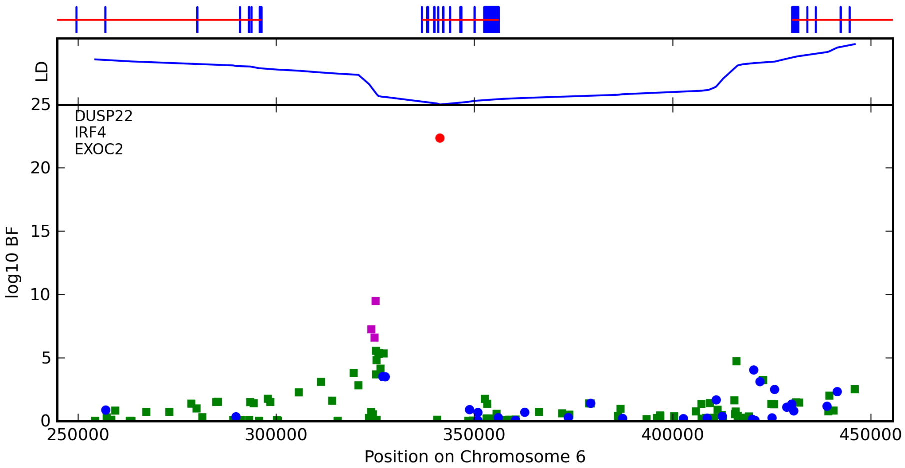

(b) Bayes factors around IRF4

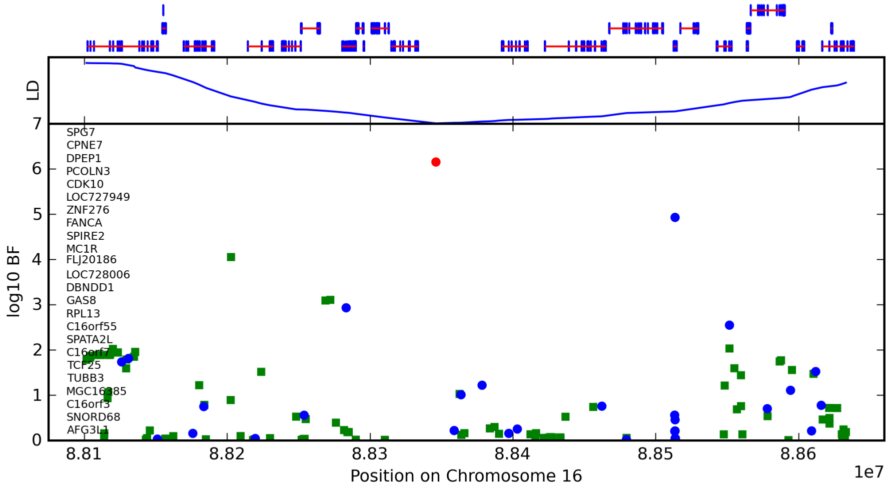

(c) Bayes factors around MC1R

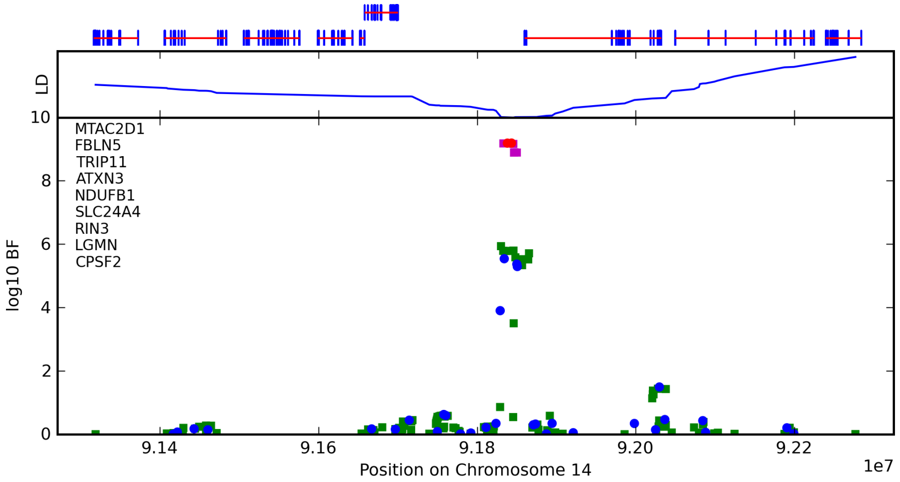

(d) Bayes factors around SLC24A4

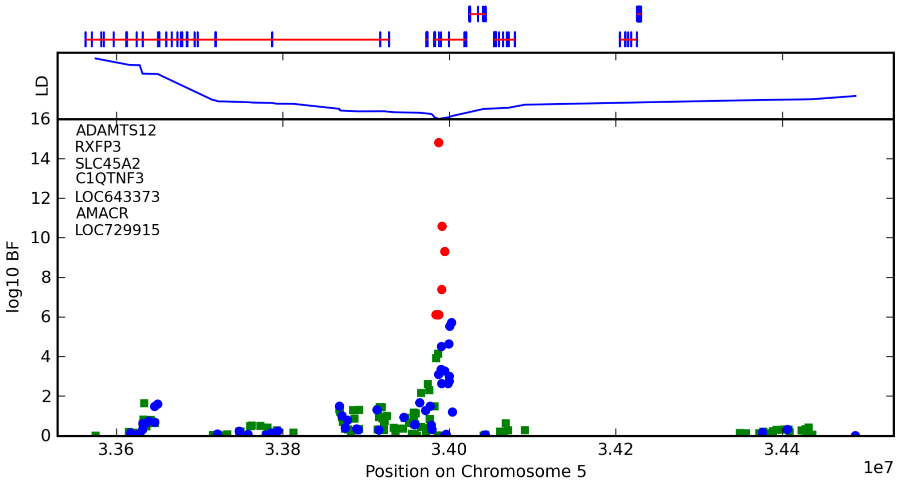

(e) Bayes factors around SLC45A2

Figure 3. Region plots for hair color, blond to brown

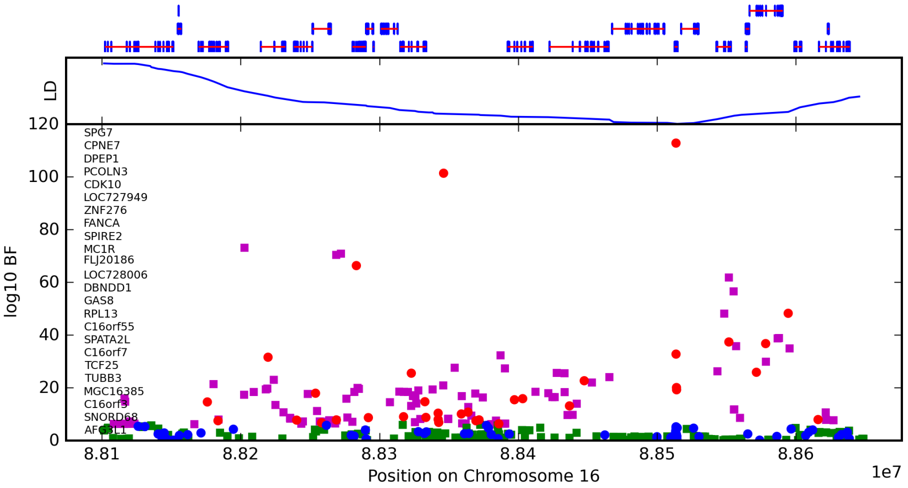

(a) Bayes factors around MC1R

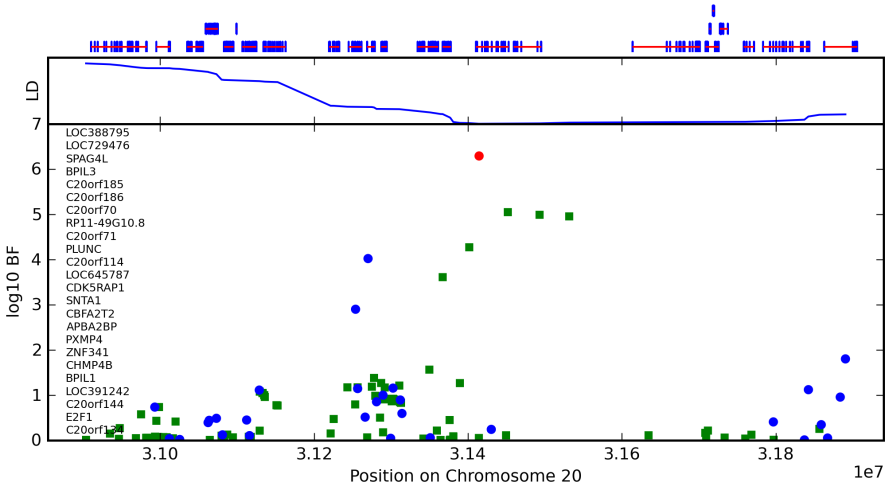

(b) Bayes factors around ASIP

Figure 4. Region plots for red hair color

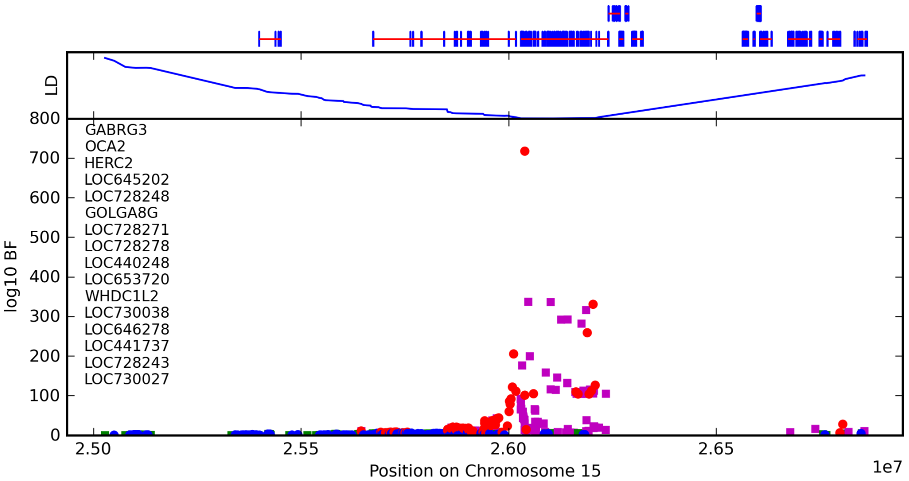

(a) Bayes factors around OCA2

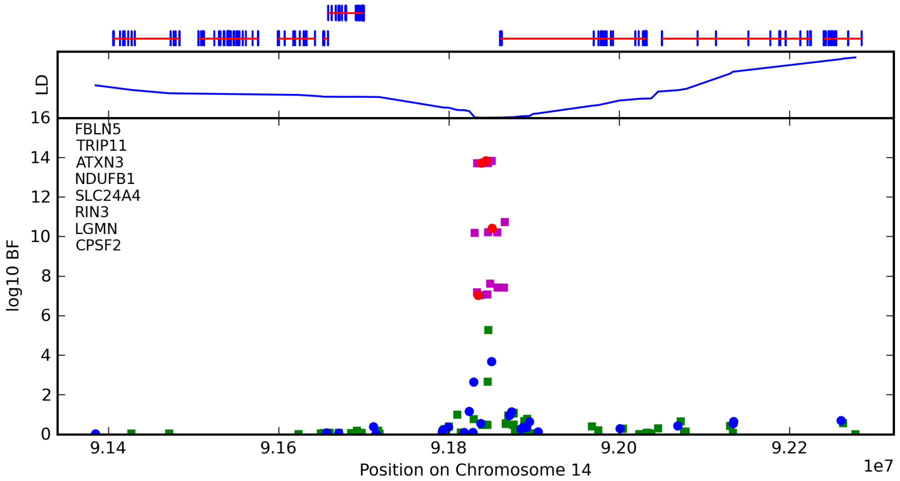

(b) Bayes factors around SLC24A4

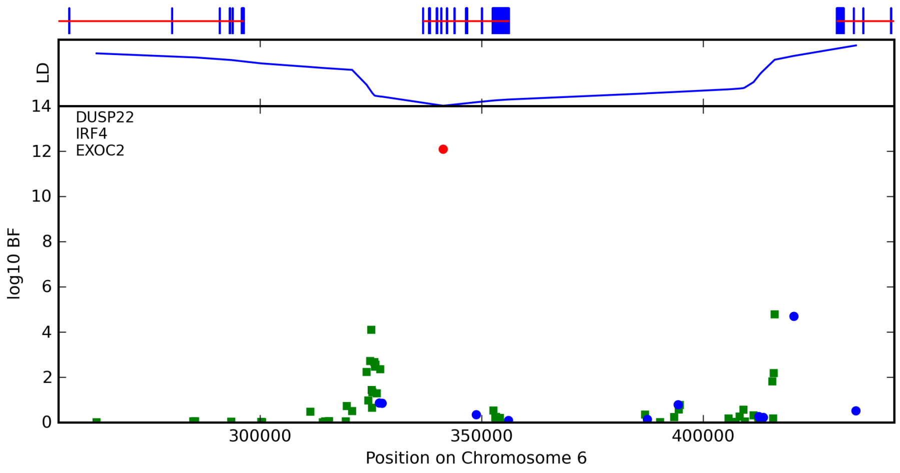

(c) Bayes factors around IRF4

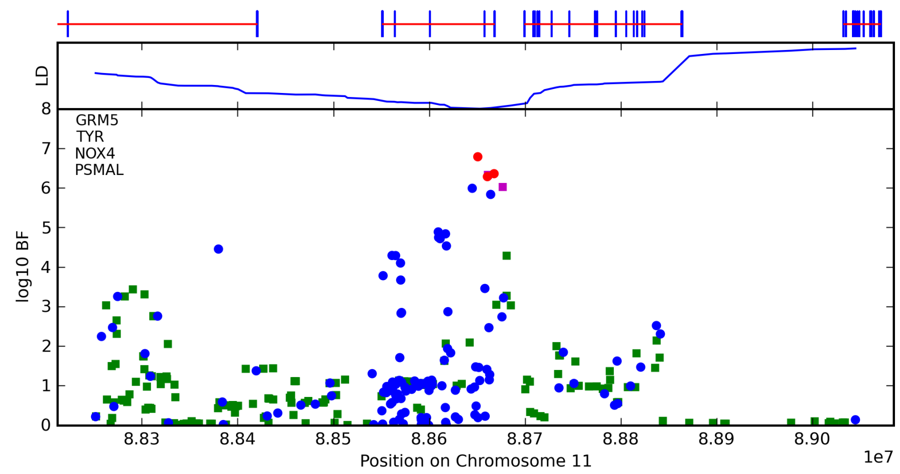

(d) Bayes factors around TYR

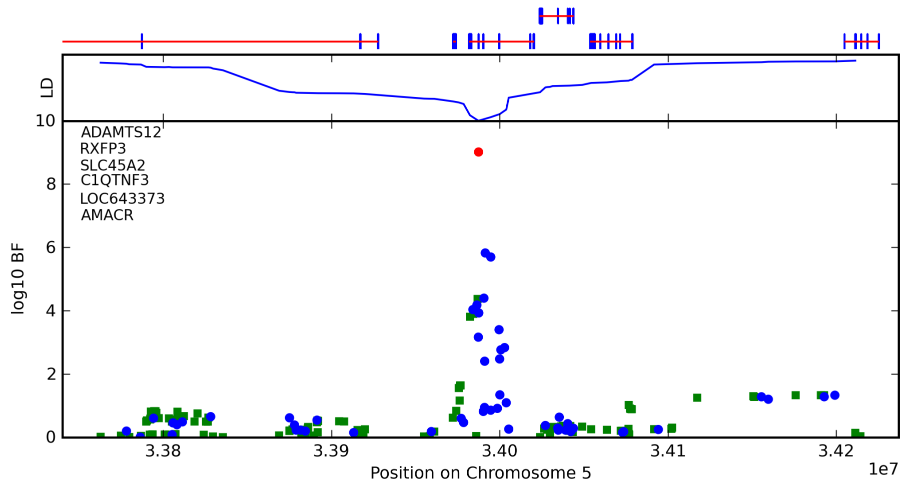

(e) Bayes factors around SLC45A2

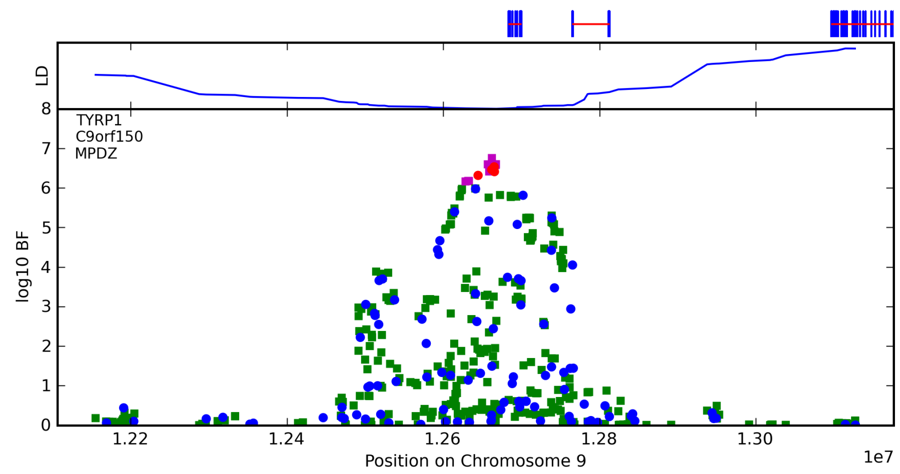

(f) Bayes factors around TYRP1

Figure 5. Region plots for eye color, blue to brown

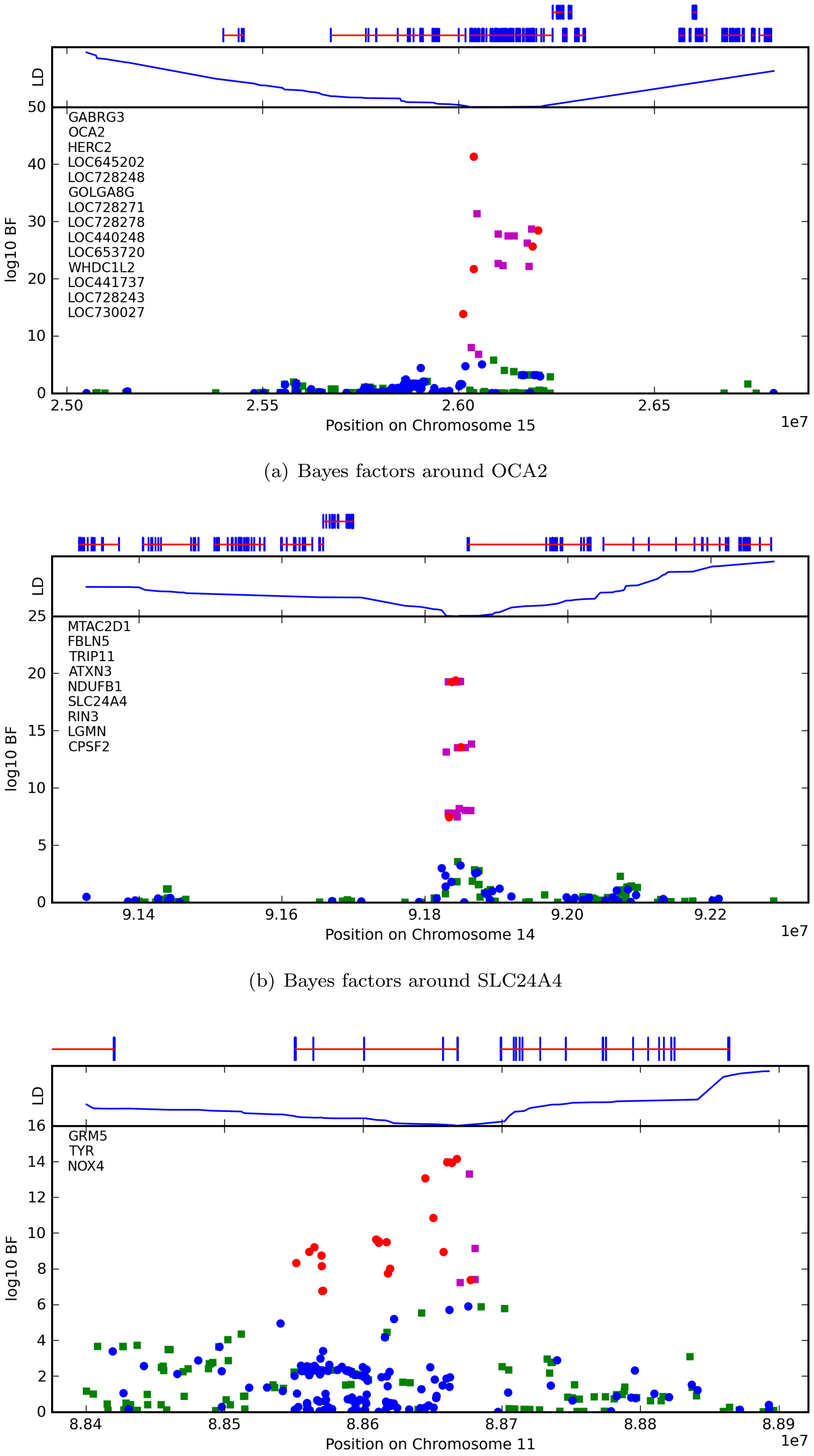

(a) Bayes factors around OCA2

(b) Bayes factors around SLC24A4

(c) Bayes factors around TYR

Figure 6. Region plots for eye color, blue versus green

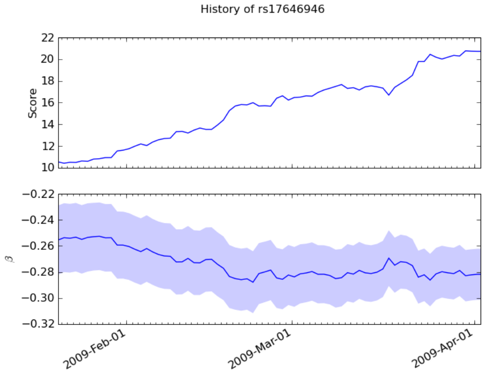

Figure 7. Association over time for rs17646946 and haircurl.

SNP	Chr	Position	Locus	Alleles	MAF	Score	BF	β (95% CI)	OR (95% CI)
rs17646946	1	150,329,391	TCHH	G/A	0.20	41.80	39.90	-0.294 (-0.31 - -0.28)	—
rs3007671	1	150,265,971	NBPF18P S100A11	G/T	0.26	27.22	24.89	-0.215 (-0.24 - -0.19)	—
rs7349332	2	219,464,627	WNT10A	C/T	0.14	13.48	11.01	0.193 (0.15 - 0.24)	—
rs4845779	1	150,745,800	CRNN LCE5A	C/T	0.31	10.03	7.67	0.124 (0.10 - 0.15)	—
rs499697	1	150,759,778	CRCT1 LCE3E	A/G	0.29	9.95	7.61	0.126 (0.10 - 0.16)	—
rs3007684	1	150,301,054	TCHH	T/G	0.34	9.89	7.53	0.120 (0.10 - 0.14)	—
rs6723140	2	219,482,944	WNT10A LOC391485	T/C	0.18	8.79	6.54	0.140 (0.13 - 0.15)	—
rs10177996	2	219,454,805	WNT10A	T/C	0.19	8.45	6.23	0.133 (0.12 - 0.15)	—
rs4672907	2	219,529,413	LOC391485	G/A	0.17	8.24	6.05	0.136 (0.12 - 0.15)	—
rs1556547	6	10,378,363	OFCC1	A/G	0.40	7.81	5.61	-0.101 (-0.12 - -0.08)	—
rs11585118	1	17,478,361	PADI3	C/A	0.16	7.65	5.50	-0.133 (-0.15 - -0.12)	—
rs6587673	1	150,696,776	CRNN LCE5A	A/G	0.43	7.55	5.84	0.100 (0.08 - 0.12)	—
rs1333652	6	10,381,949	OFCC1	C/T	0.40	7.54	5.38	-0.100 (-0.12 - -0.08)	—
rs1556548	6	10,378,372	OFCC1	C/T	0.40	7.54	5.38	-0.100 (-0.12 - -0.08)	—
rs4240887	1	150,797,134	CRCT1 LCE3E	G/A	0.37	7.50	5.50	0.100 (0.08 - 0.12)	—
rs6700998	1	150,700,633	CRNN LCE5A	G/T	0.43	7.48	5.75	0.099 (0.08 - 0.12)	—
rs1333657	6	10,404,322	OFCC1	C/T	0.37	7.42	5.39	-0.100 (-0.12 - -0.08)	—
rs12116609	1	150,764,309	CRCT1 LCE3E	T/C	0.37	7.38	5.26	0.099 (0.08 - 0.12)	—
rs3753451	1	150,751,851	LCE5A CRCT1	G/T	0.37	7.13	5.03	0.097 (0.07 - 0.12)	—
rs1053590	1	150,755,052	CRCT1	C/T	0.37	7.09	5.00	0.097 (0.07 - 0.12)	—
rs2105117	1	150,750,753	LCE5A	A/G	0.38	7.01	4.97	0.097 (0.07 - 0.12)	—

Table 3. All SNPs with scores over 7 for hair curl.

SNP	Chr	Position	Locus	Alleles	MAF	Score	BF	β (95% CI)	OR (95% CI)
rs4481887	1	246,563,486	OR2M7	G/A	0.26	23.18	21.74	-0.514 (-0.62 - -0.41)	0.598 (0.54 - 0.66)
rs4309013	1	246,547,391	OR2M7	T/C	0.27	22.27	20.78	-0.496 (-0.60 - -0.40)	0.609 (0.55 - 0.67)
rs4244187	1	246,560,134	OR2M7	C/T	0.27	22.02	20.44	-0.493 (-0.59 - -0.39)	0.611 (0.55 - 0.68)
rs7555310	1	246,554,261	OR2M7	G/A	0.27	21.72	20.18	-0.489 (-0.59 - -0.39)	0.613 (0.55 - 0.68)
rs6587440	1	246,482,285	OR2M7	G/A	0.27	19.85	18.36	-0.463 (-0.56 - -0.36)	0.629 (0.57 - 0.70)
rs7550630	1	246,428,934	OR2M7	T/C	0.23	14.69	12.29	-0.415 (-0.52 - -0.31)	0.660 (0.59 - 0.73)
rs4916113	1	246,443,915	OR2M7	T/C	0.23	14.51	12.18	-0.409 (-0.51 - -0.31)	0.664 (0.60 - 0.74)
rs7349159	1	246,452,865	OR2M7	T/C	0.25	13.22	11.15	-0.378 (-0.48 - -0.28)	0.686 (0.62 - 0.76)
rs4642918	1	246,477,680	OR2M7	T/G	0.25	13.12	11.15	-0.377 (-0.48 - -0.28)	0.686 (0.62 - 0.76)
rs10788759	1	246,417,399	OR2M7	G/A	0.35	8.75	6.63	-0.274 (-0.36 - -0.18)	0.760 (0.69 - 0.83)
rs9435891	1	246,411,221	OR2M7	C/T	0.35	8.71	6.60	-0.274 (-0.36 - -0.18)	0.760 (0.69 - 0.83)
rs1544175	1	246,590,885	OR2M7	A/G	0.47	8.10	5.99	-0.257 (-0.34 - -0.17)	0.773 (0.71 - 0.84)
rs4559542	1	246,556,603	OR2M7	T/C	0.46	7.75	6.96	-0.244 (-0.33 - -0.16)	0.783 (0.72 - 0.85)

Table 4. All SNPs with scores over 7 for asparagus anosmia.

SNP	Chr	Position	Locus	Alleles	MAF	Score	BF	β (95% CI)	OR (95% CI)
rs10427255	2	145,841,993	2q22.3	T/C	0.46	10.93	8.65	0.280 (0.20 - 0.36)	1.323 (1.22 - 1.43)
rs4422110	2	145,831,368	2q22.3	T/C	0.47	10.58	8.24	0.275 (0.19 - 0.36)	1.316 (1.21 - 1.43)
rs6721116	2	145,872,667	2q22.3	G/A	0.48	9.79	7.78	0.264 (0.18 - 0.34)	1.302 (1.20 - 1.41)
rs1040173	2	145,836,981	2q22.3	C/T	0.46	8.54	6.18	-0.246 (-0.33 - -0.16)	0.782 (0.72 - 0.85)
rs1006134	2	145,852,089	2q22.3	A/G	0.45	8.42	6.07	-0.245 (-0.33 - -0.16)	0.783 (0.72 - 0.85)
rs17410058	2	145,851,244	2q22.3	T/C	0.31	7.99	5.78	0.252 (0.17 - 0.34)	1.286 (1.18 - 1.40)
rs11856995	15	94,126,647	NR2F2	T/C	0.30	7.13	5.71	-0.244 (-0.33 - -0.15)	0.784 (0.72 - 0.86)

Table 5. All SNPs with scores over 7 for photic sneeze reflex.

SNP	Chr	Position	Locus	Alleles	MAF	Score	BF	β (95% CI)	OR (95% CI)
rs12203592	6	341,321	IRF4	C/T	0.18	90.68	87.04	1.611 (1.48 - 1.74)	—
rs12931267	16	88,346,233	MC1R	C/G	0.08	61.08	60.89	1.875 (1.67 - 2.08)	—
rs258322	16	88,283,404	MC1R	G/A	0.10	44.31	44.28	1.446 (1.40 - 1.49)	—
rs4785763	16	88,594,437	MC1R	C/A	0.33	34.32	31.17	0.836 (0.78 - 0.89)	—
rs8049897	16	88,551,703	MC1R	G/A	0.15	29.79	27.76	1.033 (0.98 - 1.08)	—
rs4238833	16	88,578,190	MC1R	T/G	0.37	26.90	24.28	0.719 (0.66 - 0.78)	—
rs1805008	16	88,513,645	MC1R	C/T	0.07	23.18	21.05	1.212 (1.00 - 1.43)	—
rs4408545	16	88,571,529	MC1R	C/T	0.50	20.20	17.48	-0.596 (-0.67 - -0.53)	—
rs164741	16	88,219,799	MC1R	G/A	0.30	20.13	17.38	0.641 (0.58 - 0.70)	—
rs12210050	6	420,489	IRF4	C/T	0.18	18.58	15.87	0.712 (0.58 - 0.84)	—
rs1805009	16	88,514,047	MC1R	G/C	0.02	17.32	14.41	2.017 (1.97 - 2.06)	—
rs872071	6	356,064	IRF4	A/G	0.49	14.38	12.52	0.508 (0.44 - 0.58)	—
rs2353033	16	87,913,062	MC1R	T/C	0.43	13.41	11.09	0.481 (0.42 - 0.55)	—
rs619865	20	33,331,111	ASIP	G/A	0.10	13.26	10.78	0.771 (0.72 - 0.82)	—
rs1540771	6	411,033	IRF4	C/T	0.47	13.24	10.79	0.479 (0.41 - 0.55)	—
rs9378805	6	362,727	IRF4	A/C	0.49	12.91	10.79	0.481 (0.41 - 0.55)	—
rs11547464	16	88,513,592	MC1R	G/A	0.01	12.85	9.91	2.430 (2.38 - 2.48)	—
rs3778607	6	348,799	IRF4	G/A	0.47	12.67	10.65	-0.471 (-0.54 - -0.40)	—
rs7188458	16	88,253,985	MC1R	G/A	0.44	12.33	10.49	0.465 (0.40 - 0.53)	—
rs291671	20	31,414,506	ASIP	A/G	0.10	12.04	9.89	0.772 (0.58 - 0.96)	—
rs4959270	6	402,748	IRF4	C/A	0.50	11.93	9.53	0.451 (0.38 - 0.52)	—
rs352935	16	88,176,081	MC1R	T/C	0.48	11.71	9.37	0.450 (0.38 - 0.52)	—
rs7196459	16	88,668,978	MC1R	G/T	0.08	11.59	9.21	0.846 (0.63 - 1.07)	—
rs11861084	16	88,403,211	MC1R	C/A	0.41	11.27	8.90	-0.445 (-0.51 - -0.38)	—
rs2241039	16	88,615,938	MC1R	G/A	0.38	10.98	8.59	-0.456 (-0.52 - -0.39)	—
rs7204478	16	88,322,986	MC1R	C/T	0.45	10.61	8.40	0.427 (0.35 - 0.50)	—
rs6088316	20	31,890,503	ASIP	A/G	0.17	10.40	8.37	0.568 (0.43 - 0.71)	—
rs4911442	20	32,818,707	ASIP	A/G	0.12	10.24	7.98	0.629 (0.46 - 0.79)	—
rs464349	16	88,183,752	MC1R	C/T	0.48	10.20	7.89	-0.412 (-0.49 - -0.34)	—
rs7195066	16	88,363,824	MC1R	C/T	0.32	10.12	7.76	-0.449 (-0.55 - -0.35)	—
rs761238	20	31,983,649	ASIP	T/G	0.33	9.90	7.56	0.428 (0.37 - 0.49)	—
rs1800286	16	88,397,262	MC1R	C/T	0.41	9.85	7.62	-0.413 (-0.50 - -0.33)	—
rs17305657	20	31,270,249	ASIP	T/C	0.09	9.82	7.56	0.697 (0.65 - 0.74)	—
rs4812405	20	34,709,999	ASIP	C/A	0.07	9.70	7.40	0.783 (0.74 - 0.83)	—
rs1474976	20	34,195,786	ASIP	A/C	0.11	9.50	7.23	0.620 (0.45 - 0.79)	—
rs2153271	9	16,854,521	BNC2	T/C	0.41	9.40	7.28	-0.402 (-0.47 - -0.34)	—
rs9328192	6	379,364	IRF4	A/G	0.50	9.39	7.11	0.395 (0.32 - 0.47)	—
rs2284378	20	32,051,756	ASIP	C/T	0.33	9.22	6.93	0.415 (0.32 - 0.51)	—
rs6060612	20	33,853,941	ASIP	G/A	0.10	9.20	6.95	0.640 (0.59 - 0.69)	—
rs2268089	20	32,130,959	ASIP	C/T	0.33	9.08	6.79	0.411 (0.32 - 0.50)	—
rs1800359	16	88,332,762	MC1R	G/A	0.41	9.04	6.87	-0.394 (-0.46 - -0.33)	—
rs11648785	16	88,612,062	MC1R	C/T	0.31	8.96	6.70	-0.424 (-0.52 - -0.33)	—
rs4911379	20	31,998,966	ASIP	C/A	0.33	8.93	6.67	0.406 (0.35 - 0.46)	—
rs8060934	16	88,447,526	MC1R	T/C	0.48	8.86	6.60	-0.390 (-0.46 - -0.32)	—
rs9405675	6	389,600	IRF4	A/G	0.36	8.72	6.86	-0.392 (-0.48 - -0.30)	—
rs154659	16	88,194,838	MC1R	T/C	0.25	8.62	6.45	0.433 (0.38 - 0.49)	—
rs463701	16	88,126,261	MC1R	A/G	0.45	8.53	6.41	-0.380 (-0.46 - -0.30)	—
rs4911414	20	32,193,105	ASIP	G/T	0.34	8.52	6.26	0.393 (0.30 - 0.48)	—
rs12599531	16	88,436,902	MC1R	C/T	0.37	8.32	6.15	-0.387 (-0.47 - -0.30)	—
rs382745	16	88,131,087	MC1R	A/G	0.44	8.28	6.16	-0.372 (-0.45 - -0.29)	—
rs17396317	20	31,254,038	ASIP	G/A	0.12	7.99	5.80	0.544 (0.49 - 0.59)	—
rs950286	6	374,457	IRF4	G/A	0.10	7.40	5.30	-0.558 (-0.61 - -0.51)	—
rs1339548	9	16,836,323	BNC2	T/C	0.33	7.39	5.66	-0.367 (-0.43 - -0.31)	—
rs9405681	6	394,358	IRF4	C/T	0.23	7.34	5.21	-0.406 (-0.52 - -0.29)	—
rs2268086	20	32,112,399	ASIP	G/A	0.39	7.25	5.07	0.349 (0.29 - 0.41)	—
rs1042602	11	88,551,344	TYR	C/A	0.37	7.23	5.48	-0.355 (-0.42 - -0.29)	—
rs4911145	20	32,078,884	ASIP	G/A	0.39	7.22	5.05	0.348 (0.28 - 0.41)	—
rs2424994	20	32,596,578	ASIP	C/T	0.17	7.22	5.32	0.461 (0.32 - 0.60)	—
rs9503644	6	360,406	IRF4	G/A	0.14	7.11	4.99	-0.484 (-0.53 - -0.43)	—
rs6060891	20	34,338,559	ASIP	C/T	0.28	7.01	4.98	0.372 (0.27 - 0.47)	—

Table 6. All SNPs with scores over 7 for freckling.

SNP	Chr	Position	Locus	Alleles	MAF	Score	BF	β (95% CI)	OR (95% CI)
rs12913832	15	26,039,213	OCA2/HERC2	G/A	0.23	87.07	85.76	0.974 (0.94 - 1.01)	—
rs1667394	15	26,203,777	OCA2/HERC2	T/C	0.16	48.55	45.83	0.854 (0.82 - 0.89)	—
rs8039195	15	26,189,679	OCA2/HERC2	T/C	0.14	40.89	38.40	0.834 (0.80 - 0.87)	—
rs4778241	15	26,012,308	OCA2/HERC2	C/A	0.17	29.87	27.09	0.638 (0.60 - 0.67)	—
rs12203592	6	341,321	IRF4	C/T	0.18	27.64	27.17	0.589 (0.50 - 0.68)	—
rs7183877	15	26,039,328	OCA2/HERC2	C/A	0.08	21.15	18.47	0.759 (0.73 - 0.79)	—
rs16891982	5	33,987,450	SLC45A2	G/C	0.03	19.41	16.76	1.104 (1.07 - 1.13)	—
rs16950987	15	26,199,823	OCA2/HERC2	G/A	0.05	15.41	12.81	0.764 (0.73 - 0.80)	—
rs7495174	15	26,017,833	OCA2/HERC2	A/G	0.06	15.20	12.56	0.711 (0.55 - 0.87)	—
rs8028689	15	26,162,483	OCA2/HERC2	T/C	0.05	14.87	12.28	0.753 (0.72 - 0.78)	—
rs35391	5	33,991,430	SLC45A2	C/T	0.02	14.62	11.84	1.128 (0.86 - 1.40)	—
rs11636232	15	26,060,221	OCA2/HERC2	C/T	0.40	14.53	12.04	-0.340 (-0.40 - -0.28)	—
rs4778138	15	26,009,415	OCA2/HERC2	A/G	0.12	14.40	11.83	0.510 (0.40 - 0.62)	—
rs16950979	15	26,194,101	OCA2/HERC2	A/G	0.05	14.25	11.69	0.743 (0.57 - 0.92)	—
rs2240204	15	26,167,627	OCA2/HERC2	G/A	0.05	14.25	11.69	0.743 (0.71 - 0.77)	—
rs35390	5	33,991,083	SLC45A2	A/C	0.02	13.78	11.05	1.104 (0.83 - 1.38)	—
rs12896399	14	91,843,416	SLC24A4	G/T	0.44	12.34	9.83	-0.308 (-0.36 - -0.26)	—
rs4904866	14	91,838,256	SLC24A4	C/T	0.44	12.15	9.65	-0.306 (-0.36 - -0.25)	—
rs35408	5	34,000,695	SLC45A2	T/C	0.02	11.19	8.63	0.964 (0.93 - 0.99)	—
rs35412	5	34,002,902	SLC45A2	G/C	0.02	11.19	8.63	0.964 (0.93 - 0.99)	—
rs7174027	15	26,002,360	OCA2/HERC2	G/A	0.09	10.07	7.72	0.474 (0.44 - 0.51)	—
rs35394	5	33,984,076	SLC45A2	T/C	0.02	9.85	7.32	1.027 (1.00 - 1.06)	—
rs35398	5	33,987,583	SLC45A2	G/T	0.02	9.85	7.32	1.027 (0.72 - 1.33)	—
rs40132	5	33,986,460	SLC45A2	A/G	0.02	9.65	7.13	1.020 (0.71 - 1.33)	—
rs12931267	16	88,346,233	MC1R	C/G	0.08	9.48	7.33	-0.556 (-0.72 - -0.39)	—
rs4778211	15	25,872,900	OCA2/HERC2	C/A	0.16	8.14	5.95	0.342 (0.31 - 0.38)	—
rs12210050	6	420,489	IRF4	C/T	0.18	8.14	5.95	0.309 (0.22 - 0.39)	—
rs4904868	14	91,850,754	SLC24A4	C/T	0.45	7.89	5.68	0.245 (0.19 - 0.30)	—
rs26722	5	33,999,627	SLC45A2	C/T	0.02	7.75	5.41	0.873 (0.58 - 1.17)	—
rs17128162	14	91,850,110	SLC24A4	G/A	0.11	7.51	5.44	-0.378 (-0.41 - -0.35)	—
rs4904864	14	91,834,272	SLC24A4	G/A	0.35	7.48	5.32	0.250 (0.21 - 0.29)	—
rs6918152	6	487,159	LOC730077	G/A	0.38	7.07	4.91	-0.240 (-0.28 - -0.20)	—
rs9392056	6	463,078	EXOC2	A/G	0.38	7.03	4.87	-0.238 (-0.30 - -0.18)	—

Table 7. All SNPs with scores over 7 for haircolor.

SNP	Chr	Position	Locus	Alleles	MAF	Score	BF	β (95% CI)	OR (95% CI)
rs12931267	16	88,346,233	MC1R	C/G	0.08	86.28	89.80	0.556 (0.51 - 0.61)	—
rs258322	16	88,283,404	MC1R	G/A	0.10	56.77	57.53	0.410 (0.40 - 0.42)	—
rs4785763	16	88,594,437	MC1R	C/A	0.33	49.68	48.61	0.252 (0.24 - 0.27)	—
rs4238833	16	88,578,190	MC1R	T/G	0.37	42.77	41.02	0.228 (0.21 - 0.24)	—
rs8049897	16	88,551,703	MC1R	G/A	0.15	42.38	40.65	0.309 (0.30 - 0.32)	—
rs1805008	16	88,513,645	MC1R	C/T	0.07	37.48	36.19	0.387 (0.33 - 0.44)	—
rs164741	16	88,219,799	MC1R	G/A	0.30	34.29	32.12	0.211 (0.20 - 0.23)	—
rs4408545	16	88,571,529	MC1R	C/T	0.50	24.23	22.14	-0.164 (-0.18 - -0.15)	—
rs7204478	16	88,322,986	MC1R	C/T	0.45	21.90	20.79	0.157 (0.14 - 0.18)	—
rs7188458	16	88,253,985	MC1R	G/A	0.44	20.14	17.91	0.151 (0.13 - 0.17)	—
rs11861084	16	88,403,211	MC1R	C/A	0.41	19.09	16.35	-0.147 (-0.16 - -0.13)	—
rs1800286	16	88,397,262	MC1R	C/T	0.41	18.63	15.96	-0.145 (-0.17 - -0.12)	—
rs1800359	16	88,332,762	MC1R	G/A	0.41	17.90	15.21	-0.142 (-0.16 - -0.13)	—
rs1805009	16	88,514,047	MC1R	G/C	0.02	17.43	14.48	0.508 (0.50 - 0.52)	—
rs352935	16	88,176,081	MC1R	T/C	0.48	17.37	15.66	0.139 (0.12 - 0.16)	—
rs8060934	16	88,447,526	MC1R	T/C	0.48	16.24	14.43	-0.135 (-0.15 - -0.12)	—
rs2241039	16	88,615,938	MC1R	G/A	0.38	15.39	12.92	-0.137 (-0.15 - -0.12)	—
rs291671	20	31,414,506	ASIP	A/G	0.10	14.70	12.45	0.215 (0.17 - 0.26)	—
rs12599531	16	88,436,902	MC1R	C/T	0.37	14.15	11.76	-0.129 (-0.15 - -0.11)	—
rs2353033	16	87,913,062	MC1R	T/C	0.43	13.93	11.55	0.123 (0.11 - 0.14)	—
rs2159116	16	88,359,011	MC1R	C/A	0.17	12.58	10.37	0.157 (0.14 - 0.17)	—
rs8058895	16	88,342,308	MC1R	T/C	0.17	12.56	10.38	0.157 (0.14 - 0.17)	—
rs17305657	20	31,270,249	ASIP	T/C	0.09	12.31	9.89	0.197 (0.19 - 0.21)	—
rs460879	16	88,240,390	MC1R	C/T	0.46	11.77	9.38	-0.113 (-0.13 - -0.09)	—
rs7196459	16	88,668,978	MC1R	G/T	0.08	11.31	8.94	0.208 (0.15 - 0.26)	—
rs7195066	16	88,363,824	MC1R	C/T	0.32	11.10	8.69	-0.118 (-0.14 - -0.09)	—
rs459920	16	88,258,328	MC1R	T/C	0.45	10.77	8.35	-0.108 (-0.12 - -0.09)	—
rs464349	16	88,183,752	MC1R	C/T	0.48	10.75	8.32	-0.106 (-0.12 - -0.09)	—
rs619865	20	33,331,111	ASIP	G/A	0.10	9.89	7.59	0.165 (0.15 - 0.18)	—
rs9936896	16	88,596,560	MC1R	T/C	0.20	9.87	7.59	0.129 (0.12 - 0.14)	—
rs447735	16	88,261,850	MC1R	T/C	0.44	9.85	7.49	-0.103 (-0.12 - -0.09)	—
rs2306633	16	87,882,779	MC1R	G/A	0.26	9.10	6.84	-0.112 (-0.13 - -0.10)	—
rs17396317	20	31,254,038	ASIP	G/A	0.12	9.00	6.90	0.145 (0.13 - 0.16)	—
rs6500437	16	88,317,399	MC1R	T/C	0.30	8.58	6.71	0.105 (0.09 - 0.12)	—
rs11648785	16	88,612,062	MC1R	C/T	0.31	8.47	6.21	-0.103 (-0.13 - -0.08)	—
rs4347628	16	88,098,136	MC1R	C/T	0.44	8.42	6.28	-0.095 (-0.11 - -0.07)	—
rs2016571	16	88,371,777	MC1R	G/C	0.31	8.20	6.15	0.102 (0.09 - 0.12)	—
rs463701	16	88,126,261	MC1R	A/G	0.45	8.07	6.02	-0.092 (-0.11 - -0.07)	—
rs1061646	16	88,333,478	MC1R	G/A	0.31	8.04	6.13	0.100 (0.09 - 0.11)	—
rs3785275	16	88,369,530	MC1R	C/G	0.31	7.87	5.88	0.099 (0.07 - 0.12)	—
rs382745	16	88,131,087	MC1R	A/G	0.44	7.86	5.82	-0.091 (-0.11 - -0.07)	—
rs16966142	16	88,378,534	MC1R	C/T	0.09	7.85	5.71	-0.168 (-0.22 - -0.11)	—
rs2378249	20	32,681,751	ASIP	A/G	0.16	7.77	5.62	0.121 (0.09 - 0.16)	—
rs2378199	20	32,650,141	ASIP	C/T	0.16	7.71	5.56	0.121 (0.09 - 0.16)	—
rs4812405	20	34,709,999	ASIP	C/A	0.07	7.42	5.47	0.170 (0.16 - 0.18)	—
rs7189734	16	88,328,051	MC1R	G/A	0.25	7.39	5.36	0.101 (0.09 - 0.12)	—
rs1800345	16	88,342,718	MC1R	C/T	0.31	7.34	5.30	0.096 (0.07 - 0.12)	—
rs4911442	20	32,818,707	ASIP	A/G	0.12	7.23	5.27	0.131 (0.09 - 0.17)	—
rs7361656	20	33,192,808	ASIP	T/G	0.24	7.11	5.52	0.099 (0.09 - 0.11)	—
rs6500452	16	88,386,006	MC1R	T/C	0.31	7.02	4.96	0.094 (0.08 - 0.11)	—

Table 8. All SNPs with scores over 7 for red hair.

SNP	Chr	Position	Locus	Alleles	MAF	Score	BF	β (95% CI)	OR (95% CI)
rs12913832	15	26,039,213	OCA2/HERC2	G/A	0.23	> 300	> 300	2.491 (2.47 - 2.51)	—
rs1667394	15	26,203,777	OCA2/HERC2	T/C	0.16	> 300	> 300	2.176 (2.15 - 2.20)	—
rs8039195	15	26,189,679	OCA2/HERC2	T/C	0.14	291.35	293.35	2.088 (2.06 - 2.12)	—
rs4778241	15	26,012,308	OCA2/HERC2	C/A	0.17	232.34	230.88	1.692 (1.66 - 1.72)	—
rs4778138	15	26,009,415	OCA2/HERC2	A/G	0.12	135.65	131.98	1.529 (1.43 - 1.63)	—
rs16950987	15	26,199,823	OCA2/HERC2	G/A	0.05	125.44	121.47	2.169 (2.14 - 2.20)	—
rs7495174	15	26,017,833	OCA2/HERC2	A/G	0.06	124.09	120.23	2.032 (1.88 - 2.18)	—
rs8028689	15	26,162,483	OCA2/HERC2	T/C	0.05	122.42	118.46	2.156 (2.13 - 2.18)	—
rs7183877	15	26,039,328	OCA2/HERC2	C/A	0.08	120.28	116.87	1.759 (1.73 - 1.79)	—
rs2240204	15	26,167,627	OCA2/HERC2	G/A	0.05	117.51	113.57	2.127 (2.10 - 2.16)	—
rs16950979	15	26,194,101	OCA2/HERC2	A/G	0.05	117.42	113.48	2.127 (1.96 - 2.29)	—
rs11636232	15	26,060,221	OCA2/HERC2	C/T	0.40	115.29	113.62	-0.936 (-0.99 - -0.89)	—
rs7174027	15	26,002,360	OCA2/HERC2	G/A	0.09	96.16	92.37	1.471 (1.44 - 1.50)	—
rs6497253	15	25,962,144	OCA2/HERC2	G/A	0.21	43.47	40.32	0.710 (0.68 - 0.74)	—
rs1470608	15	25,961,716	OCA2/HERC2	G/T	0.14	42.49	39.33	0.839 (0.74 - 0.94)	—
rs7170869	15	25,962,343	OCA2/HERC2	A/G	0.14	42.01	38.89	0.836 (0.73 - 0.94)	—
rs4778232	15	25,955,360	OCA2/HERC2	C/T	0.21	38.75	35.90	0.663 (0.58 - 0.74)	—
rs749846	15	25,942,585	OCA2/HERC2	C/A	0.14	34.53	31.70	0.772 (0.74 - 0.81)	—
rs1448485	15	25,956,336	OCA2/HERC2	G/T	0.12	33.58	30.51	0.785 (0.67 - 0.90)	—
rs1597196	15	25,968,517	OCA2/HERC2	G/T	0.17	31.88	28.81	0.648 (0.56 - 0.74)	—
rs8024968	15	25,957,284	OCA2/HERC2	C/T	0.10	30.02	27.01	0.802 (0.68 - 0.92)	—
rs16950821	15	25,957,102	OCA2/HERC2	G/A	0.10	29.69	26.69	0.797 (0.77 - 0.83)	—
rs7179994	15	25,997,365	OCA2/HERC2	A/G	0.13	28.05	25.07	0.669 (0.57 - 0.77)	—
rs4778211	15	25,872,900	OCA2/HERC2	C/A	0.16	27.02	24.36	0.628 (0.60 - 0.66)	—
rs7170451	15	25,865,819	OCA2/HERC2	G/A	0.27	26.48	23.52	0.503 (0.47 - 0.54)	—
rs1800407	15	25,903,913	OCA2/HERC2	C/T	0.08	23.49	20.78	0.823 (0.68 - 0.97)	—
rs3794604	15	25,945,660	OCA2/HERC2	C/T	0.10	23.37	20.52	0.699 (0.58 - 0.82)	—
rs1448488	15	25,890,452	OCA2/HERC2	T/C	0.28	23.16	20.33	0.465 (0.43 - 0.50)	—
rs1800411	15	25,885,516	OCA2/HERC2	A/G	0.28	22.98	20.15	0.464 (0.40 - 0.53)	—
rs12910433	15	25,902,239	OCA2/HERC2	A/G	0.29	22.40	19.61	0.456 (0.39 - 0.52)	—
rs1900758	15	25,903,692	OCA2/HERC2	T/C	0.29	22.29	19.54	0.464 (0.43 - 0.50)	—
rs4778220	15	25,894,733	OCA2/HERC2	A/G	0.17	20.58	17.83	0.531 (0.44 - 0.62)	—
rs728405	15	25,873,448	OCA2/HERC2	A/C	0.22	19.91	17.34	0.466 (0.39 - 0.54)	—
rs1037208	15	25,904,952	OCA2/HERC2	T/G	0.17	19.90	17.16	0.516 (0.48 - 0.55)	—
rs11635884	15	26,042,564	OCA2/HERC2	T/C	0.01	19.30	15.10	2.635 (2.61 - 2.66)	—
rs2594935	15	25,858,633	OCA2/HERC2	G/A	0.29	19.23	16.47	0.417 (0.38 - 0.45)	—
rs2703952	15	25,855,576	OCA2/HERC2	T/G	0.16	17.06	14.68	0.495 (0.46 - 0.53)	—
rs12896399	14	91,843,416	SLC24A4	G/T	0.44	15.95	13.49	-0.343 (-0.39 - -0.29)	—
rs4904866	14	91,838,256	SLC24A4	C/T	0.44	15.81	13.35	-0.342 (-0.39 - -0.29)	—
rs12203592	6	341,321	IRF4	C/T	0.18	14.69	13.26	-0.424 (-0.51 - -0.34)	—
rs1800404	15	25,909,368	OCA2/HERC2	T/C	0.21	14.47	11.91	0.411 (0.38 - 0.44)	—
rs7178315	15	25,911,504	OCA2/HERC2	C/T	0.21	14.20	11.64	0.407 (0.32 - 0.49)	—
rs977589	15	25,853,198	OCA2/HERC2	C/T	0.47	13.76	11.19	-0.322 (-0.37 - -0.27)	—
rs4904868	14	91,850,754	SLC24A4	C/T	0.45	13.49	10.95	0.317 (0.27 - 0.37)	—
rs2871875	15	25,938,449	OCA2/HERC2	G/A	0.24	12.57	10.14	0.361 (0.33 - 0.40)	—
rs16891982	5	33,987,450	SLC45A2	G/C	0.03	11.83	9.42	0.840 (0.81 - 0.87)	—
rs1107267	15	25,645,234	OCA2/HERC2	G/A	0.26	11.76	10.33	0.338 (0.30 - 0.37)	—
rs3930739	15	25,713,937	OCA2/HERC2	C/T	0.46	11.38	8.96	-0.288 (-0.34 - -0.24)	—
rs17565953	15	25,692,250	OCA2/HERC2	G/T	0.40	10.94	8.68	-0.289 (-0.34 - -0.23)	—
rs8036718	15	25,727,152	OCA2/HERC2	G/A	0.38	10.63	8.39	-0.284 (-0.32 - -0.24)	—
rs6497238	15	25,727,373	OCA2/HERC2	T/C	0.38	10.43	8.18	-0.281 (-0.32 - -0.24)	—
rs7496968	15	25,984,569	OCA2/HERC2	A/G	0.11	9.88	8.09	0.430 (0.31 - 0.55)	—
rs11631195	15	25,695,483	OCA2/HERC2	G/A	0.37	9.27	7.05	-0.266 (-0.31 - -0.23)	—
rs4904864	14	91,834,272	SLC24A4	G/A	0.35	9.17	6.90	0.272 (0.23 - 0.31)	—
rs13329466	15	25,950,357	OCA2/HERC2	G/A	0.23	9.10	6.91	-0.301 (-0.34 - -0.27)	—
rs4778192	15	25,753,656	OCA2/HERC2	T/C	0.47	8.66	6.39	-0.248 (-0.29 - -0.20)	—
rs977588	15	25,852,901	OCA2/HERC2	A/C	0.41	8.55	6.48	0.252 (0.20 - 0.31)	—
rs1393350	11	88,650,694	TYR	G/A	0.27	8.49	6.25	-0.278 (-0.32 - -0.24)	—
rs438702	15	26,798,209	OCA2/HERC2	T/C	0.40	8.49	6.22	0.255 (0.21 - 0.30)	—
rs4778189	15	25,745,823	OCA2/HERC2	G/A	0.47	8.43	6.21	-0.245 (-0.29 - -0.20)	—
rs2311469	15	25,742,663	OCA2/HERC2	G/T	0.47	8.35	6.12	-0.243 (-0.29 - -0.19)	—
rs35391	5	33,991,430	SLC45A2	C/T	0.02	8.08	5.78	0.814 (0.54 - 1.08)	—
rs8035334	15	25,753,962	OCA2/HERC2	G/A	0.48	8.02	5.95	-0.239 (-0.29 - -0.19)	—
rs35390	5	33,991,083	SLC45A2	A/C	0.02	7.86	5.58	0.811 (0.54 - 1.08)	—
rs1847134	11	88,644,901	TYR	A/C	0.32	7.57	5.40	-0.249 (-0.31 - -0.19)	—
rs10960751	9	12,665,264	TYRP1	C/T	0.37	7.54	5.35	0.240 (0.18 - 0.30)	—
rs1408799	9	12,662,097	TYRP1	C/T	0.31	7.42	5.39	0.251 (0.19 - 0.32)	—
rs2594909	15	25,824,657	OCA2/HERC2	T/C	0.41	7.27	5.10	-0.231 (-0.27 - -0.19)	—
rs35408	5	34,000,695	SLC45A2	T/C	0.02	7.27	5.06	0.765 (0.73 - 0.80)	—
rs35412	5	34,002,902	SLC45A2	G/C	0.02	7.12	4.92	0.756 (0.73 - 0.79)	—
rs1137134	9	12,702,157	TYRP1 C9orf150	A/G	0.40	7.10	4.94	0.228 (0.17 - 0.28)	—
rs2703936	15	25,831,670	OCA2/HERC2	A/C	0.41	7.06	4.91	-0.228 (-0.28 - -0.17)	—

Table 9. All SNPs with scores over 7 for eye color.

SNP	Chr	Position	Locus	Alleles	MAF	Score	BF	β (95% CI)	OR (95% CI)
rs12913832	15	26,039,213	OCA2/HERC2	G/A	0.23	51.52	41.34	2.131 (1.79 - 2.47)	8.425 (5.99 - 11.84)
rs1667394	15	26,203,777	OCA2/HERC2	T/C	0.16	36.09	28.45	1.944 (1.58 - 2.31)	6.985 (4.85 - 10.06)
rs8039195	15	26,189,679	OCA2/HERC2	T/C	0.14	32.86	25.63	1.910 (1.54 - 2.29)	6.753 (4.64 - 9.83)
rs7183877	15	26,039,328	OCA2/HERC2	C/A	0.08	29.54	21.57	2.275 (1.77 - 2.78)	9.732 (5.86 - 16.16)
rs12896399	14	91,843,416	SLC24A4	G/T	0.44	22.82	19.49	-0.546 (-0.66 - -0.44)	0.579 (0.52 - 0.65)
rs4904866	14	91,838,256	SLC24A4	C/T	0.44	22.72	19.39	-0.545 (-0.65 - -0.44)	0.580 (0.52 - 0.65)
rs4778241	15	26,012,308	OCA2/HERC2	C/A	0.17	17.26	13.85	0.886 (0.68 - 1.10)	2.426 (1.96 - 2.99)
rs4904868	14	91,850,754	SLC24A4	C/T	0.45	16.80	13.61	0.471 (0.36 - 0.58)	1.602 (1.43 - 1.79)
rs1847134	11	88,644,901	TYR	A/C	0.32	14.95	13.13	-0.461 (-0.57 - -0.35)	0.631 (0.56 - 0.71)
rs1393350	11	88,650,694	TYR	G/A	0.27	12.86	10.86	-0.444 (-0.56 - -0.33)	0.642 (0.57 - 0.72)
rs10765198	11	88,609,422	TYR	T/C	0.29	11.55	9.75	-0.414 (-0.53 - -0.30)	0.661 (0.59 - 0.74)
rs1827430	11	88,658,088	TYR	A/G	0.38	10.97	8.93	-0.377 (-0.49 - -0.27)	0.686 (0.61 - 0.77)
rs11018528	11	88,570,025	TYR	A/G	0.30	10.48	8.73	-0.391 (-0.51 - -0.27)	0.676 (0.60 - 0.76)
rs4904864	14	91,834,272	SLC24A4	G/A	0.35	10.07	7.48	0.375 (0.26 - 0.49)	1.455 (1.30 - 1.63)
rs7120151	11	88,380,027	TYR	G/A	0.29	9.61	8.19	-0.375 (-0.49 - -0.26)	0.687 (0.61 - 0.77)
rs1806319	11	88,677,584	TYR	T/C	0.37	9.35	7.37	-0.348 (-0.46 - -0.24)	0.706 (0.63 - 0.79)
rs1800407	15	25,903,913	OCA2/HERC2	C/T	0.08	8.70	5.46	0.743 (0.49 - 0.99)	2.102 (1.63 - 2.70)
rs7495174	15	26,017,833	OCA2/HERC2	A/G	0.06	7.58	4.70	1.303 (0.79 - 1.81)	3.679 (2.21 - 6.12)
rs1391880	11	88,035,044	TYR	A/G	0.30	7.43	6.06	-0.318 (-0.43 - -0.20)	0.728 (0.65 - 0.82)

Table 10. All SNPs with scores over 7 for eye color, green versus blue.

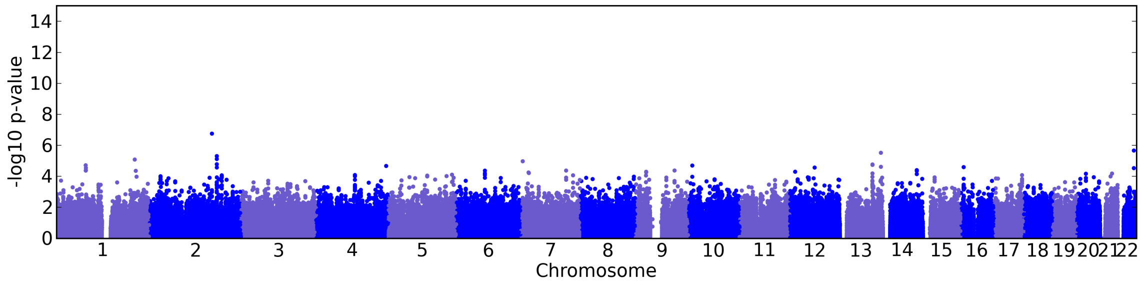

(a) Footedness

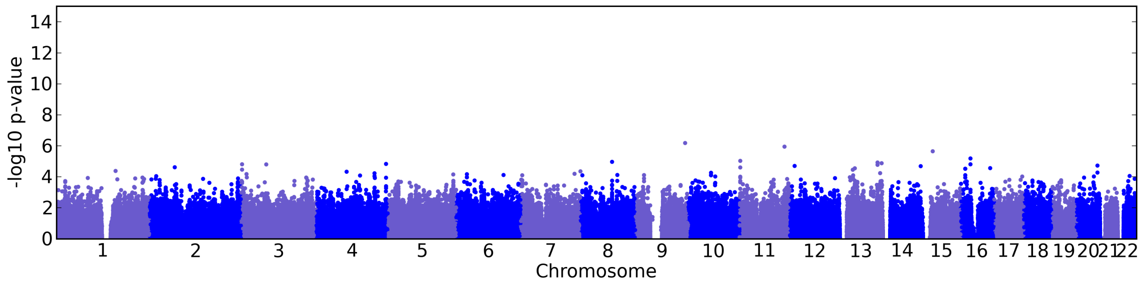

(b) Astigmatism

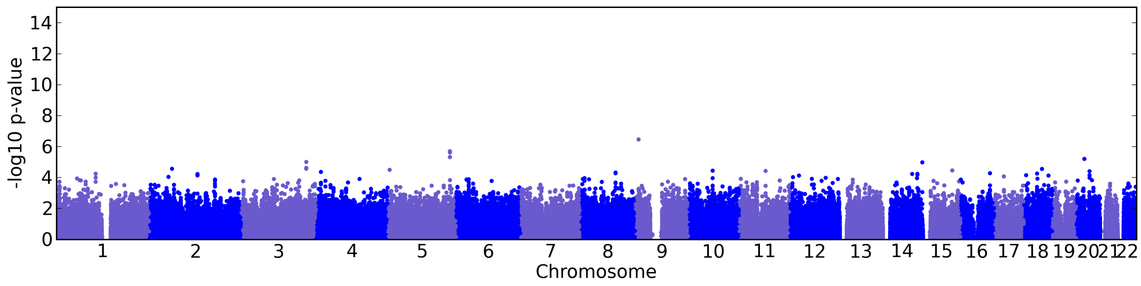

(c) Braces

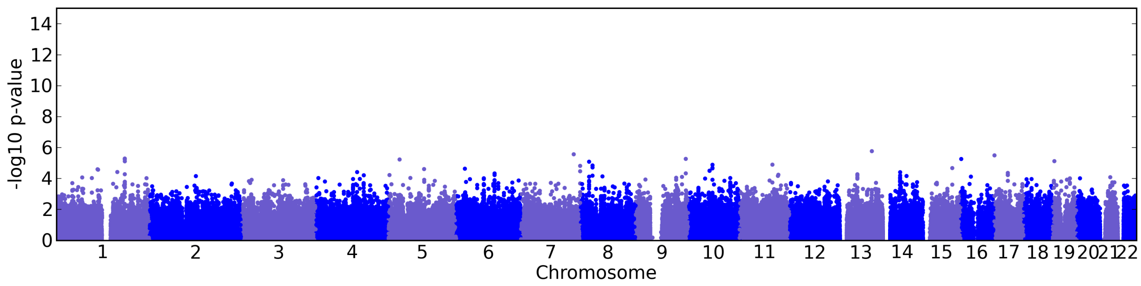

(d) Glasses

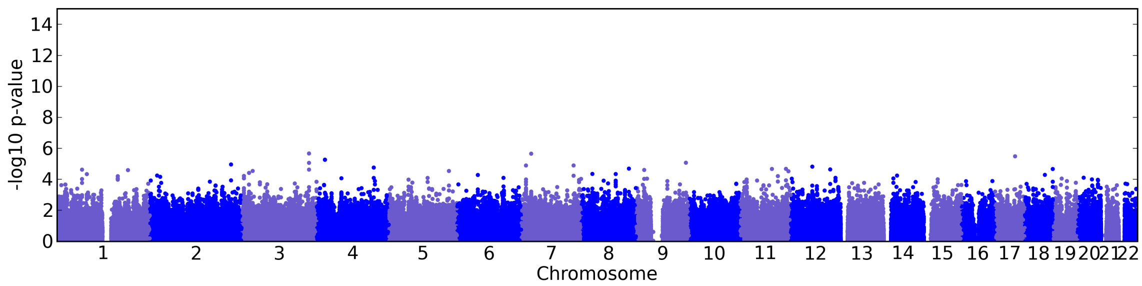

(e) Hand clasp

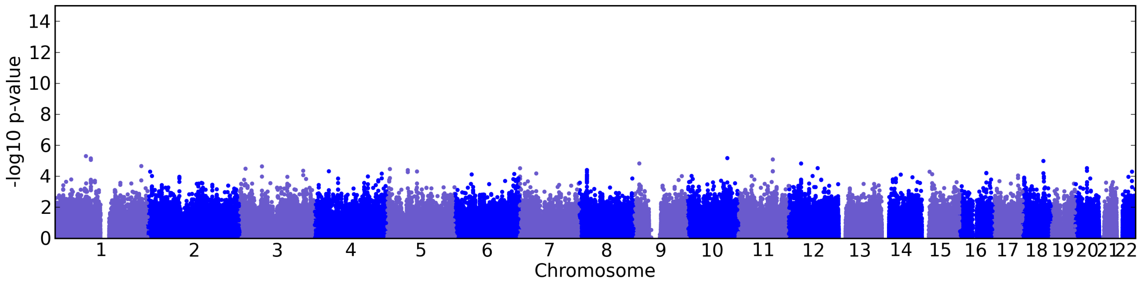

(f) Handedness

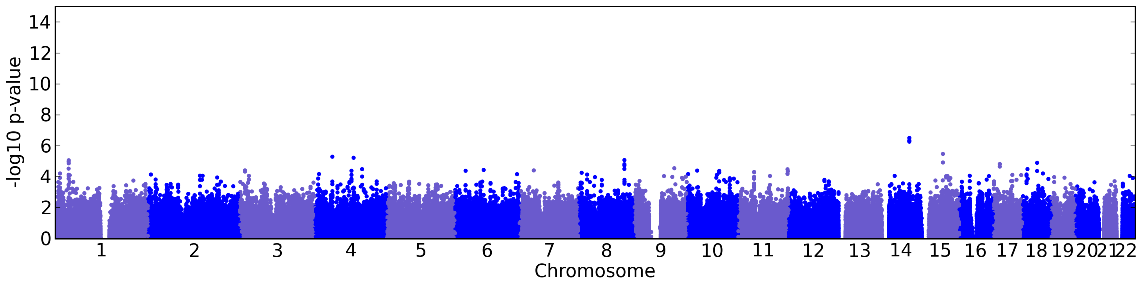

(g) Morningness

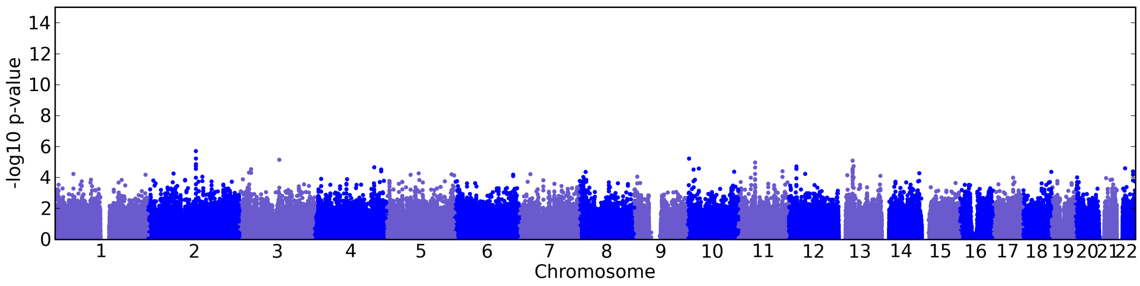

(h) Ocular dominance

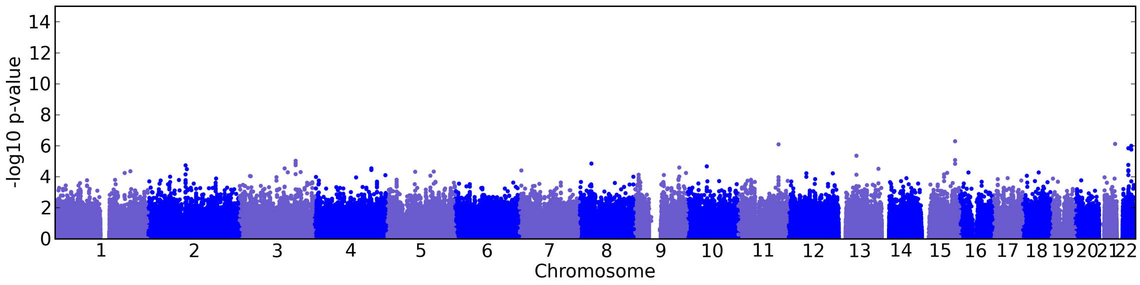

(i) Optimism

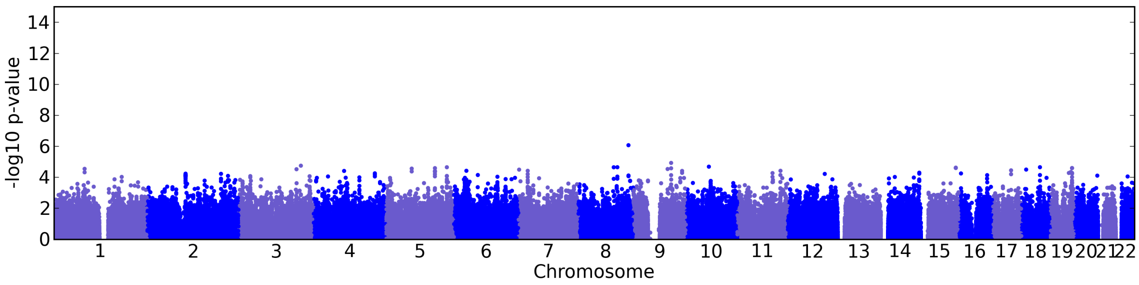

(j) Sweet tooth

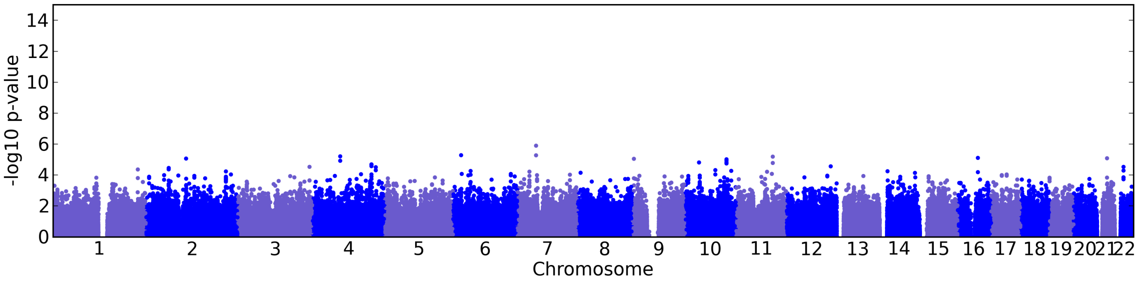

(k) Wisdom teeth

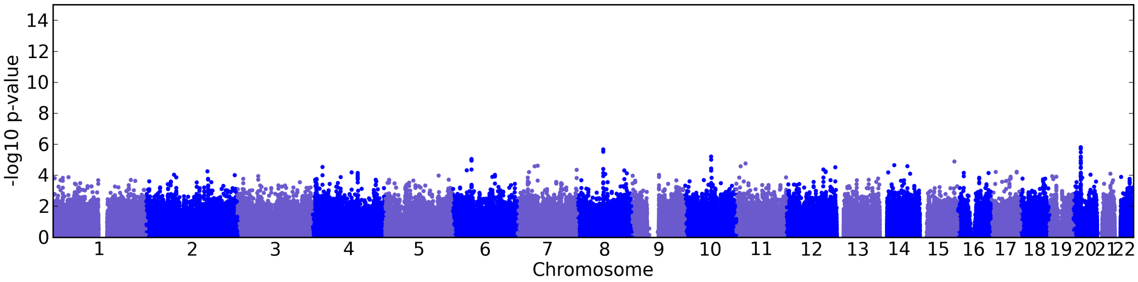

(l) Cavities

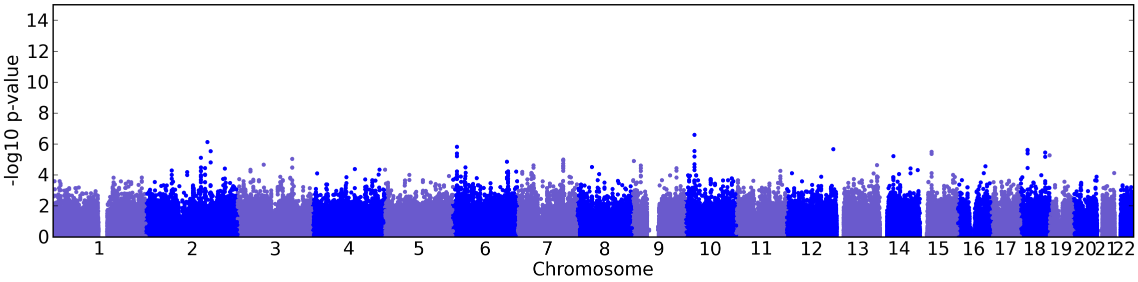

(m) Earlobe

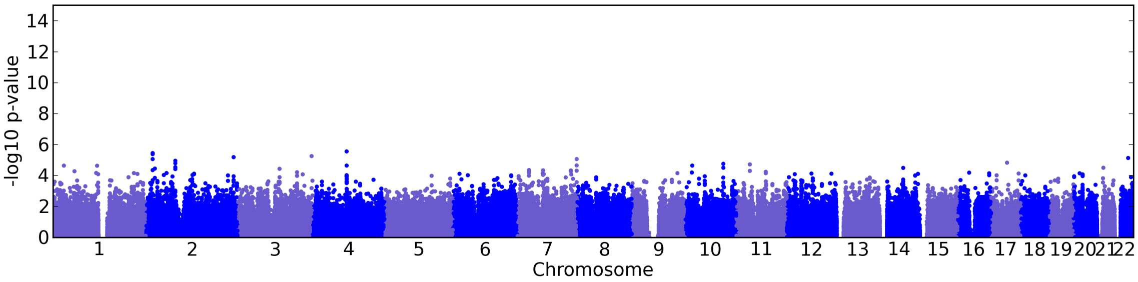

(n) Motion sickness

Figure 8. Manhattan plots for traits not included elsewhere.

References

1. Devlin B, Roeder K (1999) Genomic control for association studies. Biometrics 55: 997–1004.

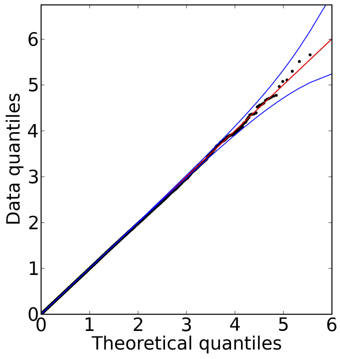

(a) Footedness

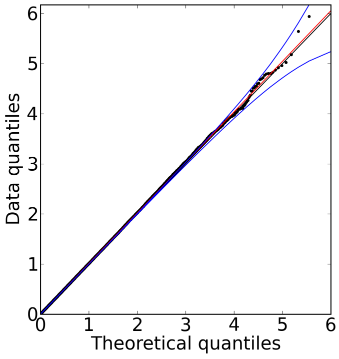

(b) Astigmatism

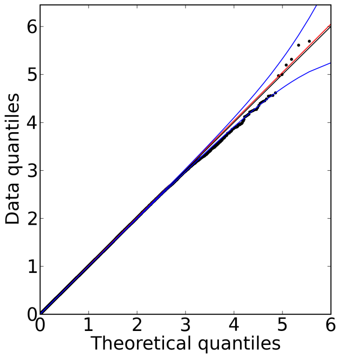

(c) Braces

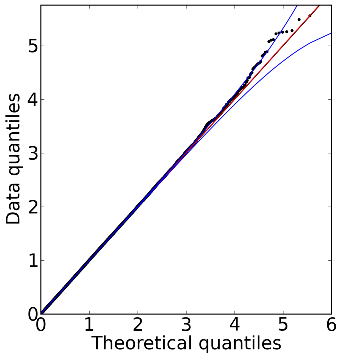

(d) Glasses

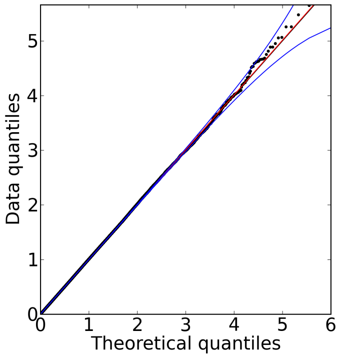

(e) Hand clasp

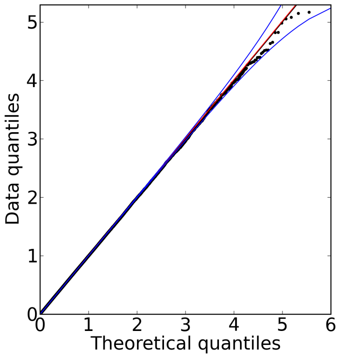

(f) Handedness

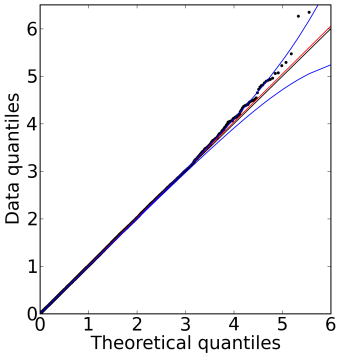

(g) Morningness

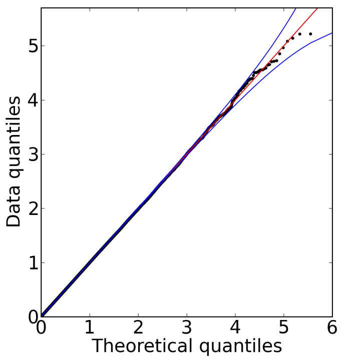

(h) Ocular dominance

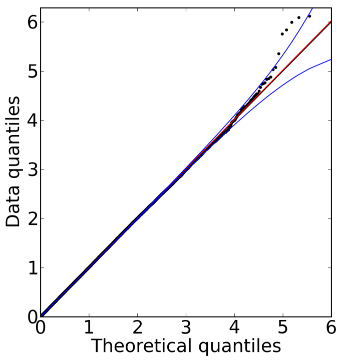

(i) Optimism

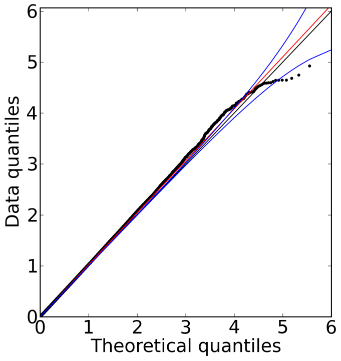

(j) Sweet tooth

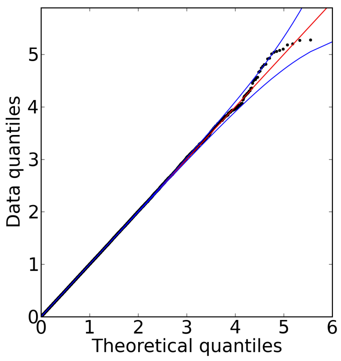

(k) Wisdom teeth

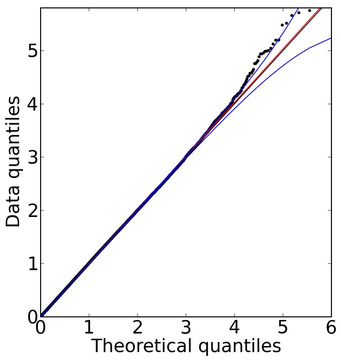

(l) Cavities

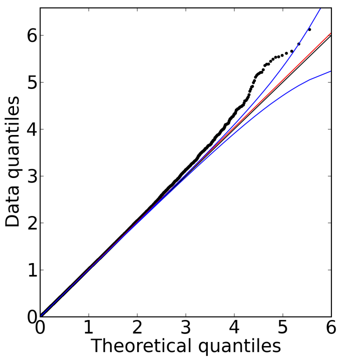

(m) Earlobe

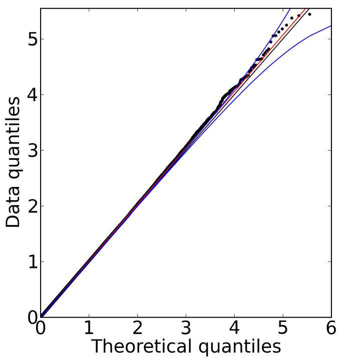

(n) Motion sickness

Figure 9. Quantile-quantile plots for traits not included elsewhere.
